# Phase-tuned modulation during reward expectancy in human anterior insular cortex

**DOI:** 10.1038/s42003-026-10427-1

**Published:** 2026-06-16

**Authors:** Linglin Yang, Katia Lehongre, Xinfeng Yu, Hongyi Ye, Vincent Navarro, Sen Cheng, Nikolai Axmacher, Shuang Wang, Hui Zhang

**Affiliations:** 1 Department of Psychiatry, Second Affiliated Hospital, School of Medicine, Zhejiang University, Hangzhou, China; 2 Department of Neurology, Epilepsy Center, Second Affiliated Hospital, School of Medicine, Zhejiang University, Hangzhou, China; 3 CENIR - Centre de Neuro-Imagerie de Recherche, Paris Brain Institute, ICM, Hôpital de la Pitié-Salpêtrière, Paris, France; 4 Department of Radiology, Second Affiliated Hospital, School of Medicine, Zhejiang University, Hangzhou, China; 5 Sorbonne Université, Inserm, CNRS, Paris Brain Institute, ICM, Hôpital de la Pitié-Salpêtrière, Paris, France; 6 Epilepsy Unit, AP-HP, PitiéSalpêtrière Hospital, Paris, France; 7 Clinical Neurophysiology Department, AP-HP, Pitié-Salpêtrière Hospital, Paris, France; 8 Institute for Neural Computation, Faculty of Computer Science, Ruhr University Bochum, Bochum, Germany; 9 Department of Neuropsychology, Institute of Cognitive Neuroscience, Faculty of Psychology, Ruhr University Bochum, Bochum, Germany; 10 State Key Laboratory of Cognitive Neuroscience and Learning and IDG/McGovern Institute for Brain Research, Beijing Normal University, Beijing, PR China

**Keywords:** Cognitive control, Reward

## Abstract

Reward expectancy engages the anterior insular cortex (AIC) to coordinate cognitive allocation. Using intracranial electroencephalographic data from epilepsy patients navigating a virtual T-maze, we identified reward-specific brain patterns (RBPs) that were preactivated in the AIC prior to reward onset. This pre-activation was followed by phase-amplitude coupling (PAC) between theta oscillations and gamma activity, with coupling strength positively correlated with the pre-activation level across contacts. Furthermore, this PAC exhibited a phase-precession-like effect (PPLE), characterized by a progressive shift of peak gamma activity to earlier theta phases across successive navigation rounds. Participants exhibiting stronger PPLE in the AIC showed greater trial-by-trial improvements in reward-collection performance. Taken together, these findings reveal a precise phase-tuned timing mechanism in the AIC that supports reward-directed behavior. Specifically, oscillatory coordination initiated by representational pre-activation, is dynamically refined through PPLE across successive exposures to the same reward. This mechanism accelerates responses to impending rewards, thereby optimizing adaptive behavior.

## Abstract (web summary)

Reward expectancy elicits pre‑activation of reward‑specific patterns in the anterior insular cortex, followed by theta‑gamma PAC. The preferred PAC phase enables temporally precise PPLE, translating expectational states into behavioral optimization.

## Introduction

Many daily behaviors of humans are driven by the expectancy of rewards, whether material or spiritual^1,2^. Reward expectancy has been proposed to boost behavioral performance^2^, particularly when the expected reward is ultimately obtained^3,4^. Research indicates that reward expectancy fundamentally depends on context-reward associations, whereby specific environmental or sensory cues become linked to rewards^5^. Recognition of reward-associated contexts (i.e., salient information) triggers activation of neural patterns encoding reward memory^3^, prompting the brain to allocate enhanced attentional and motivational resources to detect anticipated rewards^2–4,6^. Subsequent reward delivery consistent with expectations reinforces these associations via feedback-dependent plasticity^3,7,8^. These integrating processes are coordinated by the salience network^3,9,10^, within which the anterior insular cortex (AIC) serves as a pivotal hub^11–13^. Intracranial recordings in non-human primates have revealed that approximately one-quarter of responsive AIC neurons activate prior to reward delivery^14^, indicating its involvement in reward expectancy. Recent human electrophysiological studies have further confirmed the essential role of AIC in reward expectancy^10^. Nevertheless, the precise electrophysiological mechanism by which the AIC dynamically mediates reward expectancy remains poorly understood.

Intracranial electroencephalography (iEEG) provides unique access to rapid neural dynamics in deep brain regions such as the AIC^10^, revealing phase-tuned modulation mediated by neural oscillations^15,16^. A prominent form of this modulation is phase-amplitude coupling (PAC), which manifests as high-frequency activity synchronized to specific phases of low-frequency oscillations (LFOs)^17–19^, particularly theta oscillations^20–22^. Across cognitive tasks, high-frequency activity exhibit distinct phase-dependent relationships with LFOs^23,24^. For instance, gamma activity coupled to theta peaks contribute to memory encoding, whereas those coupled to theta troughs support memory retrieval^25–27^. These task-specific functional configurations demonstrate how phase-tuned modulation organizes neural computations. Notably, distinct LFO phases implement event coding through phase-locked neural patterns^16,18,21,28–32^, enabling phase-organized information binding essential for context-item association. Given the established role of PAC in information integration and context-item binding^20,22,33,34^, it is a promising candidate mechanism for mediating reward expectancy within the AIC. This functional significance is further supported by evidence linking attenuated parietal-occipital PAC to impaired reward processing in Parkinson’s disease^35^. Importantly, such decoupling results from aberrant theta phase tuning rather than decreased gamma power, highlighting the critical role of phase-tuned modulation.

Beyond PAC, more fine-grained phase-tuned modulation, specifically phase precession, may contribute to the coordination of neural oscillations during reward expectancy, given its close involvement in predicting upcoming events^36,37^. Theta phase precession, which is characterized by a progressive shift in neuronal spiking towards earlier theta phases as individuals traverse place fields, has been observed in both hippocampal place cells and entorhinal grid cells across species^36,38,39^. This phenomenon supports spatial location prediction during navigation. Furthermore, phase precession has also been observed in non-spatially tuned neurons in the human frontal cortex, indicating a broader role in predicting forthcoming events^36^. Such evolutionarily conserved phase-tuned modulation underpins working memory and predictive coding^37,40^. Notably, phase precession has been implicated in reward-related spatial encoding, potentially facilitating the precise coordination of anticipation and action. Evidence from rodent studies have demonstrated this phenomenon in salience network nodes (e.g., the ventral striatum and lateral septum) during reward expectancy, where it selectively tracks the location of rewards^41–44^. However, existing evidence for phase precession predominantly derives from microscale single-unit recordings, leaving unclear whether and how phase precession manifests at macroscale neural levels (e.g., population-level oscillations) and how such modulations relate to reward expectancy. Moreover, despite the established role of the AIC in reward expectancy, it is unclear whether the human AIC utilizes analogous phase-tuned modulation mechanisms.

To address this gap, we analyzed iEEG data from presurgical epilepsy patients performing a virtual T-maze task with pre-defined rewards. Using a combination of representational similarity analysis (RSA), PAC and phase precession analysis, we investigated AIC dynamics during the period preceding reward delivery. This study aims to advance our understanding of how neural systems optimize reward-directed behaviors through phase-tuned modulations.

## Results

**Fig. 1:**
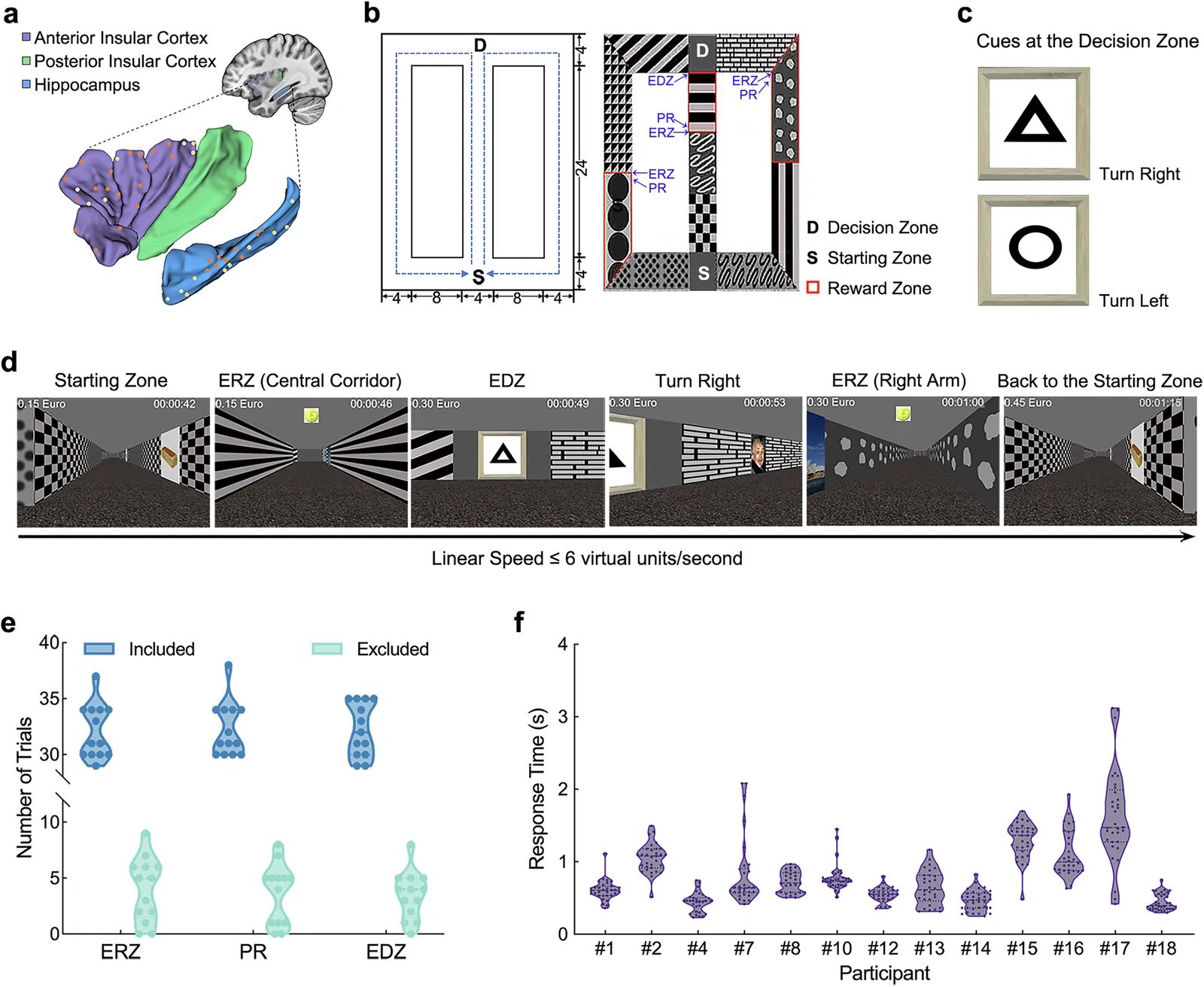
Contact locations, experimental paradigm, and behavioral performance. **a** Spatial distribution of contacts in the anterior insular cortex (purple), posterior insular cortex (green), and hippocampus (blue). Each orange sphere indicates a contact in the right hemisphere, and each yellow sphere indicates a contact in the left hemisphere. **b** Overhead view of the T maze. Left: Diagram of the virtual maze. Each blue dashed line represents one navigation round. Right: T maze adorned with various patterns on its walls. The gray square marked with ‘S’ represents the starting zone where each navigation round begins and ends, while the gray square marked with ‘D’ represents the decision zone where participants are expected to choose the correct direction according to the cue. Areas circled with red lines represent reward zones, where participants are expected to collect gold coins by pressing the “Enter” button as soon as possible. Blue arrows indicate the precise spatial locations of the ERZ (entering the reward zone) and EDZ (entering the decision zone) events, and the approximate location of the PR (picking up the reward) event. **c** Cues on the wall of the decision zone. Circle represents turning left, and triangle represents turning right. **d** An example navigation round from the first-person perspective in the maze. **e** Number of trials are similar across conditions for both included (blue) and excluded (green) trials (*n* = 13 participants). Note that there is a discontinuity in the y-axis to enhance visualization. Each dot within violin plots indicates one participant. **f** Response time for picking up rewards at the reward zone on the central corridor. Each violin plot indicates one participant, and each dot within violin plots indicates one navigation round.

To investigate the electrophysiological mechanisms of reward expectancy in the AIC, we examined 25 AIC contacts from nine epilepsy patients (18 in the right hemisphere; Fig. 1a and Supplementary Table 1). As a control, we also tested 19 hippocampal contacts (9 in the right hemisphere) from 7 participants, including 7 contacts from 3 patients with AIC contacts and 12 contacts from 4 new patients. AIC and hippocampal contacts demonstrated comparable lateralization distributions (χ^2^_(1,44)_ = 2.76, p = 0.12).

Participants navigated a virtual T-maze to collect rewards while iEEG activity was recorded (Fig. 1b-d). During each navigation round, they first entered the reward zone on the central corridor (where a reward was always delivered), and then the reward zone on a lateral arm (where reward was presented in 98.90 ± 3.28% [mean ± SD] trials following correct responses at the decision zone; see Methods). To minimize the influence of spatial heterogeneity, we focused on iEEG activity time‑locked to three key events occurring on the central corridor: entering the reward zone (ERZ, experimental condition), picking up the reward (PR, control condition), and entering the decision zone (EDZ, control condition). The number of trials included for each event was matched (32.08 ± 2.40 [mean ± SD] for ERZ, 32.23 ± 2.42 for PR, and 32.23 ± 2.39 for EDZ; F_(2,24)_ = 0.13, p = 0.79, one-way ANOVA; Fig. 1e and Supplementary Table 1). Additionally, ERZ events occurring on lateral arms (13.23 ± 3.42 for left arm, and 13.46 ± 3.93 for right arm) were included to identify reward‑specific brain patterns (RBPs) while accounting for potential spatial confounds. For each ERZ event on the central corridor, the response time (RT) for picking up the reward was calculated for further analysis (Fig. 1f).

### Pre-activation of RBPs when approaching rewards

**Fig. 2:**
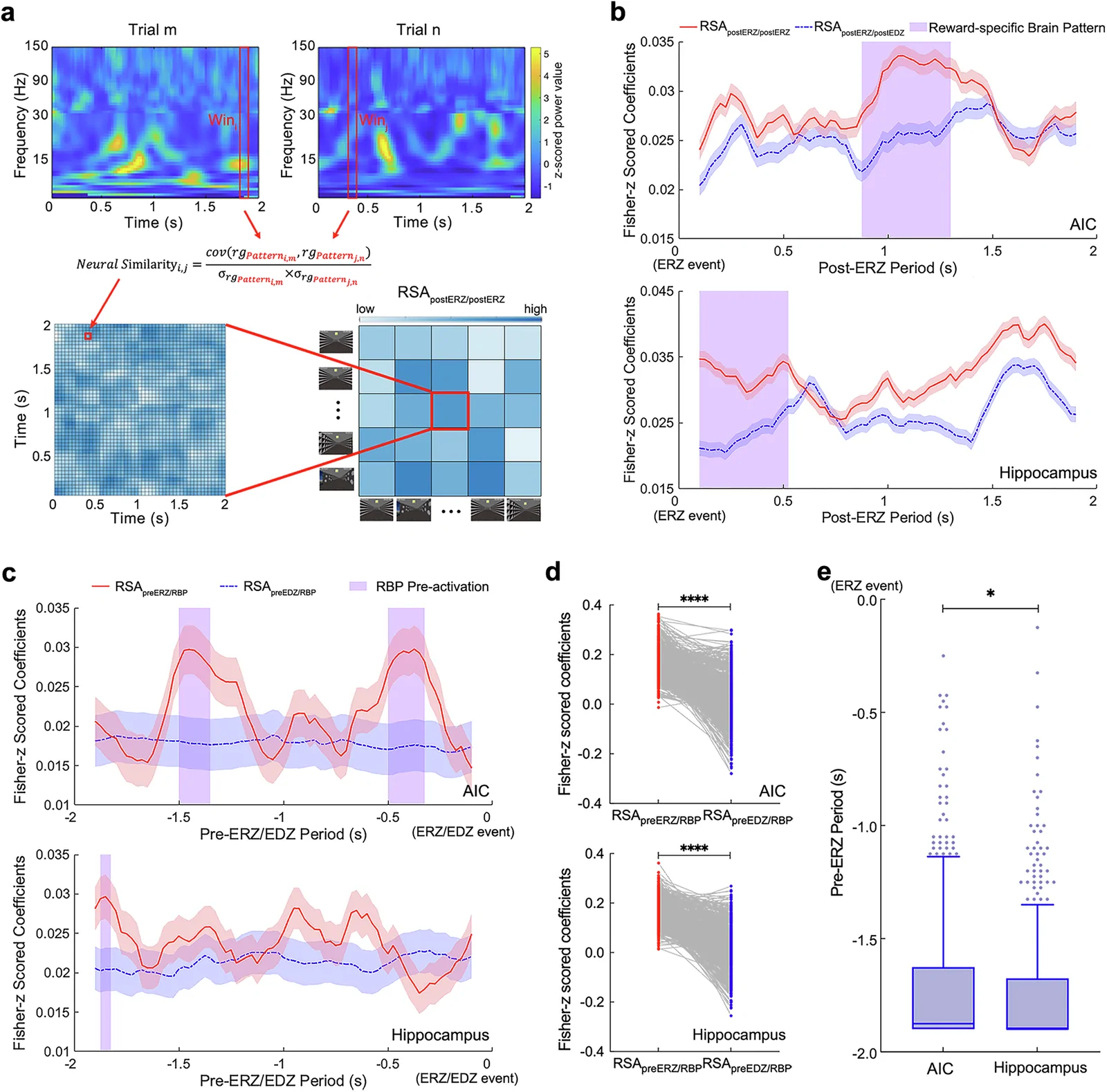
Pre-activation of RBPs before entering reward zones in the AIC and hippocampus. **a** Scheme of representational similarity analysis (RSA) to compute reward-specific brain patterns (RBPs). Representational similarity matrix illustrates the neural pattern similarities of the spectrum within each time window between different trials. Representational similarity matrix of RSA_postERZ/postERZ_ displays the categorical information structure of viewing reward at different reward zones (on the central or two lateral roads). **b** Time-resolved category specificity reveals RBPs in the anterior insular cortex (AIC; n = 25 contacts from 9 participants) and hippocampus (n = 19 contacts from 7 participants). Each line represents time courses of RSA_postERZ/postERZ_ (red; mean ± SEM) or RSA_postERZ/postEDZ_ (blue). Purple shading indicates time windows carrying RBPs (i.e., RSA_postERZ/postERZ_ > RSA_postERZ/postEDZ_). X-axis denotes time course relative to post-ERZ periods, and time 0 indicates the onset of rewards (i.e., ERZ events). ERZ, entering the reward zone; EDZ, entering the decision zone. **c** Pre-activation of RBPs before entering the reward zones in the AIC (n = 25 contacts from 9 participants) and hippocampus (n = 19 contacts from 7 participants). Each line represents time-resolved similarity trajectories for RSA_preERZ/RBP_ (red; mean ± SEM) or RSA_preEDZ/RBP_ (blue). Purple shading highlights pre-ERZ time windows for RBP pre-activation (i.e., RSA_preERZ/RBP_ > RSA_preEDZ/RBP_). X axis indicates time course relative to pre-ERZ or pre-EDZ periods, and time 0 indicates ERZ or EDZ event. **d** Different magnitude of RBPs pre-activation between RSA_preERZ/RBP_ (red) and RSA_preEDZ/RBP_ (blue) in the AIC and hippocampus. Dots represent individual trials. ****, p < 0.001. **e** The RBP pre-activations in the AIC (n = 789 trials from 9 participants) occur later than those in the hippocampus (n = 626 trials from 7 participants). Box plots show the 25^th^-75^th^ percentiles (boxes), median (line), and whiskers extending from the 5^th^ to 95^th^ percentiles; dots represent individual trials outside the whiskers. Y axis indicates time course relative to pre-ERZ periods, and time 0 indicates the onset of rewards. *, 0.01 ≤ p  < 0.05.

Reward expectancy indicates that the brain starts to process reward-relevant information before the onset of rewards. Thus, we hypothesized that the brain patterns representing rewards would be preactivated prior to actual reward delivery. To test this hypothesis, we investigated RBPs pre-activation in AIC and hippocampal contacts before reward onset using RSA^45–48^. To identify RBPs, we first computed the neural pattern similarities during reward viewing (post-ERZ periods) across different spatial locations (the central and two lateral roads; RSA_postERZ/postERZ_) to control for location-specific neural representations (Figs. 1b and 2a; see Methods). As a control comparison, we then calculated the pattern similarities between reward viewing (post-ERZ periods) and non-reward viewing periods (post-EDZ periods; RSA_postERZ/postEDZ_). Finally, we derived RBPs by contrasting these similarity measures (RSA_postERZ/postERZ_ vs. RSA_postERZ/postEDZ_) during post-ERZ periods (Fig. 2b), ensuring the identified patterns specifically reflected reward processing rather than spatial or task-general neural activities. Significant RBPs emerged in both regions during reward viewing. In the AIC, neural activity within 0.875-1.3 seconds following reward onset showed significantly higher neural pattern similarity for reward viewing pairs (RSA_postERZ/postERZ_) compared to reward viewing and non-reward viewing pairs (RSA_postERZ/postEDZ_, p_corrected_ = 0.047). In contrast, the hippocampus encoded reward information earlier, with enhanced similarity for reward viewing (RSA_postERZ/postERZ_) versus reward viewing and non-reward viewing pairs (RSA_postERZ/postEDZ_) emerging within 0.1-0.525 seconds following reward onset (p_corrected _= 0.033). Further control analyses using RSA supported that the identified RBPs represent reward-specific activity rather than motor preparation (Supplementary Note 1 and Supplementary Fig. 1).

To examine the pre-activation of RBP, we computed the neural similarities between each time window before reward onset (pre-ERZ periods) and each time window showing RBPs when viewing rewards (post-ERZ periods; RSA_preERZ/RBP_). To control for location-specific effects, we compared brain pattern from pre-ERZ periods at the central road and post-ERZ periods at the lateral roads. To avoid the temporal proximity effect (i.e., autocorrelations), correlations between pre- and post-ERZ periods from the same navigation round were excluded. As a control measure, we calculated the neural similarities between the time windows before the display of decision signs (pre-EDZ periods) and time windows showing RBPs when viewing rewards (post-ERZ periods; RSA_preEDZ/RBP_). The results revealed distinct temporal profiles between the AIC and hippocampus (Fig. 2c). Strikingly, a temporally refined analysis revealed two different time windows of RBP pre-activation in the AIC before ERZ events: one between -1.5 and -1.35 seconds and another between -0.5 and -0.325 seconds (p_corrected_s < 0.001). During these time windows, the RSA_preERZ/RBP_ values were significantly higher than the average RSA_preEDZ/RBP_ values. The hippocampus exhibited an earlier RBP pre-activation window between -1.875 to -1.825 seconds (p_corrected _< 0.001). To quantify the magnitude of RBPs pre-activation, we extracted the maximum neural pattern similarity values (RSA_preERZ/RBP_ and RSA_preEDZ/RBP_) within individual trials, separately for the AIC and hippocampus. In the AIC, the magnitudes of RBPs pre-activation during pre-ERZ periods significantly exceeded those during pre-EDZ periods, both at the trial level (0.17 ± 2.15×10^-3^ [mean ± SEM] vs. 0.02 ± 3.65×10^-3^, t_(1526)_ = 36.33, p < 0.001; independent t-test; Fig. 2d) and the contact level (0.17 ± 9.62×10^-3^ vs. 0.02 ± 4.63×10^-3^, t_(24)_ = 15.94, p < 0.001; paired t-test; Supplementary Fig. 2a). A highly similar effect was observed in the hippocampus, again at both the trial (0.17 ± 2.30×10^-3^ vs. 0.02 ± 3.80×10^-3^, t_(1264)_ = 33.54, p < 0.001; Fig. 2d) and contact levels (0.17 ± 0.01 vs. 0.02 ± 4.39×10^-3^, t_(18)_ = 13.81, p < 0.001; Supplementary Fig. 2b). We further examined the temporal sequence of RBP pre-activation between the AIC and hippocampus. The results showed that the hippocampus exhibited significantly earlier initial RBP pre-activation (-1.80 ± 0.01 seconds [mean ± SEM]) compared to the AIC (-1.70 ± 9.70×10^-3 ^seconds, p = 0.028; Mann-Whitney test; Fig. 2e).

Taken together, these results demonstrate that robust RBP pre-activation occurred prior to reward delivery, exhibiting distinct region-specific temporal dynamics. This spatiotemporally precise pre-activation underscores conserved reward-predictive mechanisms across brain regions, suggesting that the brain started to process reward information during reward expectancy.

### Coupling between theta phases and gamma amplitudes in the AIC when approaching rewards

**Fig. 3:**
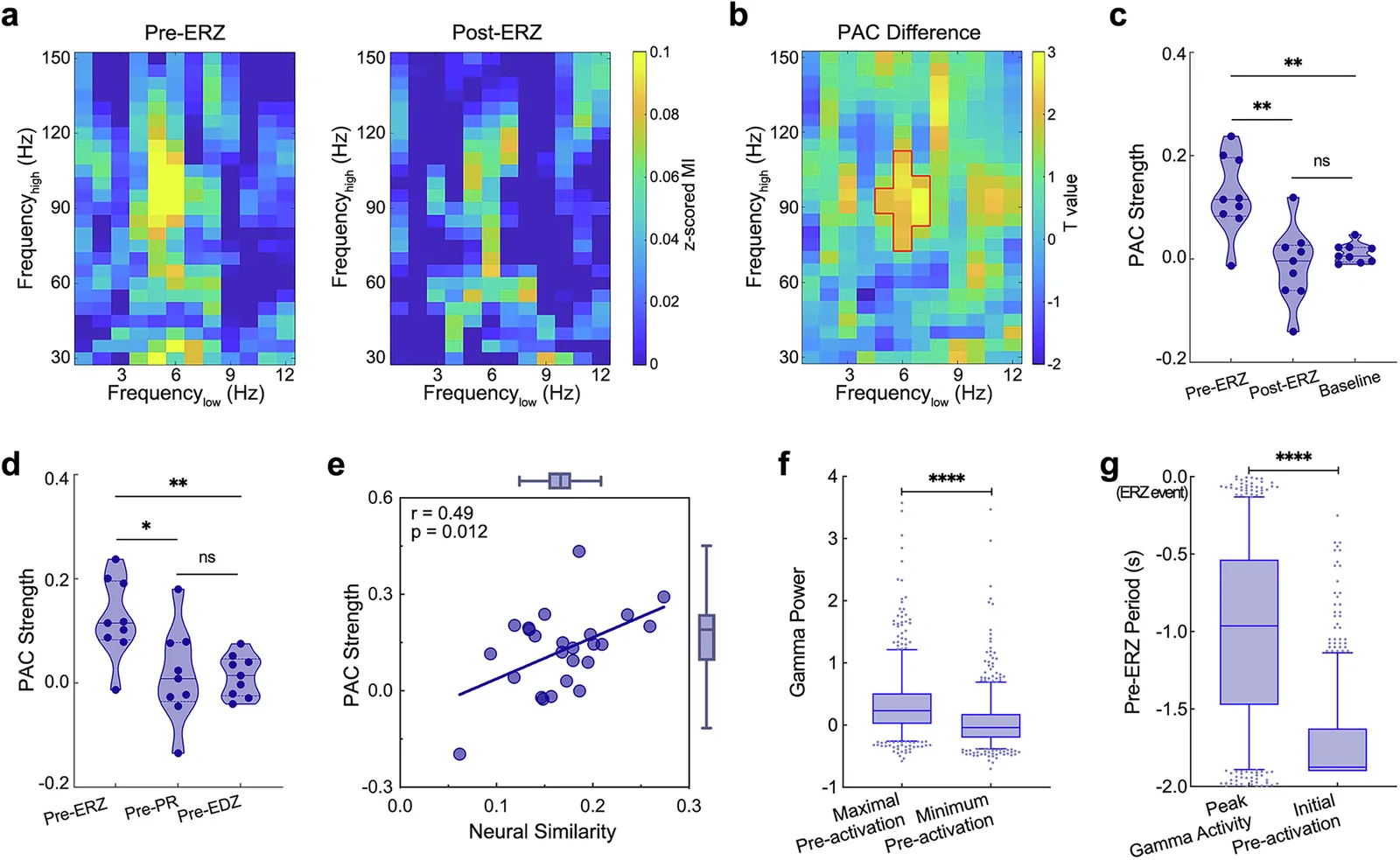
Theta-gamma PAC and its relationship to RBP pre-activation in the AIC before entering reward zones. **a** Comodulograms illustrate the z-scored modulation index (MI) during the pre-ERZ and post-ERZ periods. ERZ, entering the reward zone (n = 25 contacts from 9 participants). **b** Difference in phase-amplitude coupling (PAC) between pre-ERZ and post-ERZ periods. Red contours indicate a frequency_low_-frequency_high_ cluster showing significantly enhanced PAC during pre-ERZ relative to post-ERZ periods. **c** Average PAC values within the frequency_low_-frequency_high_ cluster from panel b for individual participants in the pre-ERZ, post-ERZ, and baseline conditions. Each violin plot indicates one condition, and each dot within violin plots indicates one participant. **, 0.005 ≤ p < 0.01; ns, no significant difference. **d** Average PAC values within the identified cluster from panel b for individual participants in the pre-ERZ, pre-PR, and pre-EDZ conditions. Each violin plot indicates one condition, and each dot within violin plots indicates one participant. *, 0.01 ≤ p  < 0.05, **, 0.005 ≤ p < 0.01; ns, no significant difference. **e** The PAC strength positively correlate with the neural similarities of pre-ERZ periods relative to reward-specific brain patterns (RBP) across contacts. Each sphere indicates one recording contact, and the blue line indicates the fitted line. Blue box plots indicate the distribution of PAC strength or neural similarities. **f** Gamma power showing PAC effects is higher at the time bins with maximal RBP pre-activation than those with minimum pre-activation (n = 791 trials). Box plots show the 25^th^-75^th^ percentiles (boxes), median (line), and whiskers extending from the 5^th^ to 95^th^ percentiles; dots represent individual trials outside the whiskers. ****, *p* < 0.001. **g** Peak gamma activity showing PAC effects occur later than initial RBP pre-activation (n = 791 trials). Box plots show the 25^th^-75^th^ percentiles (boxes), median (line), and whiskers extending from the 5^th^ to 95^th^ percentiles; dots represent individual trials outside the whiskers. Y axis indicates the time course relative to pre-ERZ periods, and time 0 indicates the onset of rewards. ****, p < 0.001.

To characterize the neural dynamics underlying reward expectancy, we computed the difference in PAC strength between the pre- and post-ERZ periods (see Methods). Firstly, we extracted the time courses of phases for each low-frequency band (frequency_low_;1-12 Hz in 1-Hz steps) and amplitude for each high-frequency band (frequency_high_; 30-150 Hz in 5-Hz steps). Utilizing an established method^49^, PAC strength was subsequently calculated for each frequency_low_-frequency_high_ pair for each pre- and post-ERZ periods (Fig. 3a). Differences in PAC strength between pre- and post-ERZ periods were examined using a multi-level linear regression followed by a cluster-based permutation analysis^50,51^. We observed significantly stronger coupling between theta phases (5-7 Hz) and gamma amplitudes (75-110 Hz) in the AIC during pre-ERZ periods than during post-ERZ periods (p_corrected _= 0.005; Fig. 3b). Identical analyses revealed no significant difference in PAC strength between before and after PR/EDZ events in the AIC (Supplementary Fig. 3). Similarly, no significant difference in PAC strength was observed between the periods before and after any events (ERZ/PR/EDZ) in the hippocampus (Supplementary Fig. 4).

To investigate whether the significant frequency_low_-frequency_high_ cluster (Fig. 3b) was due to the enhanced PAC effect during pre-ERZ periods, we extracted the average PAC values within the identified cluster separately for pre-ERZ, post-ERZ, and baseline condition which was randomly selected 2-second data outside of ERZ events. We found in general, the PAC value was different between pre-ERZ, post-ERZ, and the baseline (F_(2,16)_ = 11.02, p = 0.004; one-way ANOVA following by post-hoc paired t-test; Fig. 3c). Specifically, the PAC strength during the pre-ERZ period (0.12 ± 0.08 [mean ± SD]) was significantly greater than that observed during the post-ERZ period (-0.01 ± 0.07, t_(8)_ = 3.39, p = 0.01) and the baseline (0.01 ± 0.02, t_(8)_ = 4.47, p = 0.002). No significant difference in PAC strength was found between the post-ERZ period and the baseline (t_(8)_ = 0.91, p = 0.39). Additionally, we found that the PAC strength is different among the pre-ERZ, pre-PR, and pre-EDZ periods (F_(2,16)_ = 8.72, p = 0.006; Fig. 3d). Further analysis showed that the theta-gamma PAC effect was stronger during the pre-ERZ period than during the pre-PR (0.02 ± 0.09, t_(8)_ = 3.01, p = 0.017) and pre-EDZ (0.01 ± 0.04, t_(8)_ = 3.62, p = 0.007) periods. These results suggest that the enhanced PAC effect in the AIC reflects a genuine increase in coupling strength during pre-ERZ periods, rather than a mere consequence of reduced PAC strength during post-ERZ periods. We further ruled out the possibility that this PAC enhancement was confounded by fluctuations in gamma amplitude (Supplementary Note 2 and Supplementary Fig. 5).

Subsequently, we investigated the relationship between PAC dynamics and RBP pre-activation preceding reward onset. The result showed that the PAC strength was positively correlated with the level of RBP pre-activation across all AIC contacts (r = 0.49, p = 0.012, Pearson’s correlation; Fig. 3e). To further verify the relation between PAC strength and RBP pre-activation level, we compute the correlation across trials within individual contacts. A significant positive correlation was observed in 15 out of 25 contacts (Supplementary Fig. 6), a proportion significantly exceeding chance level (i.e., 5%; p = 5.97×10^-14^; Binomial test). It indicates that the group-level effect was supported by the majority of recording contacts, while also revealing some contact-specific variability. Further data analysis showed that the gamma power (75-110 Hz) was significantly elevated during time windows with maximal RBP pre-activation (0.33 ± 0.017 [mean ± SEM]) compared to those with minimum pre-activation (0.04 ± 0.014, p < 0.001; Wilcoxon matched-pairs signed rank test; Fig. 3f), suggesting that the RBP pre‑activation is accompanied by increased gamma‑band activity. These results demonstrate a positive association between PAC strength and RBP pre-activation levels.

Additionally, we examined the temporal relationship between the peak theta-gamma PAC effect and the onset of RBP pre-activation. The peak PAC effect for each pre-ERZ period was defined as the emergence of peak gamma activity with the maximal amplitude at the preferred PAC phase (see Methods). Across all trials and contacts, the peak gamma activity occurred markedly later than the initial RBP pre-activation (-1.00 ± 0.02 seconds [mean ± SEM] vs. -1.70 ± 9.7×10^-3 ^seconds, p < 0.001; Wilcoxon matched-pairs signed rank test; Fig. 3g), with a mean time lag of 0.73 ± 0.02 seconds. The coefficient of variation (CV, 85.77%) indicated a high dispersed distribution of the time lag. Detailed analyses at the individual contact, contact‑averaged, and participant levels, along with corresponding statistics, can be found in Supplementary Note 3 (Supplementary Figs. 7 and 8, and Supplementary Table 2). Together, these findings demonstrate that RBP pre‑activation consistently precedes the peak of theta‑gamma oscillatory coordination during the pre‑ERZ period, but the interval between the two events is highly variable, suggesting that the precise timing of their interaction may instead be modulated by ongoing cognitive demands.

Taken together, these results suggested that gamma-band activity coupled to theta oscillations when approaching rewards, particularly in the AIC. The consistent temporally sequential relationship between PAC dynamics and RBP pre-activation further suggests the possible role of pre-activation of RBPs in anticipating the emergence of coordinated theta-gamma activity during reward expectancy.

### Gamma activity exhibit phase-precession-like effect (PPLE) in the AIC when approaching rewards

**Fig. 4:**
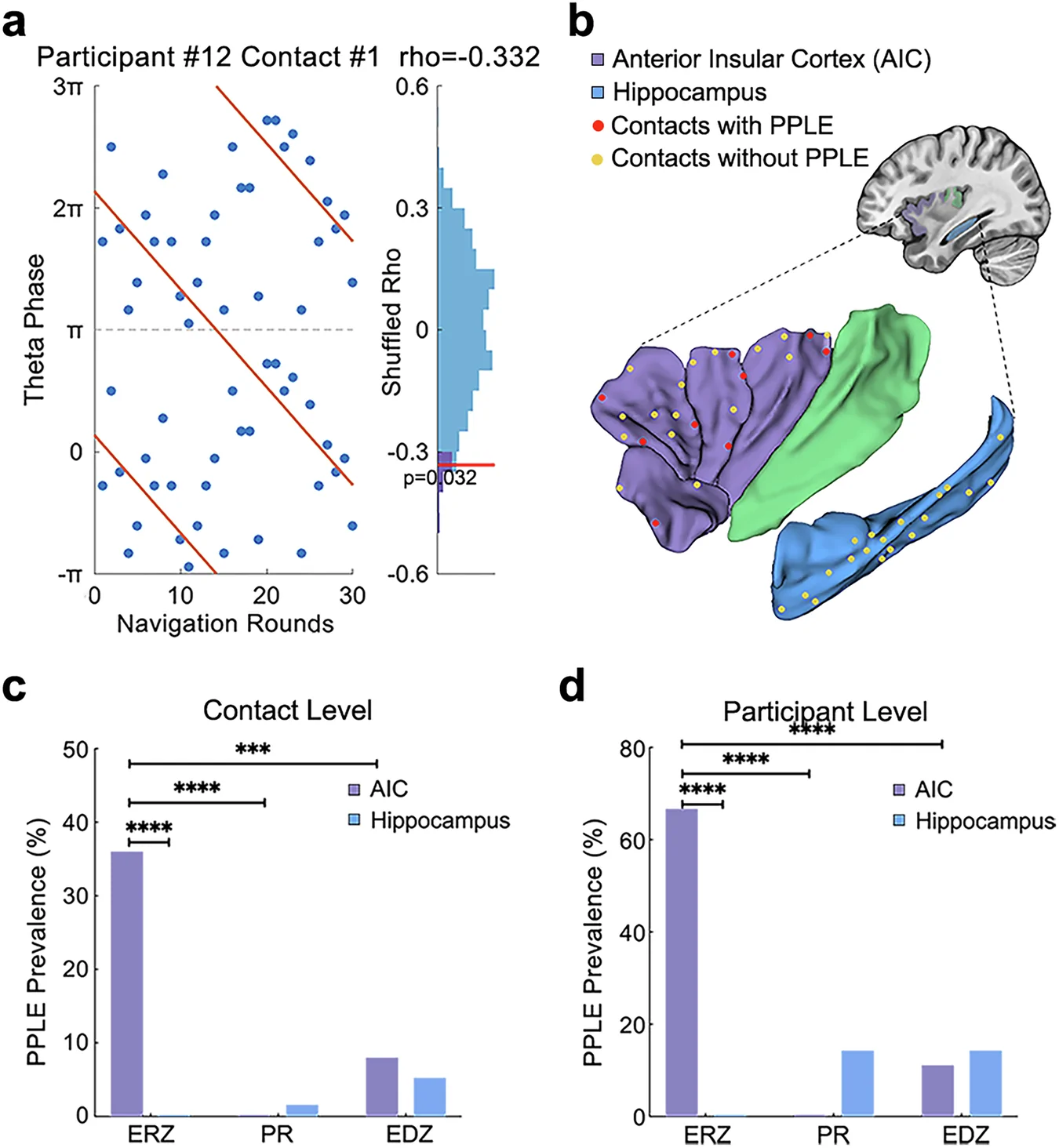
PPLEs in the AIC before entering the reward zones. **a** An example demonstrates that peak gamma activity is locked to earlier theta phases over navigation rounds when participants are approaching the same reward zone. Blue spheres indicate individual rounds, and orange lines indicate the fitted line between theta phases corresponding to peak gamma activity and navigation rounds. Note that theta phases are duplicated vertically to enable visualization of circular-linear regression. The statistical assessment of circular-linear regression coefficients (rho) is conducted using surrogate distribution of rho values generated by shuffling round labels (the blue bar plot on the right). Red line indicates rho value of real data, and purple shading indicates the 95^th^ percentile of the surrogate distribution. **b** Spatial distribution of contacts showing phase-precession-like effects (PPLEs) in the anterior insular cortex (AIC; n = 25 contacts from 9 participants) and hippocampus (n = 19 contacts from 7 participants). **c**,** d** Prevalence of PPLE in the AIC (purple) and hippocampus (blue) for each condition at the contact (**c)** and participant (**d)** levels. ERZ, entering the reward zone; PR, picking up the reward; EDZ, entering the decision zones. *, 0.01 ≤ p < 0.05; **, 0.005 ≤ p < 0.01; ***, 0.001 ≤ p < 0.005; ****, p < 0.001.

By visual inspection, we found that the preferred PAC phase demonstrated a trial-to-trial variability within individual recording contacts (Fig. 4a and Supplementary Fig. 9), reflecting dynamic reconfiguration of phase-amplitude coordination across behavioral epochs. We therefore aimed to investigate if gamma activities were coupled to theta phases that systematically shifted forward or backward when participants were approaching a reward. To this end, we investigated the dynamic pattern of preferred theta phases across navigation rounds by performing a circular-linear correlation analysis within individual AIC contacts (see Methods)^36^. We found a phase-tuned effect showing significant negative correlation in 9 out of 25 (36%) AIC contacts from 6 out of 9 (66.7%) participants, indicating progressive advancement of peak gamma activity to earlier theta phases when approaching the same reward on the central corridor. This phase-tuned modulation was reminiscent of phase precession effect and termed PPLE. This phenomenon was absent in the remaining 16 AIC contacts, with no significant correlations observed (Fig. 4b and Supplementary Fig. 9a). The prevalence of PPLE significantly exceeded the chance level (i.e., 5%) at both the contact and participant levels (ps ≤ 10^-6^; Binomial test), confirming the neurophysiological relevance of PPLE. To exclude the systematic temporal shifts in peak gamma activity as a confounding factor for PPLE, we examined the linear correlation between the timepoints corresponding to peak gamma activity and the number of navigation rounds (Supplementary Fig. 10; see Methods). No AIC contacts exhibited significant negative correlations (βs ≥ -0.35, p_corrected_s ≥ 0.064), while one recording contact from participant #16 showed a positive correlation (β = 0.40, p_corrected_ = 0.036). It argues against systematic temporal forward shift in gamma activity as the driver of PPLE, suggesting that PPLE primarily reflects an internal neural activity associated with reward expectancy, rather than being driven by the estimating the timing of reward onsets.

The PPLE in the AIC when approaching rewards was possibly the electrophysiological foundation of reward expectancy. Meanwhile, it might also relate to the expectancy of visual stimuli, specific locations, or outcomes of picking up rewards. To determine whether PPLE in the AIC was specifically related to reward expectancy, we compared PPLE prevalence during pre-ERZ periods with two control conditions (i.e., pre-PR or pre-EDZ periods; Fig. 4c and d, Supplementary Figs. 11 and 12). PPLE occurred in fewer AIC contacts during pre-PR (0/25) and pre-EDZ (2/25) periods which were significantly smaller than during pre-ERZ periods (pre-PR: p = 1.4 × 10^-5^; pre-EDZ: p = 1.6 × 10^-3^; Binomial test). At the participant level, a similar pattern was observed, demonstrating a lower occurrence of PPLE during pre-PR (0/9 cases, p = 4.6 × 10^-5^) and pre-EDZ (1/9 cases, p = 8.9 × 10^-4^) periods in comparison to pre-ERZ periods. These findings suggest that PPLE in the AIC is preferred to reward expectancy rather than reward outcomes (i.e., pre-PR periods) or non-reward visual stimuli or specific locations (i.e., pre-EDZ periods).

To determine if PPLE prior to reward appearance is specific to the AIC, we examined PPLE prevalence in hippocampal contacts during pre-ERZ periods (Figs. 4b-d and Supplementary Fig. 9b). None of the hippocampal contacts (0/19) exhibited PPLE, showing significantly lower prevalence compared to the AIC contacts (p = 2.1 × 10^-4^; Binomial test). This dissociation maintained statistical significance at the participant level: no participants (0/7) with hippocampal contacts exhibited PPLE, whereas 66.7% (6/9) participants with AIC contacts did (p = 4.3 × 10^-4^). It suggests that PPLE during pre-ERZ periods is more prevalent in the AIC rather than in the hippocampus.

Subsequently, we examined whether PPLE strength correlated with either average PAC strength or average RBP pre-activation level across contacts in the AIC during the pre-ERZ period. PPLE strength was quantified as the absolute value of the circular-linear coefficient for each contact. The result showed that the PPLE strength was independent of both average PAC strength (r = -0.09, p = 0.67; Spearman’s correlation) and average RBP pre‑activation level (r = -0.25, p = 0.24; Supplementary Fig. 13). These results indicate that PPLE, characterized by a progressive shift in PAC phase across rounds, represents a distinct phenomenon from both the enhanced theta-gamma PAC and the earlier RBP pre‑activation.

Taken together, PPLE reveals that gamma activity peaks at progressively earlier theta phases as participants approach the same rewards across successive navigation rounds. Furthermore, such a reward expectancy-related PPLE is especially prominent in the AIC.

### Functional relevance of PPLE

**Fig. 5:**
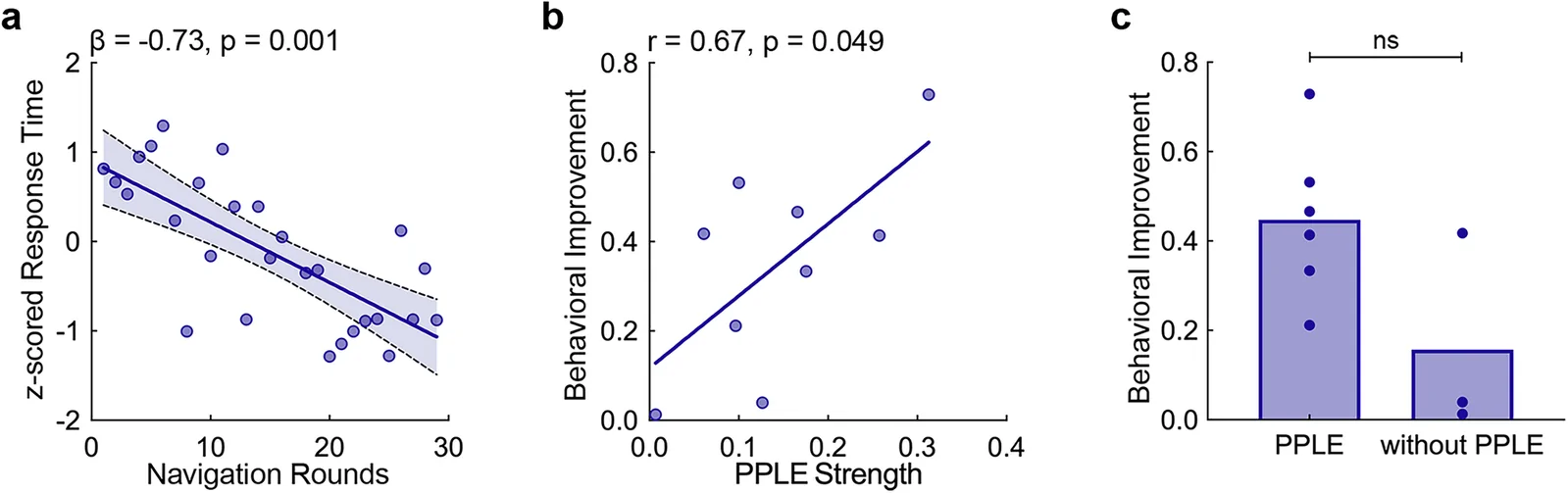
Functional relevance of PPLEs. **a** An example showing that response time (RT) decreases with an increasing number of navigation rounds. Each sphere indicates one navigation round, blue line indicates the fitted line between z-scored RT and navigation rounds, and shaded area represents 95% confidence interval of the fitted line. **b** Behavioral performance improved greater for participants (n = 9) with stronger phase-precession-like effects (PPLEs). Each sphere indicates one participant, and blue line indicates the fitted line between the magnitude of behavioral improvement and PPLE strength. **c** No significant difference in the degree of behavioral improvement was found between participants with and without PPLE. Each sphere represents one participant.

Reward anticipation has been shown to enhance cognitive performance, such as shortening response latency^6,52^. We thus investigated whether PPLE facilitated participants’ responses during reward expectancy. To examine trial-wise behavioral improvement, we analyzed z-scored RTs after excluding outliers (see Methods). A decreasing trend in RT across navigation rounds was observed in all nine participants with AIC contacts (βs < 0; Fig. 5a and Supplementary Fig. 14), which was statistically reliable in six participants as confirmed by surrogate analysis (ps < 0.05). For each participant, the behavioral improvement was quantified by the absolute regression coefficient of z-scored RT across navigation rounds.

We next evaluated the role of PPLE. We computed the PPLE strength for each participant as the average absolute value of circular-linear coefficients across AIC contacts. Across participants, the PPLE strength was positively correlated with the degree of behavioral improvement (r = 0.67, p = 0.049; Fig. 5b). To address the potential confounding factor of general practice, we performed a partial correlation analysis controlling for navigation rounds. The association remained substantial in effect size (r = 0.66), yet it was not statistically significant (p = 0.076). While this indicates the relationship may not be solely attributed to time-on-task effects, the interpretation requires caution due to the small number of participants. Additionally, participants with a detectable PPLE (n = 6) showed numerically higher but statistically comparable behavioral improvement to those without (0.45 ± 0.18 [mean ± SD] vs. 0.16 ± 0.23, p = 0.17; Wilcoxon matched-pairs signed rank test; Fig. 5c). These results suggest that the strength, rather than the presence, of PPLE is related to behavioral improvement. Further studies with larger sample sizes are needed to investigate the functional relevance of PPLE strength. Additionally, we performed complementary analyses to examine whether behavioral improvement correlated with RBP pre‑activation level or PAC strength (Supplementary Note 4; Supplementary Fig. 15a and b).

To determine whether the PPLE was specifically related to dynamic behavioral optimization rather than overall response speed, we tested its correlation with average RT across participants. The PPLE strength showed no significant correlation with average RT, either before (r = -0.25, p = 0.52; Supplementary Fig. 15c) or after controlling for navigation rounds (r = -0.18, p = 0.66). This dissociation indicates that the PPLE strength tracks trial-by-trial behavioral improvement, rather than stable individual differences in general performance.

Taken together, these findings suggest that reward-directed behavioral improvement is associated with a precise phase‑tuned timing mechanism (e.g., PPLE). To be more specific, a stronger PPLE is associated with a progressive reduction in RT toward the upcoming reward across successive navigation rounds, underscoring its role in trial‑by‑trial behavioral optimization.

## Discussion

**Fig. 6:**
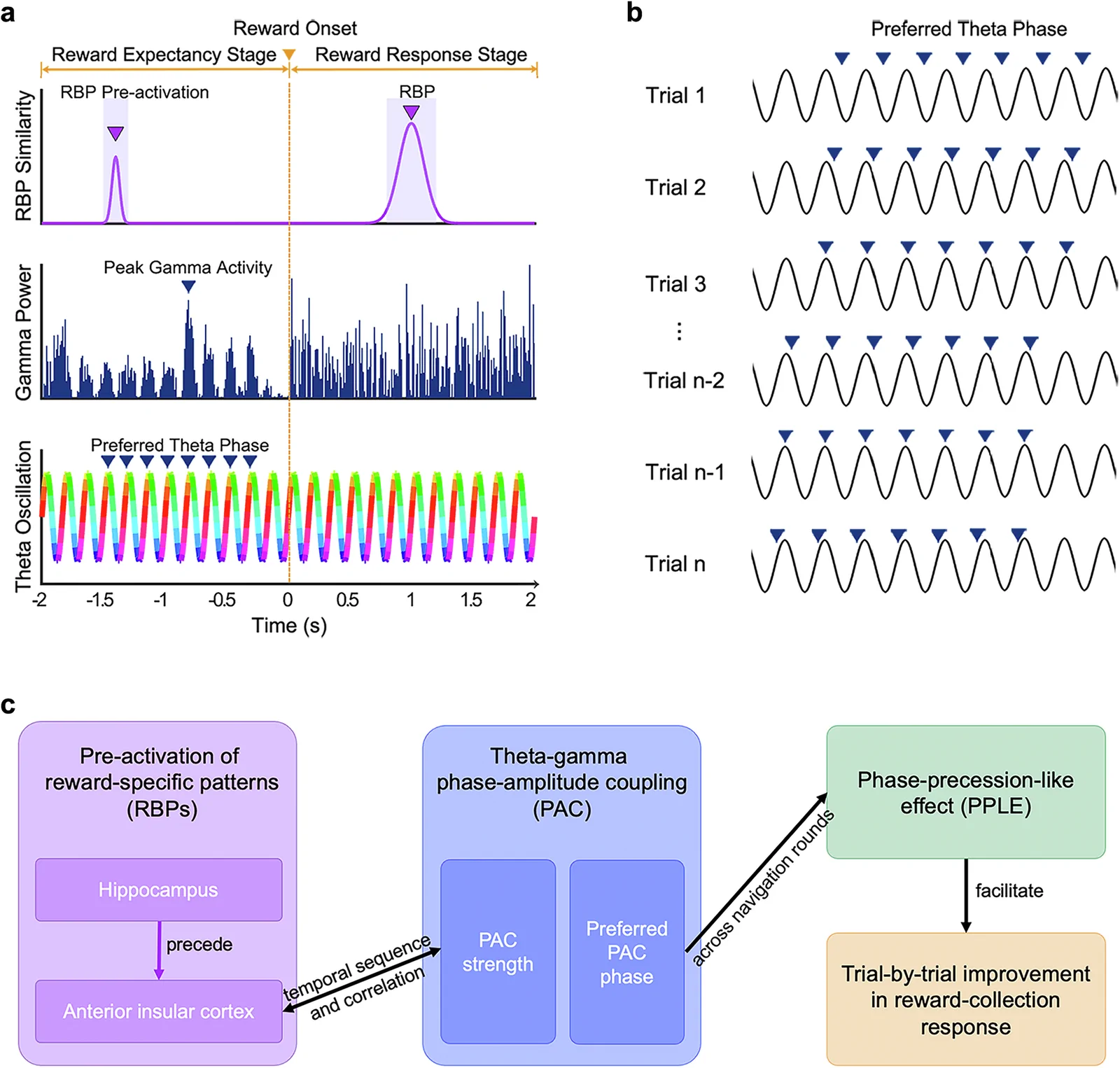
Dual phase-tuned modulation mechanisms underlying reward expectancy in the AIC. **a** Schematic of single-trial neural dynamics. Top: The reward-specific brain pattern (RBP, purple trace) shows anticipatory pre-activation (shaded area) during the reward expectancy stage, peaking at the critical timepoint (purple triangle). Middle: Gamma-band power (blue bars) exhibits phase-tuned modulation by theta oscillations. Peak gamma activity (blue triangle), which is coupled to the preferred theta phase, emerges after the RBP pre-activation. Bottom: Theta oscillations with continuous phase encoding (0-2π gradient color map). Blue triangles mark the preferred phases of phase-amplitude coupling (PAC). **b** Trial-to-trial phase-precession-like effect (PPLE). Gamma activity are coupled with advanced theta phases (blue triangles) across trials. Individual trials demonstrate progressive phase advancement of theta-gamma PAC (blue triangles) across successive reward experiences. **c** PAC serves as a critical bridge in two senses: its strength links back to representational content (RBP pre-activation), reflecting whether oscillatory coordination is engaged; its phase links forward to behavioral outcomes via PPLE, determining how that coordination is temporally organized to support behavioral optimization.

Reward expectancy has been demonstrated to facilitate critical cognitive processes during goal-directed behavior, including attention, memory, and motivation^2,4,6^. To elucidate its neural substrate, we analyzed iEEG activity in the AIC as participants approached pre-determined reward zones in a virtual T-maze task, revealing several key findings. Firstly, we identified RBPs in the AIC that exhibited pre-activation prior to reward onset. Subsequent observation revealed enhanced theta-gamma PAC, the peak of which occurred following the rise of RBP pre-activation in most recording contacts. Notably, at the group level, PAC strength exhibited a positive correlation with the degree of RBP pre-activation. Furthermore, the peak gamma activity derived from PAC analysis progressively advanced toward earlier theta phases across successive navigation rounds, a phenomenon we term PPLE. Behaviorally, stronger PPLE was associated with more pronounced trial-by-trial improvements in reward-collection response. Collectively, these results delineate a dynamic temporal structure that supports an integrated “phase-tuned modulation” model in which theta-gamma PAC serves as a critical intermediary linking representational content to behavioral outcomes through two functionally distinct characteristics (Fig. 6). In this framework, PAC strength reflects the engagement of oscillatory dynamics following representational (RBP) pre-activation, while the preferred PAC phase provides temporally precise coding manifesting as PPLE that translates expectational states into behavioral optimization. This framework highlights phase-tuned oscillatory dynamics (e.g., PAC and PPLE) as candidate electrophysiological mechanisms through which the AIC facilitate reward expectancy.

### Temporal dynamics of reward expectancy in the AIC

Neuroimaging research has implicated the AIC in the initial stages of reward processing^53–55^. An invasive investigation in non-human primates has also identified that distinct AIC neuronal populations activate in response to immediate rewards, indicating the role of AIC in anticipating rewards^14^. Complementing these findings, human research has revealed temporally structured neural activity within the AIC underlying reward prediction^10^.

Our results further advance this understanding by delineating a temporally precise mechanism. We observed robust pre-activation of RBPs in the AIC during reward expectancy, consistent with the SOCRATIC model^56^. This model posits that reward-related memories (conceptualized as RBPs in our study) are retrieved in familiar reward-associated contexts. Importantly, our data provide empirical support for the subsequent integration stage proposed by the model. According to the SOCRATIC framework, preactivated mnemonic representations (e.g., prior reward experience) must integrate with ongoing sensory inputs (e.g., current contextual cues) to generate precise reward expectations^3,12,56–58^. The model further posits that theta-gamma coding, such as PAC, mediates this integration by compressing real-world events into neural timescales^56,59,60^. In line with this perspective, we observed enhanced PAC between theta oscillations (5-7 Hz) and gamma activity (75-110 Hz) in the AIC, following RBP pre-activation. The significant positive correlation between PAC strength and RBP pre-activation magnitude at the group level suggests that preactivated mnemonic information may scaffold theta-gamma synchronization to amplify reward-predictive signals. Collectively, these findings indicate a potential sequence in which preactivated RBPs provide the representational content (“what”), and subsequent theta-gamma PAC in the AIC contributes to the temporal structure (“when” and “how”) of reward expectancy.

The integration process mediated by theta-gamma PAC is strongly modulated by dopaminergic pathways^56,60^. The critical role of dopamine is illustrated in Parkinson’s disease, where dopaminergic degeneration leads to theta-gamma decoupling and impaired reward processing^35^. Specifically, these deficits can be partially remedied by dopaminergic medication^35^. Given its rich dopaminergic innervation, the AIC integrates bottom-up sensory inputs (e.g., context cues) with top-down anticipatory signals (e.g., preactivated RBPs) through dopamine-modulated PAC, thereby dynamically optimizing the allocation of cognitive resource towards expected rewards^60–62^.

Additionally, we found that RBP pre-activation occurs earlier in the hippocampus than in the AIC, suggesting a potential role for the hippocampus in the initial retrieval of contextual information. The present study proposes that this early engagement of hippocampus provides a generalized contextual scaffold to the AIC, thereby enabling subsequent specialized integration of reward-specific information. Concurrently, the enhancement of PAC prior to reward onset was observed in the AIC, but not the hippocampus. This dissociation may be indicative of distinct dopaminergic mechanisms involved^61^. The hippocampus appears to prioritize novelty detection via transient dopamine bursts, which enhance theta-gamma PAC during exploratory navigation (Supplementary Note 5; Supplementary Fig. 16). In the context of our paradigm, which relies on well-established and predictable rewards, such a mechanism would be less engaged. Conversely, the AIC may integrate predictable reward information through more sustained dopaminergic modulation^61,63,64^, thereby supporting the stable anticipatory theta-gamma coordination observed in this study.

Taken together, our population-level findings reveal a temporally ordered and correlated interplay between representational pre-activation and oscillatory coordination within human AIC for reward expectancy. However, it should be noted that the observed correlation and temporal sequence between PAC and RBP pre-activation exhibited heterogeneity across individual recording contacts. Moreover, the extremely limited number of unrewarded trials prevented a statistically meaningful comparison between reward and no-reward conditions. To mitigate this limitation, we employed trial-matched EDZ periods as a control condition for ERZ, effectively accounting for confounding factors such as visual stimulus processing, spatial anticipation, and action preparation. This approach enabled maximal control for general task-related and motor-preparatory activity, thereby isolating neural signals specific to reward expectancy. Future studies incorporating more extensive electrode coverage and causal manipulations will be essential to clarify the consistency and directionality of this relationship.

### Phase precession effect in macroscale brain signals

The phase precession phenomenon, a form of precise phase-tuned temporal coding, was initially established in microscopic neuronal signaling. Specifically, single-units spike locking to progressively advanced phases across successive theta cycles^65,66^. This effect has since been documented in various species, including rodents, non-human primates, and humans^38,41,67,68^. Although it is most frequently reported in the medial temporal regions, phase precession extends to higher-order brain areas, such as the prefrontal and anterior cingulate cortices, during goal-directed behavior^16,36,68^. It has been posited as a critical mechanism for cognitive processing, whereby behavioral sequences are compressed into accelerated neural representations, enabling the prediction of upcoming stimuli^36,41,65,66,68,69^. Inspiringly, the strength of phase precession correlates with successful memory encoding and retrieval^68^.

Notably, recent perspectives reframe phase precession as an emergent phenomenon of network-level coordination, not merely as a single-neuron property^70,71^. Elucidating the mechanisms and functional roles of macroscopic phase precession, particularly in large-scale cortical networks, is essential for understanding how neural dynamics govern cognition and behavior. High gamma activity (HGA, > ~80 Hz), a robust proxy for local population neuronal firing^72,73^, provides a macroscopic agent for such phenomena. Nevertheless, direct evidence for theta phase precession effect in HGA remains sparse. A notable advance comes from Zheng et al., who identified cross-regional theta-gamma interactions during facial perception in a contextual modulation task^34^. Their work revealed a macroscopic phase precession effect, extending this phenomenon beyond single-unit spiking to population-level HGA and broadening its relevance to non-spatial cognitive states (e.g., transitions between task stages such as context maintenance and face perception). In the present study, we identified an analogous, yet functionally specific, temporally structured HGA in human AIC. During reward expectancy, peak gamma activity derived from PAC analysis was found to be systematically advanced towards earlier theta phases as rewards approached across trials, a phenomenon termed PPLE. This finding establishes PPLE as a multiscale neural principle spanning spatial domains (from single-unit spikes to HGA) and temporal scales (from hundreds of millisecond-long oscillatory cycles to tens of second-long behavioral epochs). Intriguingly, a recent neuromodulation study has demonstrated that the preferred phase for motor excitability progressively advances across successive tACS cycles^71^, providing independent empirical support for the observation of phase precession effect at macroscopic scales. These observations provide a unified framework for exploring how theta oscillations organizes neural activity across resolutions via phase precession effects. They shed light on the implications for cognitive processes and neuro-modulatory interventions.

Crucially, the PPLE identified in the AIC demonstrated clear functional specificity. It was selectively associated with reward expectancy, rather than reward outcome or a particular stimulus/location. This further supports the specific involvement of AIC in reward expectancy. In contrast, no PPLE was observed in the hippocampus before reward onset, which is consistent with previous rodent studies indicating that hippocampal phase precession remains uniformly distributed and independent of reward expectancy^41,42^. These dissociable profile highlight that the PPLE is not a general property of theta-gamma coordination, but rather a specific timing mechanism engaged within the AIC to support reward-directed behavior.

Nevertheless, the observation that the PPLE was present in 9 out of 25 contacts implies the spatial specificity corresponding to the AIC’s known fine-grained functional architecture^74^. Further investigation employing higher-density electrode coverage is warranted to map the precise topography of PPLE and clarify its relationship with functional subdomains of the AIC. Additionally, while our work bridges scales by demonstrating macroscopic PPLE in population‑level HGA, the exact relationship between this signature and classic microscale phase precession remains unclear. Future studies using simultaneous micro- and macroscopic recordings are necessary.

### Reward expectancy promote behavioral performance

Reward expectancy plays a crucial role in optimizing adaptive behavior by allocating cognitive resources and priming motor systems^4,6,10,42^, thereby enabling accelerated and precise responses to reward-related cues^2^. This process relies on the establishment of context-reward association via associative learning^2^. When encountering conditioned contexts, preactivated RBPs mediate the integration of bottom-up spatial cues with top-down reward-related memories, generating subsequent reward expectancy^3^. This process prioritizes attentional and motivational resources towards expected rewards, leveraging their salience within contextual frameworks to prime preparatory motor and cognitive states^3,4,53^. Consequently, participants exhibit enhanced behavioral performance, including reduced response latency and improved accuracy, through pre-configured motor readiness^75^. Expected reward outcomes further reinforce existing context-reward associations via feedback mechanisms^2,57^.

In this study, the observed RBP pre-activations and theta-gamma PAC during the reward expectancy stage constitute a neural signature of the formation of context-reward associations. These dynamics reflect the compression of real-world events into neural timescales, facilitating reward anticipation. We observed that the strength of PPLE was correlated with the magnitude of trial-by-trial behavioral improvement. This suggests that PPLE reflects a precise phase-tuned timing mechanism engaged during reward expectancy. Future studies with larger sample size and greater statistical power are needed to determine whether the PPLE specifically tracks reward-specific processes or is related to general practice effects. Nevertheless, the relationship between PPLE strength and the progressive optimization of responses towards upcoming rewards highlights a potential role for phase-tuned oscillatory dynamics in optimizing behavioral performances^1,2,4^. This raises the possibility that neuromodulation targeting such phase-coordinated activity could support cognitive and behavioral flexibility.

Methodologically, we implemented several controls to improve interpretability. To minimize the effects of fatigue, all participants completed experiments of comparable duration after adequate rest. A pre-experiment practice session ensured task proficiency and reduced learning-related confounds within the main task. Additionally, fixed reward parameters eliminated the confounding influence of reward types and magnitudes. These strategies help isolate the neural and behavioral correlates of reward expectancy. Future research should employ multidimensional reward paradigms that systematically vary reward types (e.g., psychological versus material), intensity, salience, and predictability. These designs will allow for a more precise characterization of the spatiotemporal dynamics underlying reward expectancy, including representational (e.g., RBP) and oscillatory (e.g., PAC) mechanisms. This refined understanding could inform the development of closed-loop neuromodulation protocols that selectively enhance context-reward associations to optimize adaptive behavior in real-world scenarios.

### Conclusions

Our findings suggest that the AIC serves an important role in linking predictive processes with adaptive behavior, potentially through phase-tuned oscillatory modulation. This temporal recalibration appears to begin with RBP pre-activation, which is followed by enhanced theta-gamma PAC. Notably, this coupling exhibits a macroscopic PPLE, characterized by the progressive advancement of peak gamma activity toward earlier theta phases across successive navigation rounds. This dynamic phase refinement of phase coding is associated with improved reward-collection performance, underscoring how precise temporal orchestration within the AIC optimizes reward expectancy.

## Methods

### Participants

Thirteen medically refractory epilepsy patients with depth electrodes implanted for pre-surgical evaluation (six males; average age = 25.25 ± 8.87 years [mean ± SD]; Supplementary Table 1) were recruited from the Hôpital de la Salpêtrière (n = 12) and the Second Affiliated Hospital of Zhejiang University (n = 1). The study was approved by the Medical Ethics Committee at the hospital sites (INSERMC11-16 and StudyNo.2020-910, respectively), and written informed consents were obtained from all participants. All ethical regulations relevant to human research participants were followed.

### Experimental design and procedure

We built a virtual T-maze using Panda3D with PandaEPL extensions, allowing for free navigation. As illustrated in Fig. 1b, the T-maze consists of a central corridor (32 virtual units [VUs] in length and 4 VUs in width), two lateral arms (24 VUs apart), a starting zone, a decision zone, and three reward zones. Participants initiated each navigation round in the designed starting zone, advancing along the central corridor that contained a guaranteed-reward zone (0.15€ coin). Upon reaching the designated decision zone, a wall-mounted directional cue (circle for left-turn, and triangle for right-turn) indicated the correct lateral-arm path leading to an additional reward (Fig. 1c and d). If the incorrect lateral arm was chosen, no reward was available on that route. Visible coins required a prompt “Enter” key press for collection before automatic disappearance upon reward-zone exit. Following reward collection, participants returned to the starting zone along the lateral arm to begin subsequent navigation round. Participants were encouraged to collect enough gold coins (10€) as soon as possible. To avoid being disoriented, participants were asked to memorize as many pictures hanging on the wall as possible to incentivize them to pay attention to the task and their ongoing position. Furthermore, different wall-mounted paintings also helped participants to allocate themselves inside the maze to avoid being disoriented (Fig. 1d). We tested their knowledge about the location of pictures at the end of the experiment (data not reported here). Before the experiment, participants completed a practice session in a similar virtual maze. This session was designed to fully familiarize participants with both maze navigation (e.g., walking straight and turning) and the experimental procedure (e.g., picking up the reward upon its display), thereby minimizing potential practice effects in the main task. On average, participants completed 36.08 ± 2.96 (mean ± SD) navigation rounds, with each experimental session lasting over one hour.

Participants were positioned comfortably on their beds, with a laptop placed on a table directly in front of them. Navigation was controlled via arrow buttons on the keyboard at 6 VUs/second velocity (40 VUs/second^2^ acceleration). A mandatory 2-second pause when the reward was picked up or when the decision zone was entered. Directional cues (signs indexing left- or right-turn) were randomly generated for each navigation round but counter-balanced across navigation rounds. During the experiment, the testing laptop sent triggers to the iEEG recording system to synchronize behavioral and iEEG data for offline analyses.

### Behavioral data collection and process

Navigation trajectories were continuously logged during the experiment. We focused on three events for further behavioral and iEEG data analyses: entering the reward zone (ERZ; experimental condition), picking up the reward (PR; control condition), and entering the decision zone (EDZ; control condition). When participants entered a reward zone, a gold coin immediately appeared on the monitor, which was termed as the ERZ (Fig. 1d). The event corresponding to PR was defined as the moment when participants pressed the “Enter” button to collect rewards. Additionally, upon EDZ, a directional cue was displayed on the wall. To minimize the influence of learning biases, we specifically focused on the reward zone in the central corridor, where participants’ expectations for receiving a reward were consistently fulfilled (i.e., the reward would certainly appear). Another advantage is ensuring the balance among the trial numbers under the three conditions included in the analysis. Nevertheless, ERZ events occurred on the lateral roads were also extracted to identify the reward-specific brain patterns (RBPs).

The response time (RT) for picking up a reward was calculated as a behavioral parameter for each ERZ event in the central corridor, referring to the time interval between the ERZ event and the corresponding PR event. To examine the improvement in RT across navigation rounds, we computed the z-scored RT for each navigation round in each participant, excluding navigation rounds with z-scored RT > 3 for further data analysis^76^. We then performed a linear regression analysis for z-scored RT using the number of navigation rounds. We tested the significance of RT decline pattern by performing a surrogate analysis on each participant. Within each permutation, we randomly shuffled the round labels, computed the linear regression again, and obtained a surrogate coefficient. The permutation procedure was repeated 500 times, generating 500 surrogate coefficients. We then ranked the empirical coefficient within the distribution of surrogate coefficients for each participant. If the empirical correlation values were negative and below the 95^th^ percentile of the null distribution, these values were considered significant, signifying behavioral improvement.

### Electrode reconstruction

The electrode implantation schemes were planned purely for clinical pursues. The Ad-Tech electrodes used at the Hôpital de la Salpêtrière were equipped with 4-12 contacts, each with a diameter of 1 mm, a length of 2.41 mm and a contact spacing of 5 mm^77^. Meanwhile, the HKHS electrodes employed at the Second Affiliated Hospital of Zhejiang University consisted of 8-16 contacts, with a diameter of 0.8 mm, a length of 2 mm, and a spacing of 1.5 mm^78^.

We selected contacts located in the AIC for further data analyses and contacts in the hippocampus for control analyses, which are known to be closely associated with reward processing and spatial memory^13,25,26^. The exact location of each contact was defined by visual inspection in local space of each participant using FSLeyes (https://fsl.fmrib.ox.ac.uk/fsl/fslwiki/FSLeyes).

After excluding contacts in the seizure onset zones and those contaminated by epileptic spikes, we obtained 25 AIC contacts (18 in the right hemisphere) from 9 participants and 19 hippocampal contacts (9 in the right hemisphere) from 7 participants (Fig. 1a and Supplementary Table 1). Three participants (#8, #14, and #15) had both AIC and hippocampal contacts.

### IEEG data acquisition and preprocessing

IEEG data were acquired at sampling rate of ≥1k Hz using an Ad-Tech system (Hôpital de la Salpêtrière) and a Nihon-Kohden recording system (Second Affiliated Hospital of Zhejiang University; Supplementary Table 1). During offline data analysis, the data were band-pass filtered from 0.01 to 200 Hz, notch filtered at 50 Hz and its harmonics, and then down-sampled to 1k Hz. The filtered data were then re-referenced to the average activity of all depth electrodes outside the seizure onset zones^45^. We extracted the iEEG activity from 3 seconds before to 3 seconds after each event (i.e., ERZ, PR, and EDZ). Relatively long segments were chosen to eliminate edge effects associated with spectrum-temporal decomposition. EEG trials were excluded for the following reasons: 1) clinical interruptions (e.g., medication administration; one ERZ epoch from participant #8), 2) reward omissions (i.e., exiting reward zones without collecting; one PR epoch from participant #15), and 3) incorrect responses at the decision zones (i.e., erroneous arm selections; four EDZ epochs from participant #16 and one from #17). Additionally, following visual inspection, EEG trials contaminated by artifacts (e.g., epileptic activity) were excluded. The numbers of excluded trials were 4.00 ± 2.86, 3.62 ± 2.69, and 3.46 ± 2.14 (mean ± SD) for ERZ, PR, and EDZ events, respectively (Fig. 1e and Supplementary Table 1).

Time-frequency decomposition was performed on each EEG trial using Morlet wavelet transformation (6 cycles), and power values were extracted. Only data from 2 seconds before to 2 seconds after each event was kept for further analyses. We then z-transformed the EEG power data within each frequency and each recording contact across all trials. Further data analyses were based on normalized data. Data preprocessing was performed using EEGLAB toolbox and custom MATLAB code^79^.

### Identification of RBPs and their pre-activation

Reward expectancy indicates that the brain starts to process reward-relevant information before the onset of rewards, which triggers the activation of reward-relevant brain activity. Thus, we hypothesized that brain patterns representing rewards would be preactivated prior to actual reward delivery. To test this hypothesis, we investigated RBP pre-activation in AIC and hippocampal contacts before reward onset using representational similarity analysis (RSA)^45–48^.

To identify RBPs, we analyzed post-ERZ periods from three separate reward zones for each recording contact (Fig. 1b). Each post-ERZ period is from the display of reward (i.e., the onset of ERZ events) to 2 seconds afterward. First, we segmented each post-ERZ period into 200-ms windows with 25-ms overlap, yielding 73 time windows. Then we computed the averaged iEEG power across time within each window and in each frequency (1-30 Hz in 1-Hz steps, 35-150 Hz in 5-Hz steps; Fig. 2a). It resulted in a 54 (frequencies) × 73 (time windows) matrix per post-ERZ period for each contact. For each post-ERZ pair (trial m vs. trial n), neural similarity was quantified as Spearman’s correlation between their EEG power patterns across 54 frequencies within each corresponding time window. It generated a 73 × 73 time-resolved similarity matrix for each post-ERZ pair. The correlation coefficients were then Fisher-z-transformed and averaged across the time windows of trial n, yielding a 1 × 73 similarity vector for each post-ERZ pair (RSA_postERZ/postERZ_; Fig. 2b). To isolate RBPs from general neural activity, we compared brain patterns between post-ERZ and post-EDZ periods. Using the same procedure, a similarity vector for each post-ERZ/post-EDZ pair (RSA_postERZ/postEDZ_) was generated. Crucially, pairs from the same navigation round or same spatial location were excluded to control for potential confounds due to temporal proximity and location-specific effects. To assess the statistical difference between RSA_postERZ/postERZ_ and RSA_postERZ/postEDZ_, we conducted multi-level linear regression analysis for each time bin using fitlme with the following formula in MATLAB:

$${{\rm{RSA}}} \, {{\rm{value}}} \sim {{\rm{Cue}}} \, + (1|{{\rm{Participant}}}) \, + (1|{{\rm{Contact}}})$$

in which the single‑trial RSA value was the dependent variable, and the cue condition (postERZ/postERZ vs. postERZ/postEDZ) was the fixed effect. Random effects included by‑participant intercepts and by‑contact intercepts. Significant bins (p < 0.05) adjacent to each other were grouped into one cluster, with corresponding statistical values calculated by summing t-values of all bins within the cluster. Cluster-level significance was determined through 500 permutation tests, in which the condition labels (post-ERZ vs. post-EDZ) were randomly shuffled^50^. For each permutation, we repeated the same procedure as the empirical data and identified the cluster with the maximum statistical value as the surrogate cluster. If no cluster was identified, a value of zero was assigned. Empirical clusters exceeding the 95^th^ percentile of all surrogate clusters were deemed significant. Time windows showing RSA_postERZ/postERZ_ significantly higher than RSA_postERZ/postEDZ_ were considered to carry RBPs.

We subsequently examined whether RBPs were preactivated before reward onset. We first extracted the pre-ERZ period spanning from 2 seconds before each ERZ event to its onset. Using the same preprocessing pipeline applied to post-ERZ periods, we converted each pre-ERZ period into a 54 (frequencies)×73 (time windows) power matrix. To control for location-specific confounds, we compared brain patterns in each pre-ERZ time window (on the central corridor) with RBPs derived from post-ERZ periods (on the lateral roads) using Spearman’s correlations. This generated a 1 × 73 similarity vector for each pre-ERZ period and each recording contact (RSA_preERZ/RBP_; Fig. 2c). As a control analysis, the same procedure was applied to pre-EDZ periods (i.e., from 2 seconds before EDZ event), yielding RSA_preEDZ/RBP_. To avoid temporal autocorrelations, similarity values (RSA_preERZ/RBP_ and RSA_preEDZ/RBP_) from the same navigation round were excluded for further analysis. We then performed a statistical comparison between RSA_preERZ/RBP_ and RSA_preEDZ/RBP_ for each time window, using multi-level linear regression that considered participant- and contact-level variances. Time bins with p < 0.05 were deemed significant and grouped into adjacent clusters. The statistical value of each cluster was calculated by summing the t-values from the regression analysis. Cluster significance was assessed through permutation analysis^50^, in which we randomly re-assigned the condition labels (pre-ERZ vs. pre-EDZ). For each permutation, we repeated the same procedure as with the empirical data, identifying the cluster with the highest statistical value as a surrogate value. The permutation was performed 500 times, generating 500 surrogate values. The empirical cluster was considered significant if its statistical value exceeded the 95^th^ percentile of the surrogate distribution. Time windows showing significantly elevated RSA_preERZ/RBP_ relative to baseline (RSA_preEDZ/RBP_) indicated RBP pre-activation.

### Detection of phase-amplitude coupling (PAC) during reward expectancy

To estimate the neural dynamics of the AIC during reward expectancy, we computed the PAC strength before entering the reward zone (i.e., pre-ERZ periods) using modulation index (MI). The MI is based on the Kullbeck-Leiber distance, which is a measure of the divergence between the uniform distribution and the probability distribution of high-frequency amplitudes^49,80^. For each pre-ERZ period, we applied finite impulse response (FIR) filtering to iEEG data, yielding dynamics for each low-frequency band (1-12 Hz in 1-Hz steps, 12 samples of frequency_low_; Supplementary Note 6; Supplementary Fig. 17) and each high-frequency band (30-150 Hz in 5-Hz steps, 25 samples of frequency_high_). The low-frequency signals were filtered with a 1-Hz bandwidth and the high-frequency signals were filtered with a 24-Hz bandwidth, according to the Heisenberg-Gabor limit^81,82^. Each filtered low-frequency band was visually inspected to confirm its sinusoidal nature. Instantaneous phases of the low-frequency signals and the amplitudes of the high-frequency signals were extracted using the Hilbert transformation. Phases were then binned into eighteen 20° intervals (-180° to 180°), and MI values were computed for each low-frequency phase and high-frequency amplitude pair. The obtained MI values were further normalized against 200 surrogate datasets generated by randomly shuffling the trial numbers of high-frequency signals. We applied the same analysis to post-ERZ periods.

To explore the differences in PAC strength between the pre- and post-ERZ periods, we conducted a multi-level linear regression analysis on z-scored MI values by considering variances at both participant and contact levels for each frequency_low_-frequency_high_ bin. Bins with a p-value smaller than 0.05 were considered significant. Adjacent significant bins were clustered together^51^. For each cluster, a cluster-level statistic was computed by summing the t-values obtained from the multi-level linear regression analysis. To correct for multiple comparisons, we implemented a cluster-based permutation analysis^50^. Within each permutation test, we randomly shuffled condition labels (i.e., pre- or post-ERZ period) and performed the same multi-level linear regression analysis. This generated a set of surrogate MI clusters. The MI cluster with the largest statistical value was selected. This procedure was repeated 500 times, generating 500 surrogate MI clusters. Empirical clusters exceeding the 95^th^ percentile of the surrogate MI distribution were deemed significant (p < 0.05). Enhanced PAC during reward expectancy was indicated by frequency_low_-frequency_high_ clusters showing significantly greater MI values during pre-ERZ versus post-ERZ periods.

### Analysis of phase shifts in PAC temporal patterns

To characterize trial-wise PAC dynamics, we extracted the preferred PAC phase within significant frequency_low_-frequency_high_ clusters during pre-ERZ periods at the central road. For each trial, the low-frequency phases were divided into 18 bins of 20° each ([-180°, -160°], [-160°, -140°], …, [160°, 180°]), and the high-frequency amplitudes were averaged across frequencies within the PAC cluster for each phase bin^83^. The highest mean high-frequency amplitudes were considered to be peak high-frequency activity, and the corresponding phase bins were defined as the preferred PAC phase.

Subsequently, we investigated the dynamics of the preferred PAC phases across navigation rounds. For each recording contact, we performed a circular-linear correlation analysis^36^, treating the number of navigation rounds as the linear variable and the preferred phases during pre-ERZ periods as the circular variable. A negative correlation coefficient indicates progressive advancement of preferred phases toward reward approach, whereas a positive correlation coefficient reflects phase retardation. Statistical significance was assessed through permutation tests. Within each permutation, we randomly shuffled round labels and performed the same circular-linear correlation analysis. It generated one surrogate value. This procedure was repeated 500 times to generate 500 surrogate values^50^. Empirical coefficients exceeding the 95^th^ percentile or falling below the 5^th^ percentile of the surrogate distribution (p < 0.05) were considered significant, indicating phase shifts in PAC dynamics across navigation rounds.

To exclude systematic temporal shifts in peak high-frequency activity as a confounding factor in phase-tuned modulation of PAC effects, we performed a linear regression analysis of the timepoints corresponding to peak high-frequency activity against the number of navigation rounds. Significance level for coefficient values were estimated by surrogate analysis. Within each permutation, we randomly shuffled round labels and then recomputed the linear correlation coefficient^50^. This was repeated 500 times, yielding 500 linear correlation coefficients. Empirical coefficients exceeding the 95^th^ percentile or falling below the 5^th^ percentile of the null distribution were considered significant.

### Statistics and Reproducibility

#### Software

All statistical analyses were performed using either SPSS version 26.0 (IBM Corp., Armonk, NY, USA) or MATLAB 2022b (MathWorks, Natick, USA) and illustrated using GraphPad Prism version 9.5 (GraphPad Software, La Jolla California USA).

#### Sample size and replicates

For electrophysiological data, the unit of analysis was either individual recording contacts (AIC: n = 25 contacts from 9 participants; hippocampus: n = 19 contacts from 7 participants) or participants, as specified in each figure legend. Trial‑wise behavioral improvement was analyzed across navigation rounds; the number of trials per participant is provided in Supplementary Table 1. No statistical methods were used to predetermine sample sizes, but our sample sizes are comparable to those reported in previous iEEG studies^34,45^.

#### Parametric and non‑parametric tests

Normality of data distribution was assessed using the Shapiro-Wilk test. For data that met normality assumptions, parametric tests were used: paired or independent t‑tests, Pearson’s correlation, and one-way ANOVA. For data that did not meet normality assumptions, non‑parametric alternatives were applied: Wilcoxon matched‑pairs signed‑rank test, Mann–Whitney U test, Spearman’s correlation, or Friedman test with post‑hoc test. Pearson’s r or Spearman’s r are reported as effect sizes for correlations. Where applicable, 95% CIs for correlation coefficients are provided. Repeated‑measures data (e.g., RSA values, and PAC strength) were analyzed using linear mixed‑effects models with participant and contact (nested within participant) as random intercepts to account for within‑participant and within‑contact dependencies across trials.

#### Cluster‑based permutation tests

The non‑parametric cluster‑based permutation test was used to assess statistical significance for RSA values, PAC strength, PPLE strength, and behavioral improvement^50^. A threshold of α = 0.05 was applied, with 500 permutations. Cluster p‑values were calculated as the proportion of permuted clusters exceeding the observed cluster statistic.

#### Reproducibility

All experiments were performed in accordance with the study protocol approved by the institutional review board. The results presented are based on the full dataset, with no exclusions other than those explicitly stated: outlier removal based on ±3 SD from the mean (for RT analysis) and rejection of contaminated EEG epochs (as described in the main Methods).

### Reporting summary

Further information on research design is available in the Nature Portfolio Reporting Summary linked to this article.

## Supplementary information


Supplementary Material
Reporting Summary
Transparent Peer Review file


## Data Availability

The raw iEEG data are confidential medical records and cannot be publicly deposited due to ethical and privacy restrictions. All numerical data used to generate the data shown are available at the Figshare repository^84^ https://doi.org/10.6084/m9.figshare.30413173. Additional data that support the findings of this study are available from the corresponding author upon reasonable request.

## Code availability

The custom-written Matlab and Python code supporting the findings of this study will be made available at the Figshare repository^84^ https://doi.org/10.6084/m9.figshare.30413173.
