# Supplementary material for: Phase-tuned modulation during reward expectancy in human anterior insular cortex
Source: Commun Biol. 2026 Jun 16;9:1148. doi: 10.1038/s42003-026-10427-1 (PMC13521940; doi:10.1038/s42003-026-10427-1)
Supplement: Supplementary file 1 [file 42003_2026_10427_MOESM1_ESM.pdf]

Supplementary Information for

Phase-tuned modulation during reward expectancy in human anterior insular cortex

Linglin Yang[1,2], Katia Lehongre[3], Xinfeng Yu[4], Hongyi Ye[2], Vincent Navarro [5,6,7], Sen Cheng[8], Nikolai Axmacher[9,10], Shuang Wang[2, *], Hui Zhang[9, *]

* These authors jointly supervised this work.

Email: Hui.Zhang-c5u@ruhr-uni-bochum.de (Hui Zhang); wangs77@zju.edu.cn (Shuang Wang)

This PDF file includes:

- **Supplementary Note 1. Additional control analyses using representational similarity analysis (RSA)**
- **Supplementary Note 2. Control analysis for gamma-amplitude confounds in phase-amplitude coupling (PAC)**
- **Supplementary Note 3. Temporal relationship between RBP pre-activation and peak theta-gamma PAC across trials, contacts, and participants**
- **Supplementary Note 4. Behavioral improvement correlates with RBP pre-activation but not with PAC strength**
- **Supplementary Note 5. Theta activity and PAC in the hippocampus during virtual spatial navigation**
- **Supplementary Note 6. Spectral dynamics of theta and alpha oscillations in the AIC and hippocampus**
- **Supplementary Table 1. Demographic information. Related to Figure 1.**
- **Supplementary Table 2. Time difference between the initial pre-activation of reward-specific brain patterns and the peak gamma activity in individual contacts. Related to Figure 3.**
- **Supplementary Figure 1. Reward-specific brain pattern (RBPs) in the anterior insular cortex. Related to Figure 2.**

- **Supplementary Figure 2. Differential RBP pre-activation during reward versus decision context at the contact level. Related to Figure 2.**
- **Supplementary Figure 3. Phase-amplitude coupling (PAC) difference between before and after each experimental event for AIC contacts, respectively. Related to Figure 3.**
- **Supplementary Figure 4. Phase-amplitude coupling (PAC) difference between before and after experimental events in hippocampal contacts. Related to Figure 3.**
- **Supplementary Figure 5. Theta–gamma phase-amplitude coupling (PAC) exhibits substantial independence from gamma-band amplitude fluctuations. Related to Figure 3.**
- **Supplementary Figure 6. Theta–gamma PAC exhibits positive correlation with RBP pre-activation level. Relate to Figure 3.**
- **Supplementary Figure 7. Temporal sequence of RBP pre-activation and PAC effect at the trial level. Related to Figure 3.**
- **Supplementary Figure 8. Temporal sequence of RBP pre-activation and PAC effect at the contact and participant level. Related to Figure 3.**
- **Supplementary Figure 9. PPLE before entering reward zones in AIC and hippocampal contacts. Related to Figure 4.**
- **Supplementary Figure 10. Relationship between the time of peak gamma activity and the number of navigation rounds in AIC contacts.**
- **Supplementary Figure 11. PPLE before entering decision zones in AIC and hippocampal contacts. Related to Figure 4.**
- **Supplementary Figure 12. PPLE before picking up rewards in AIC and hippocampal contacts. Related to Figure 4.**
- **Supplementary Figure 13. Relationship between PPLE strength and RBP pre-activation level or PAC strength. Related to Figure 4.**
- **Supplementary Figure 14. Response time acceleration across navigation rounds in participants with AIC contacts. Related to Figure 1 and 5.**
- **Supplementary Figure 15. Relationship between behavioral performance and identified neural signatures. Relation to Figure 5.**
- **Supplementary Figure 16. Enhanced theta activity and altered phase-amplitude coupling (PAC) in the hippocampus. Relate to Figure 3.**
- **Supplementary Figure 17. Spectral characterization of low-frequency oscillations in the AIC and hippocampus during virtual navigation.**
- **Supplementary References**

Supplementary Note 1. Additional control analyses using representational similarity analysis (RSA)

Furthermore, to rule out the possibility that reward-specific brain patterns (RBPs) identified in the anterior insular cortex (AIC) were confounded by motor preparation related to picking up the reward (PR), we conducted additional control analyses using the RSA approach described in main text. Specifically, we compared neural pattern similarity for reward-viewing pairs ($RSA_{postERZ/postERZ}$) against that for reward-viewing versus reward-picking pairs ($RSA_{postERZ/prePR}$). Neural activity within 0.975-1.3 seconds following reward onset showed significantly higher $RSA_{postERZ/postERZ}$ than $RSA_{postERZ/prePR}$ ($p_{corrected}$=0.023; Supplementary Figure 1). The observation aligns with the contrast between $RSA_{postERZ/postERZ}$ and $RSA_{postERZ/postEDZ}$ reported earlier, further supporting that the identified RBPs reflect reward-specific representation, rather than motor preparation for the impending button press.

Supplementary Note 2. Control analysis for gamma-amplitude confounds in phase-amplitude coupling (PAC)

To rule out the possibility that the observed enhancement in theta-gamma PAC was confounded by fluctuations in gamma-band amplitude, we conducted an additional control analysis. For each recording contact, we assessed the correlation between PAC strength and averaged gamma amplitude (75-110 Hz) during the period before entering the reward zones (ERZ) across trials. In 23 out of 25 contacts, gamma amplitude and PAC strength were not significantly correlated ($ps \geqslant 0.15$, Pearson's correlation; Supplementary Figure 5). This finding indicates that the reported theta-gamma PAC effect is largely independent of variations in gamma amplitude, supporting the interpretation that it reflects genuine oscillatory coordination rather than an amplitude-driven artifact.

Supplementary Note 3. Temporal relationship between RBP pre-activation and peak theta-gamma PAC across trials, contacts, and participants

In individual contacts, the peak PAC effect was significantly delayed relative to RBP pre-activation across trials in 24 out of 25 contacts (ps<0.001), with no significant difference in the remaining contact (p=0.697; Supplementary Figure 7). We again investigated the time lag within each contact and found high dispersed distributions of the time lag in all contacts (CVs≥62%; Supplementary Table 2). We then averaged the timing of both events across trials for each contact and compared them at the contact level (Supplementary Table 2 and Supplementary Figure 8a). Consistent with the above results, the peak PAC effect occurred significantly later than RBP pre-activation across the 25 contacts (-1.00±0.004 seconds vs. -1.72±0.004 seconds, p<0.001; Supplementary Figure 8b), with delays ranging from 0.04 to 0.92 seconds (CV=23%), suggesting a moderate dispersed distribution of the time lag across contacts. To preclude potential bias introduced by uneven contact numbers across participants, we performed the analysis at the participant level by averaging across contacts within each participant. The peak PAC effect (-0.97±0.08 seconds [mean±SD]) again occurred significantly later than the initial RBP pre-activation (-1.70±0.09 seconds; p=0.0039; Supplementary Figure 8c). The time lags at the participant level ranged from 0.45 to 0.90 seconds (CV=18.8%), further indicating the absence of a fixed temporal delay between the onset of RBP pre-activation and the peak of theta-gamma oscillatory coordination. These findings demonstrate a temporal sequence in which RBP pre-activation consistently precedes before the peak of theta-gamma oscillatory coordination during the pre-ERZ period. Furthermore, this temporal sequence pattern was robust across trials, contacts, and participants. However, the interval between these two events is highly variable, suggesting that the precise timing of their interaction is not fixed, but may instead be modulated by ongoing cognitive demands.

Supplementary Note 4. Behavioral improvement correlates with RBP pre-activation but not with PAC strength

We examined how behavioral improvement related to RBP pre-activation level and PAC strength. The result showed that the magnitude of behavioral improvement showed a negative correlation with the average level of RBP

pre-activation across participants (r=-0.83, p=0.006; Pearson's correlation), but no significant correlation with average PAC strength (r=-0.22, p=0.57; Supplementary Figure 15a and b). These results do not support a simple linear cascade in which representational pre-activation and cross-frequency coupling directly drive reward-directed behavioral improvement. Notably, the absence of a significant correlation with PAC should be interpreted with caution, as PAC estimates are more variable than power-based measures[1], potentially requiring greater statistical power to detect behavioral links.

Supplementary Note 5. Theta activity and PAC in the hippocampus during virtual spatial navigation

Previous research has demonstrated that rodent hippocampus exhibits prominent theta activity (5-10 Hz) during locomotion, with both frequency and power increasing as the running speed increases[2]. In humans, however, theta oscillations during virtual navigation are more heterogeneous, spanning a broader range (2-9 Hz)[3-5]. While human theta power is indeed sensitive to movement (e.g., higher during locomotion than stillness)[5-7], it is also influenced by attention and memory demands[5,8]. For instance, theta power increases significantly during decision-making, and correlates with decision speed and choice accuracy[9,10]. Notably, the dominant theta frequency can shift depending on task demands[11], with higher-frequency theta activity potentially supporting tasks requiring concentrated spatial attention[11]. This frequency-specific modulation suggests that distinct cognitive processes may be supported by different theta sub-bands.

In the present study, we investigated low-frequency power dynamics by computing z-scored power (1-12 Hz in 0.5-Hz steps; see Methods) for time windows surrounding ERZ and EDZ events. To explore differences in low-frequency power between pre- and post-event (ERZ or EDZ) periods, we employed a multi-level linear regression analysis on the z-scored power values, accounting for variances at both the participant and contact levels for each time-frequency bin. Bins with a p-value < 0.05 were considered significant, and adjacent significant bins were clustered together[12]. For a given cluster, a summed t-value across all bins was assigned as the statistical value of that cluster. To compute the significance level of each cluster, we implemented a cluster-based permutation analysis[13]. Within each permutation test, we randomly shuffled condition labels (i.e., pre- or post-event period) and repeated the multi-level linear regression analysis. This generated a set of surrogate clusters, from which the cluster

with the maximum statistical value was retained. This procedure was repeated 500 times, generating 500 surrogate clusters. Empirical clusters exceeding the 95th percentile of the surrogate distribution were considered significant ($p<0.05$). For ERZ event, higher-frequency theta activity (9-12 Hz) was enhanced during pre-ERZ (i.e., moving) relative to post-ERZ (i.e., stillness) periods (-1.662 to -1.078 seconds; p=0.009; Supplementary Figure 16a), reflecting locomotion-related theta activity[2,6,7]. A parallel analysis of EDZ events similarly showed enhanced higher-frequency theta (6-12 Hz) during movement (pre-EDZ periods; -1.592 to -1.136 seconds; p=0.001; Supplementary Figure 16b), further confirming locomotion-related theta modulation[2,6,7]. Additionally, lower-frequency theta activity (3-6.5 Hz) increased after EDZ events (1.284 to 1.902 seconds; p=0.007), suggesting decision-related neural correlates[9]. These findings reveal functionally distinct theta rhythms in the human hippocampus during virtual navigation, aligning with prior work[5-7,9,11]. Specifically, higher-frequency theta activity associated with locomotion and lower-frequency theta activity linked to decision process.

PAC between low-frequency oscillations (LFOs) and gamma activity represents a fundamental organizing principle of hippocampal neural dynamics, enabling the integration of information across multiple temporal scales[14,15]. Accumulating evidence from rodents has established that theta-gamma PAC plays a critical role in spatial coding and path integration during navigation[14], and invasive intracranial electroencephalography (iEEG) studies in humans have similarly implicated hippocampal PAC in supporting working and long-term memory[16-18], with different PAC configurations supporting distinct cognitive processes. However, direct evidence for task-dependent modulation of hippocampal PAC during virtual spatial navigation in humans remains limited. This gap may be accounted for by two factors. First, spatial navigation involves a complex interplay of cognitive processes, including spatial mapping, decision-making, and salience processing, which makes it difficult to isolate PAC signatures associated with specific cognitive subcomponents[11]. Second, the human hippocampus exhibits considerable heterogeneity in its low-frequency spectral profile compared to the more stereotyped theta rhythm observed in rodents. Rather than displaying a narrow-band oscillation concentrated around a fixed frequency, human hippocampal LFOs show marked interindividual variability[4,11,19]. Some participants exhibit clear spectral peaks in the 7-9 Hz range during memory-guided navigation in virtual environments, while others show peaks in the 2-4 Hz range[11]. This broad-band and bimodal distribution may reflect the fact that human hippocampal oscillations are not exclusively dedicated to spatial processing, but also participate in internally generated activities, contextual construction, and cognitive control processes[20]. For each experimental condition, we computed average PAC

strength between two LFO bands of interest (2-4 Hz and 7-9 Hz) and four gamma sub-bands (30-55 Hz, 60-85 Hz, 90-115 Hz, and 120-145 Hz) which were reported in previous studies[4,6,11]. Our analyses revealed that hippocampal PAC hippocampal PAC varied dynamically across conditions (Supplementary Figure 16c). Prior to entering the reward zones, PAC between 7-9 Hz and 60-85 Hz was significantly decreased, suggesting a reorganization of information integration as participants approached the reward zone. Upon entering the reward zone, we observed enhanced PAC in two distinct frequency pairs—2-4 Hz with 30-55 Hz and 7-9 Hz with 120-145 Hz—pointing to a shift in oscillatory coupling configurations during reward acquisition and contextual updating. Immediately prior to reward collection, PAC between 7-9 Hz and 30-55 Hz was decreased, potentially reflecting reduced information integration or network reconfiguration prior to action execution. During the pre-EDZ period, PAC between 2-4 Hz and 120-145 Hz was reduced while coupling between 7-9 Hz and 30-55 Hz was enhanced, suggesting that different low-frequency rhythms differentially regulate distinct information channels during path selection and strategy evaluation. Upon entering the decision zone, PAC between 7-9 Hz and 30-55 Hz was decreased, possibly reflecting inhibition or resetting of task-related network activity following decision completion.

Collectively, these findings support the view that the hippocampus is continuously engaged in virtual spatial navigation, though its specific frequency composition undergoes dynamic reconfiguration according to changing cognitive demands. The dynamic reorganization of PAC likely constitutes a key mechanism through which the hippocampus integrates and allocates information across navigation, decision-making, and reward processing. Future studies that refine the dissection of cognitive subcomponents are essential to determining the specific functional contributions of each frequency pair. Moreover, higher-density sampling along the longitudinal axis of hippocampus may reveal regional specialization[12].

Supplementary Note 6. Spectral dynamics of theta and alpha oscillations in the AIC and hippocampus

Before performing further analyses on the oscillatory data, we characterized the spectral dynamics in the AIC and hippocampus across experimental events (e.g., ERZ, PR and EDZ; Supplementary Figure 17). This preliminary characterization aimed to establish a foundational profile of LFOs underlying task engagement.

To reliably identify oscillatory components, we applied the Multiple Oscillations Detection Algorithm (MODAL)[19]. For each trial and each contact, the power spectral density (PSD) was calculated between 1 and 50 Hz (in steps of 0.5 Hz) using a Morlet wavelet transformation with 6 cycles (Supplementary Figure 17a). Subsequently, a 1/f regression to the background spectrum was constructed in log-log space, and narrow-band oscillations exceeding the fitted line were identified. To evaluate the prevalence of oscillations at each frequency, the percentage of detected time was calculated for each contact and trial. These values were then averaged across trials, contacts, and participants for each experimental condition. In both the AIC and hippocampus, LFOs exhibited relatively constant and pronounced within event-centered time windows (Supplementary Figure 17a-e). In the AIC, theta oscillations (3.5-7.5 Hz) were the most prevalent, detected in nearly half of the time bins on average across participants (ERZ: 41.39±1.36% [mean±SD], PR: 42.84±1.62%, EDZ: 43.20±2.24%; Supplementary Figure 17d). Oscillations in the alpha (8-12 Hz; ERZ: 13.00±1.25%, PR: 12.83±1.24%, EDZ: 12.96±1.81%) and beta (12.5-30 Hz; ERZ: 13.44±1.55%, PR: 12.37±1.08%, EDZ: 13.45±1.73%) bands were less prevalent. The prevalence of theta oscillation significantly exceeded that of alpha and beta oscillations, respectively for each experimental condition (Friedman test: $ps \leq 3.0 \times 10^{-4}$; post-hoc Wilcoxon matched-pairs signed rank test: $ps \leq 3.9 \times 10^{-3}$ for theta vs. alpha and theta vs. beta). In contrast, alpha and beta oscillations showed no significant difference in prevalence (post-hoc Wilcoxon matched-pairs signed rank test: $ps \geq 0.50$). A similar spectral pattern was observed in the hippocampus during event-locked time windows (Supplementary Figure 17e). LFOs were predominantly observed in theta band (ERZ: 41.86±0.96%, PR: 42.33±1.06%, EDZ: 41.30±1.04%). In contrast, the prevalence of alpha-band (ERZ: 13.46±1.61%, PR: 12.78±1.67%, EDZ: 13.36±1.74%) and beta-band oscillations (ERZ: 13.02±2.43%, PR: 13.41±1.52%, EDZ: 12.68 ± 1.65%) were relatively lower. Theta oscillations demonstrated a significantly higher prevalence in comparison to alpha and beta oscillations across all experimental conditions (Friedman test: $ps \leq 1.2 \times 10^{-4}$; post-hoc Wilcoxon matched-pairs signed rank test: $ps \leq 0.016$ for theta vs. alpha and theta vs. beta). However, no significant difference in prevalence was observed between alpha and beta oscillations (post-hoc Wilcoxon matched-pairs signed rank test: $ps \geq 0.16$).

To further validate these observations, the Fitting Oscillations & One Over F (FOOOF) algorithm was employed[20]. For each trial and contact, the PSD was computed using Welch's method with Hamming windows, spanning 1-40 Hz in 0.5 Hz increments. The PSD was decomposed into aperiodic and oscillatory constituents, with the following parameters: aperiodic_mode='fixed', peak_width_limits=[1, 6], max_n_peaks=6,

peak_threshold=2, and proximity_threshold=1.5. Subtracting the aperiodic fit from the raw PSD revealed periodic peaks in the residual ‘flattened’ spectrum. Peaks with an r-squared value greater than 0.95 and a peak power higher than 0.6 a.u. were retained for further analysis. The frequency distribution of identified peaks was subsequently quantified across a range of experimental conditions in the AIC and hippocampus (Supplementary Figure 17g and h). Consistent with findings from MODAL, spectral peaks in the AIC were predominantly clustered within the theta band across all conditions. In the hippocampus, FOOOF revealed a broader spectral profile, with peaks spanning both theta and alpha bands that were dynamically modulated by behavioral context. These findings establish a spectral foundation for interpreting the phase-tuned modulation described in the main text, underscoring the role of theta rhythms as a stable temporal framework for neural computation during reward processing in both the AIC and hippocampus. This aligns with prior reports that LFOs coordinate neural activity during virtual navigation[4,11,19,21].

Supplementary Table 1. Demographic information. Related to Figure 1.

Participant number	Gender	Age	Handedness	Epileptic zone	Total number of electrodes	Sampling rate (Hz)	Number Electrodes included		Trial numbers (included/excluded)			Sample source
							AIC	Hippocampus	ERZ	PR	EDZ	
1	f	45	Uncertain	Uncertain	74	2048	×	5R2L	33/6	32/7	35/2	Hôpital de la Salpêtrière
2	f	19	Right	Medial temporal lobe (L)	34	1000	×	2L	31/3	30/4	30/4	Hôpital de la Salpêtrière
4	f	21	Right	Cingulate Gyrus (R)	58	4000	2R	×	31/0	31/0	29/3	Hôpital de la Salpêtrière
7	m	37	Right	Temporal lobe (R)	50	4000	1R	×	30/5	30/5	31/4	Hôpital de la Salpêtrière
8	f	18	Right	Temporal Lobe (L)	41	4000	1L	1L	29/6	30/5	29/5	Hôpital de la Salpêtrière
10	m	19	Right	Temporal Lobe (L)	65	4000	×	2L	37/2	38/1	35/3	Hôpital de la Salpêtrière
12	m	29	Left	Uncertain	47	4000	4L	×	30/6	31/5	32/4	Hôpital de la Salpêtrière
13	m	26	Right	Temporal Lobe (R)	66	1000	×	1L	30/5	30/5	30/5	Hôpital de la Salpêtrière
14	m	22	Right	Frontal/Temporal Lobe (R)	64	1000	4R	3R	34/0	34/0	34/0	Hôpital de la Salpêtrière
15	f	29	Left	Medial temporal lobe (L)	82	1000	3R	1R2L	34/2	34/1	35/1	Hôpital de la Salpêtrière
16	m	25	Right	Frontal lobe (R)	53	2000	6R1L	×	30/7	31/5	31/5	Hôpital de la Salpêtrière
17	f	13	Right	Precentral sulci (R)	86	2000	2R	×	34/9	34/8	35/8	Second Affiliated Hospital of Zhejiang University
18	f	27	Right	temporal lobe (L)	54	4096	1L	×	34/1	34/1	33/1	Hôpital de la Salpêtrière

Abbreviations: AIC, anterior insular cortex; ERZ, entering the reward zone; PR, picking up the reward; EDZ, entering the decision zone; R, right hemisphere; L, left hemisphere; f, female; m, male.

Supplementary Table 2. Time difference between the initial pre-activation of reward-specific brain patterns and the peak gamma activity in individual contacts. Related to Figure 3.

Contact	ERZ Trials	Time of Initial Pre-activation		Time of Peak Gamma Activity		Time Lag	
		Mean ± SEM (s)	CV (%)	Mean ± SEM (s)	CV (%)	Mean ± SEM (s)	CV (%)
Participant#4 Contact#1	31	-1.74 ± 0.01	14.33	-0.92 ± 0.02	60.83	-0.83 ± 0.02	69.40
Participant#4 Contact#2	31	-1.23 ± 0.02	41.57	-1.19 ± 0.02	41.58	-0.04 ± 0.02	1875.09
Participant#7 Contact#1	30	-1.81 ± 0.01	9.01	-0.90 ± 0.02	67.15	-0.90 ± 0.02	67.94
Participant#8 Contact#1	29	-1.70 ± 0.01	13.63	-0.84 ± 0.02	60.60	-0.86 ± 0.02	63.29
Participant#12 Contact#1	30	-1.74 ± 0.01	11.20	-1.04 ± 0.02	45.73	-0.69 ± 0.02	82.13
Participant#12 Contact#2	30	-1.79 ± 0.00	8.08	-0.98 ± 0.02	54.39	-0.81 ± 0.02	66.57
Participant#12 Contact#3	30	-1.75 ± 0.01	15.10	-1.09 ± 0.02	46.99	-0.65 ± 0.02	91.63
Participant#12 Contact#4	30	-1.77 ± 0.01	14.05	-0.97 ± 0.02	63.86	-0.80 ± 0.02	89.77
Participant#14 Contact#1	34	-1.76 ± 0.01	10.12	-0.90 ± 0.02	63.96	-0.86 ± 0.02	70.71
Participant#14 Contact#2	34	-1.83 ± 0.00	8.91	-0.99 ± 0.02	60.83	-0.84 ± 0.02	79.36
Participant#14 Contact#3	34	-1.75 ± 0.01	12.26	-0.93 ± 0.02	61.40	-0.82 ± 0.02	81.42
Participant#14 Contact#4	34	-1.76 ± 0.01	10.14	-0.84 ± 0.02	67.62	-0.92 ± 0.02	66.45
Participant#15 Contact#1	34	-1.78 ± 0.00	9.51	-1.02 ± 0.02	52.33	-0.76 ± 0.02	69.49
Participant#15 Contact#2	34	-1.74 ± 0.01	15.00	-0.96 ± 0.02	60.64	-0.78 ± 0.02	70.07
Participant#15 Contact#3	34	-1.65 ± 0.01	20.98	-1.09 ± 0.02	46.81	-0.56 ± 0.02	112.00
Participant#16 Contact#1	30	-1.70 ± 0.01	16.22	-1.04 ± 0.02	61.44	-0.66 ± 0.02	102.90
Participant#16 Contact#2	30	-1.77 ± 0.01	11.34	-1.03 ± 0.02	44.77	-0.74 ± 0.02	62.00
Participant#16 Contact#3	30	-1.73 ± 0.01	19.78	-1.04 ± 0.02	56.55	-0.69 ± 0.02	96.97
Participant#16 Contact#4	30	-1.75 ± 0.01	14.63	-1.20 ± 0.02	50.60	-0.55 ± 0.02	118.64
Participant#16 Contact#5	30	-1.78 ± 0.01	14.05	-0.99 ± 0.02	56.70	-0.79 ± 0.02	70.00
Participant#16 Contact#6	30	-1.73 ± 0.01	17.75	-0.95 ± 0.02	57.74	-0.78 ± 0.02	89.01
Participant#16 Contact#7	30	-1.79 ± 0.01	13.95	-1.14 ± 0.02	50.26	-0.65 ± 0.02	82.56
Participant#17 Contact#1	34	-1.73 ± 0.01	14.79	-1.07 ± 0.02	55.37	-0.66 ± 0.02	99.63
Participant#17 Contact#2	34	-1.65 ± 0.01	16.64	-0.97 ± 0.02	61.70	-0.69 ± 0.02	95.54
Participant#18 Contact#1	34	-1.70 ± 0.01	16.09	-0.89 ± 0.02	66.45	-0.81 ± 0.02	81.04

Abbreviations: ERZ, entering the reward zone; SEM, standard error of the mean; CV, coefficient of variation.

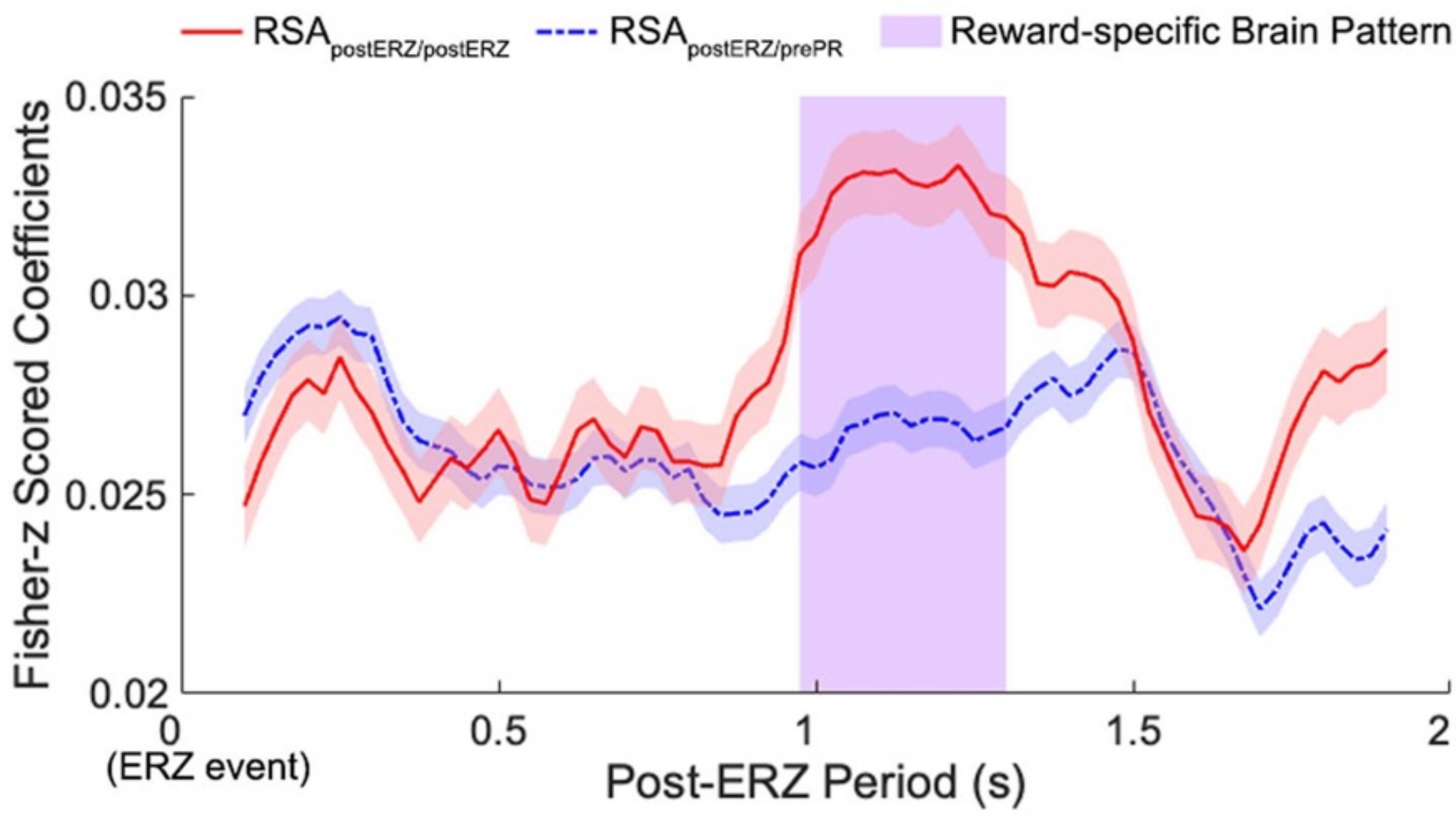

Supplementary Figure 1. Reward-specific brain pattern (RBPs) in the anterior insular cortex. Related to Figure 2. Time-resolved category specificity reveals RBPs during reward viewing. Each line represents time courses of $RSA_{postERZ/postERZ}$ (red; mean ± SEM) or $RSA_{postERZ/prePR}$ (blue). Purple shading indicates time windows carrying RBPs (i.e., $RSA_{postERZ/postERZ} > RSA_{postERZ/prePR}$). X-axis denotes time courses relative to post-ERZ periods, and time 0 indicates the onset of rewards (i.e., ERZ events). ERZ, entering the reward zone; PR, picking up the reward.

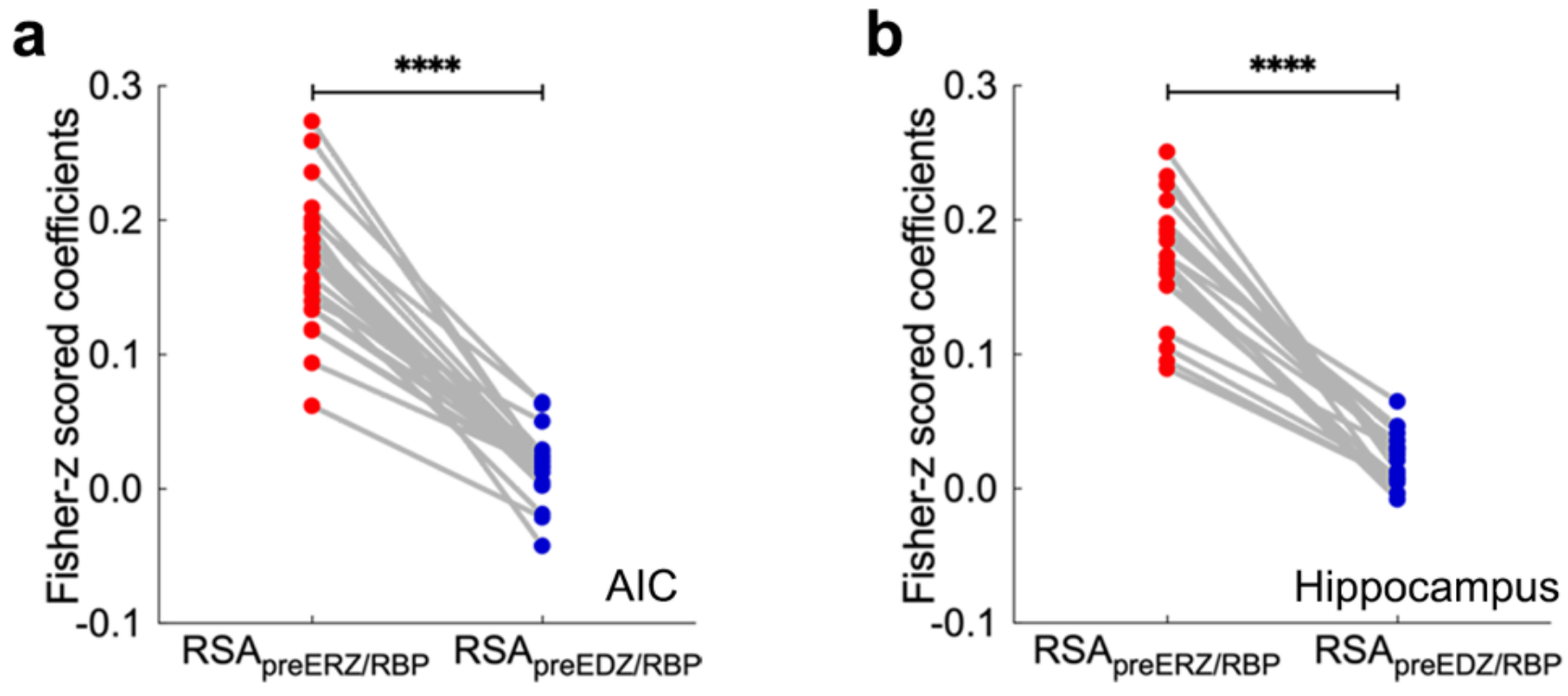

Supplementary Figure 2. Differential RBP pre-activation during reward versus decision context at the contact level. Related to Figure 2. (a and b) Maximal pre-activation of reward-specific brain patterns (RBPs) was significantly higher for $RSA_{preERZ/RBP}$ (red) than for $RSA_{preEDZ/RBP}$ (blue) in both the anterior insular cortex (**a**) and hippocampus **(b)**. Dots represent individual contacts. AIC, anterior insular cortex.****, p<0.001.

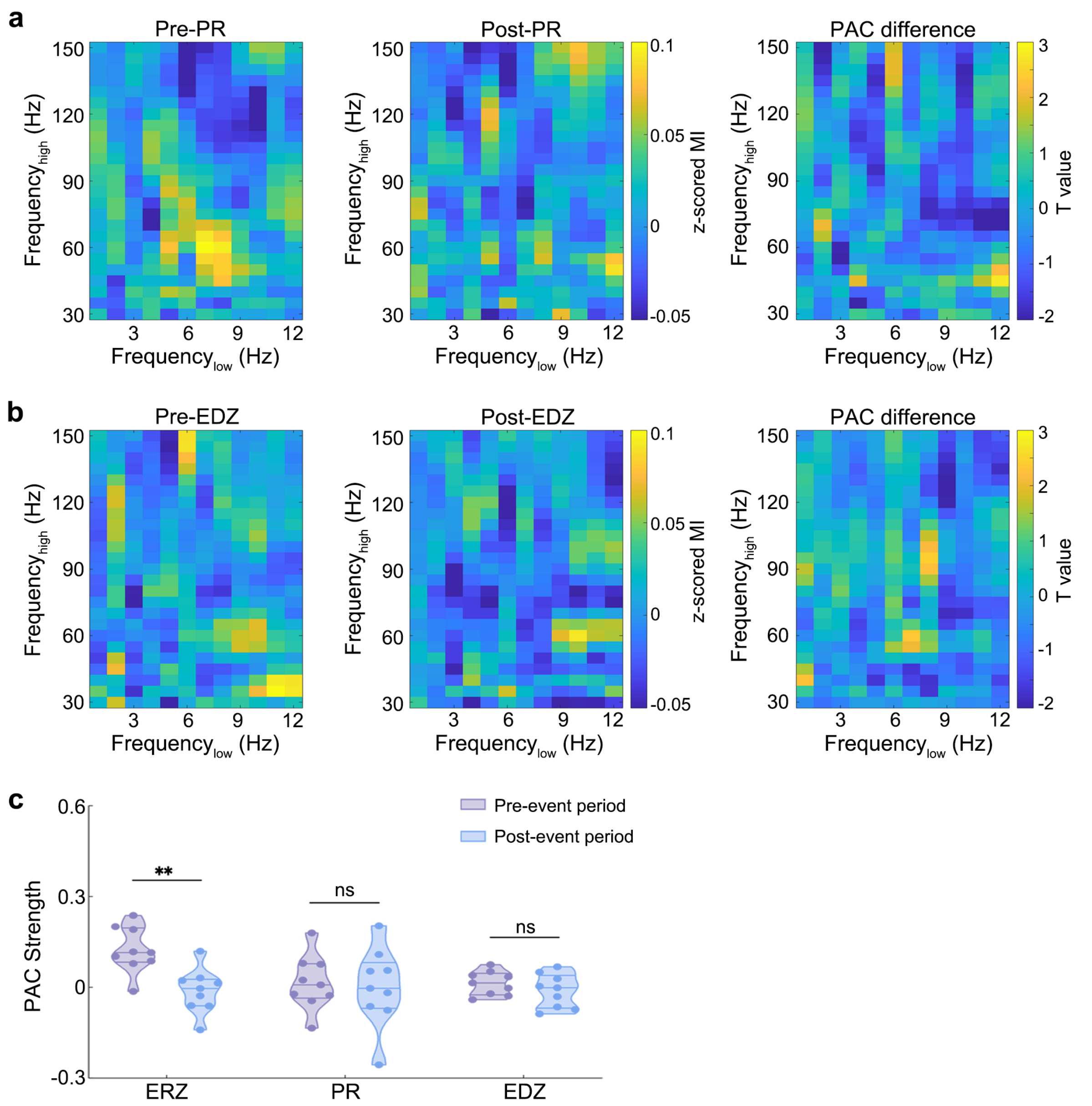

Supplementary Figure 3. Phase-amplitude coupling (PAC) difference between before and after experimental events in AIC contacts. Related to Figure 3. (a and b) Comodulograms illustrate the z-scored modulation index (MI) during pre-event and post-event periods, as well as the difference in PAC, for both PR **(a)** and EDZ **(b)** events. PR, picking up the reward; EDZ, entering the decision zone. **(c)** Average PAC values within the significant cluster identified in Figure 3b are shown for individual participants, comparing pre- and post-event periods for entering the reward zone (ERZ), picking up the reward (PR) and entering the decision zone (EDZ). Purple indicates pre-event period, and blue indicate post-event period. Each violin plot represents one condition, and dots represent individual participants. **, $0.005 \leq p < 0.01$; ns, no significance.

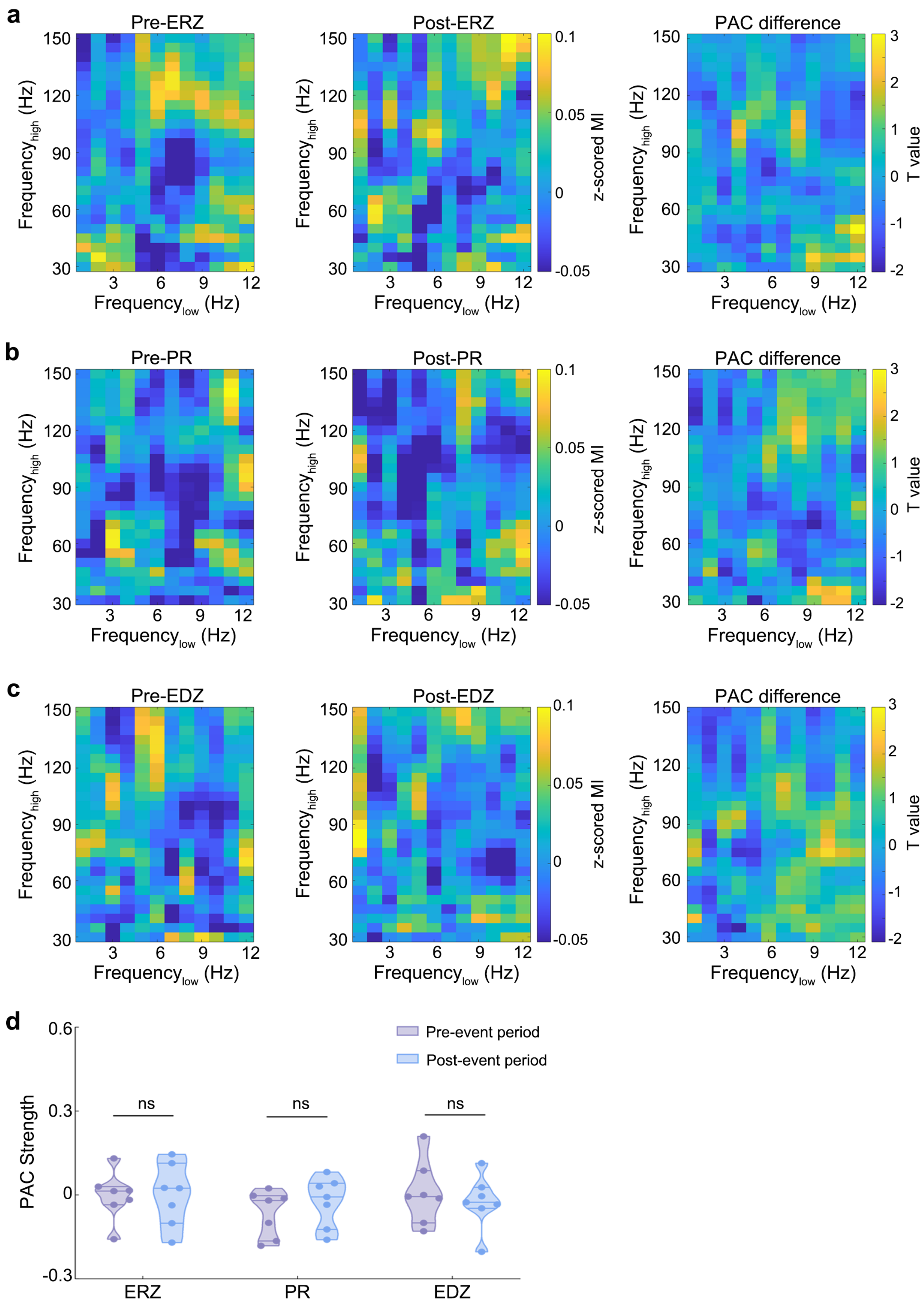

Supplementary Figure 4. Phase-amplitude coupling (PAC) difference between before and after experimental events in hippocampal contacts. Related to Figure 3. (a-c) Comodulograms illustrate the z-scored modulation index (MI) during pre-event and post-event periods, as well as the difference in PAC, for both

ERZ **(a),** PR **(b)** and EDZ **(c)** events. ERZ, entering the reward zone; PR, picking up the reward; EDZ, entering the decision zone. **(d)** Average PAC values within the significant cluster identified in Figure 3b are shown for individual participants, comparing pre- and post-event periods for ERZ, PR and EDZ events. Purple indicates pre-event period, and blue indicate post-event period. Each violin plot represents one condition, and dots represent individual participants. ns, no significance.

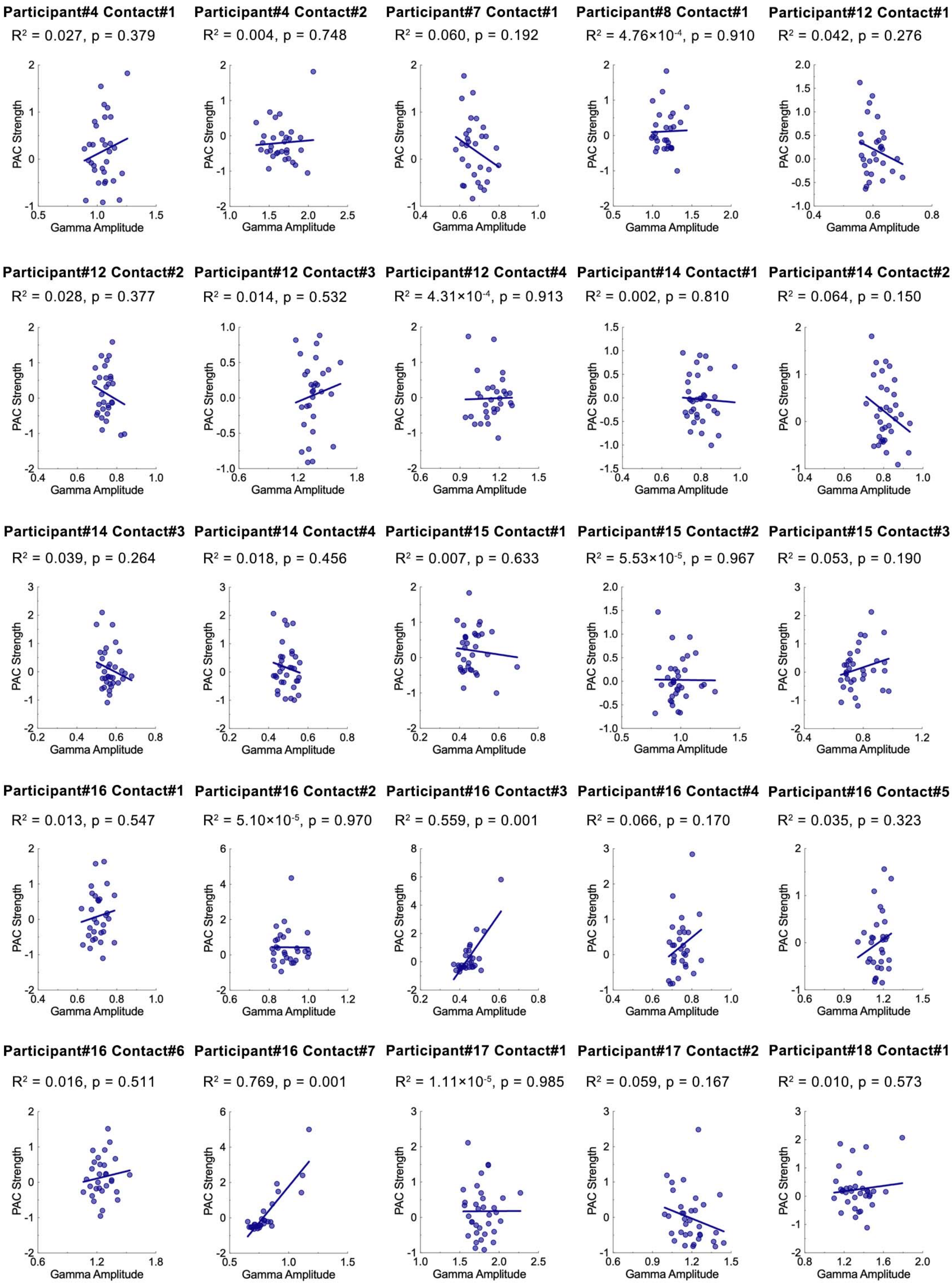

Supplementary Figure 5. Theta–gamma PAC exhibits substantial independence from gamma-band amplitude fluctuations. Across most anterior insular contacts (23/25), theta-gamma phase-amplitude coupling (PAC) strength was not significantly correlated with gamma-band amplitude. Each panel represents one recording contact; blue dots indicate individual trials.

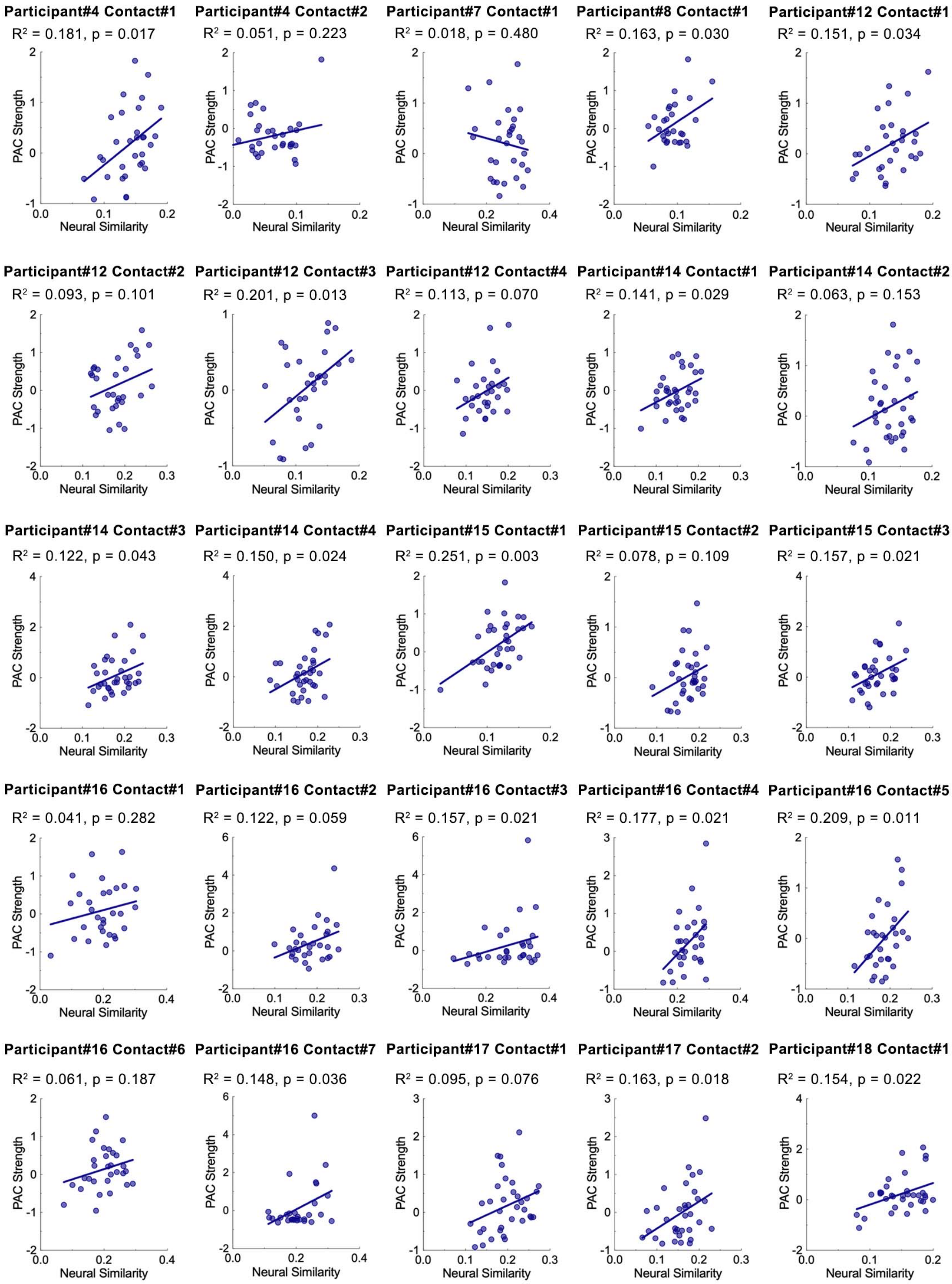

Supplementary Figure 6. Theta–gamma PAC exhibits positive correlation with RBP pre-activation level. Relate to Figure 3. A significant positive correlation between the strength of phase-amplitude coupling (PAC) and the pre-activation level of reward-specific pattern (RBP) was observed in majority (15/25) of anterior insular contacts. Each panel represents one recording contact; blue dots indicate individual trials.

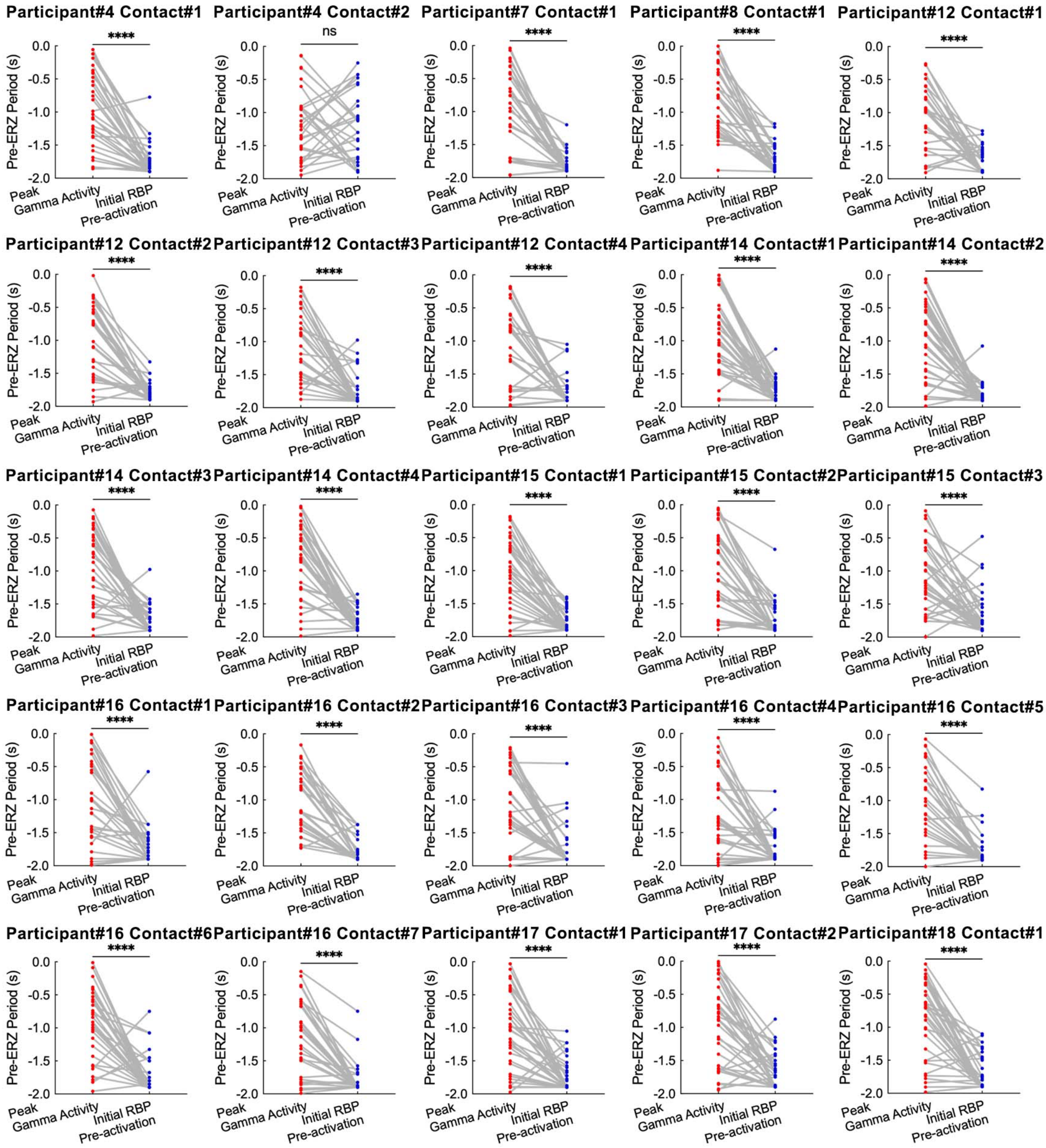

Supplementary Figure 7. Temporal sequence of RBP pre-activation and PAC effect at the trial level. Related to Figure 3. Trial-wise time points of peak gamma activity (red) and initial RBP pre-activation (blue) within each contact. Each panel represents one contact, and dots indicate individual trials. Y-axis shows time relative to pre-ERZ periods; time 0 represents reward onset. ****, $p < 0.001$; ns, not significant.

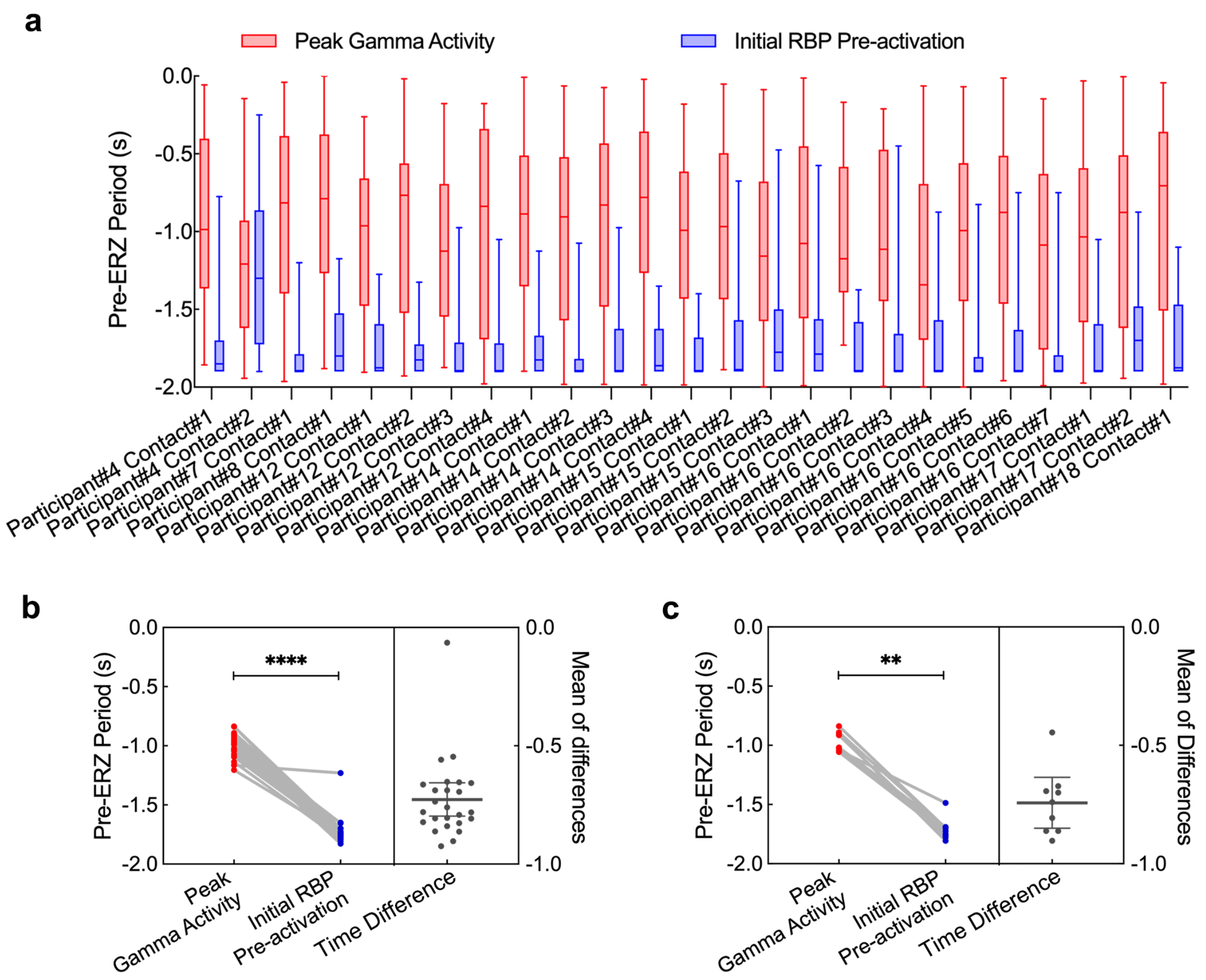

Supplementary Figure 8. Temporal sequence of RBP pre-activation and PAC effect at the contact and participant level. Related to Figure 3. (a) The initial pre-activation of reward-specific brain patterns (RBPs, blue) precedes the peak gamma activity (red) at the preferred theta phase (derived from phase-amplitude coupling, PAC) in the 24 out of 25 contacts. Box plots indicate the median (central line), 25th-75th percentiles (box), and whiskers from minimum to maximum across trials for each contact (n = 25 contacts). **(b)** Time difference between peak gamma activity and initial RBP pre-activation across contacts. **(c)** Time difference between peak gamma activity and initial RBP pre-activation across participants. Y axis indicates the time course relative to pre-ERZ periods, and time 0 indicates reward onset. **, $0.005 \le p < 0.01$; ****, $p < 0.001$.

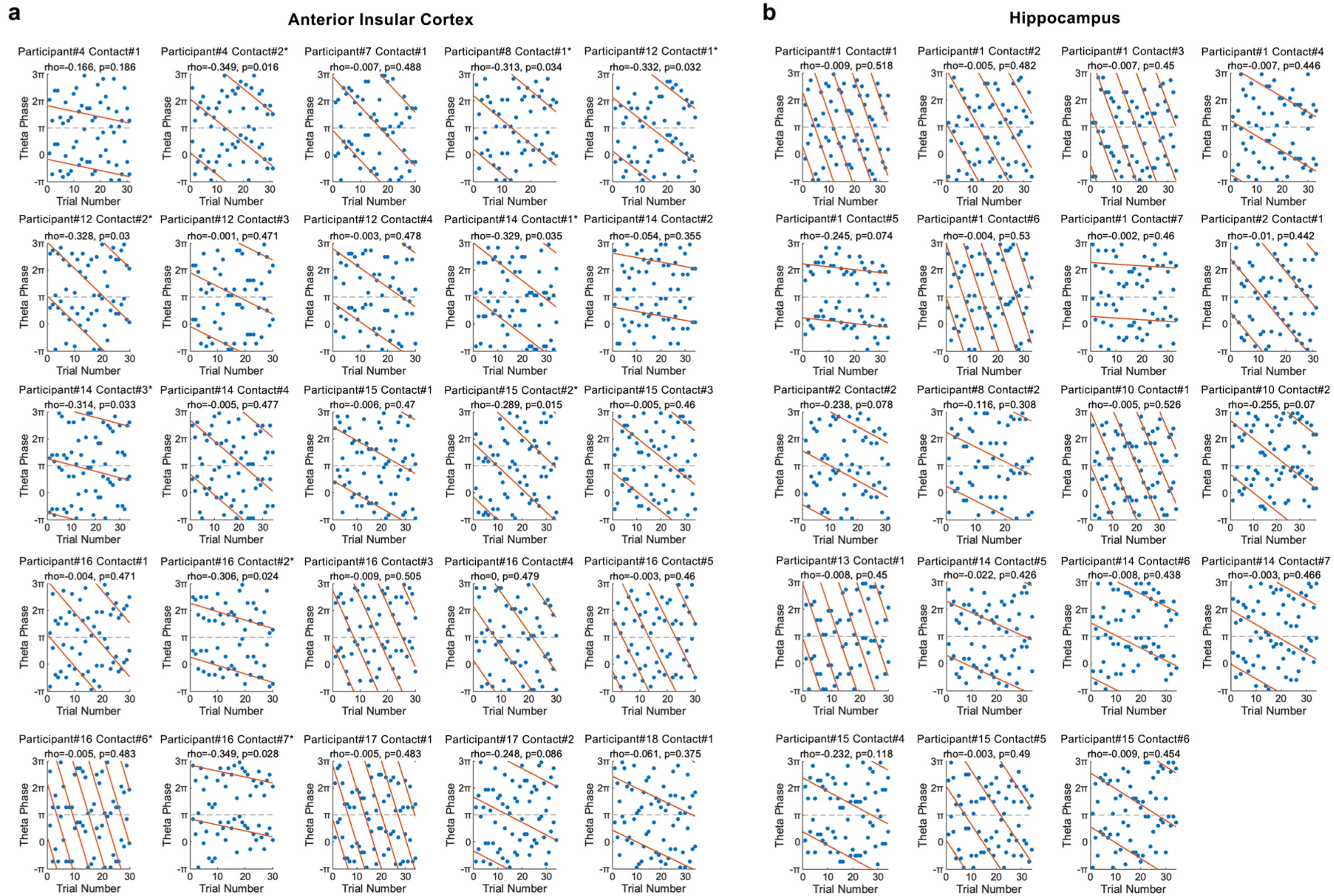

Supplementary Figure 9. PPLE before entering reward zones in AIC and hippocampal contacts. Related to Figure 4. (a and b) Phase-precession-like effect (PPLE) in the anterior insular cortex **(a)** and hippocampus **(b)**, showing the progressive advancement of preferred gamma-coupled theta phases across navigation rounds before entering the reward zones. Each panel represents one contact, blue dots indicate individual navigation rounds and orange lines represent circular-linear regression fits. Asterisks denote significant effects.

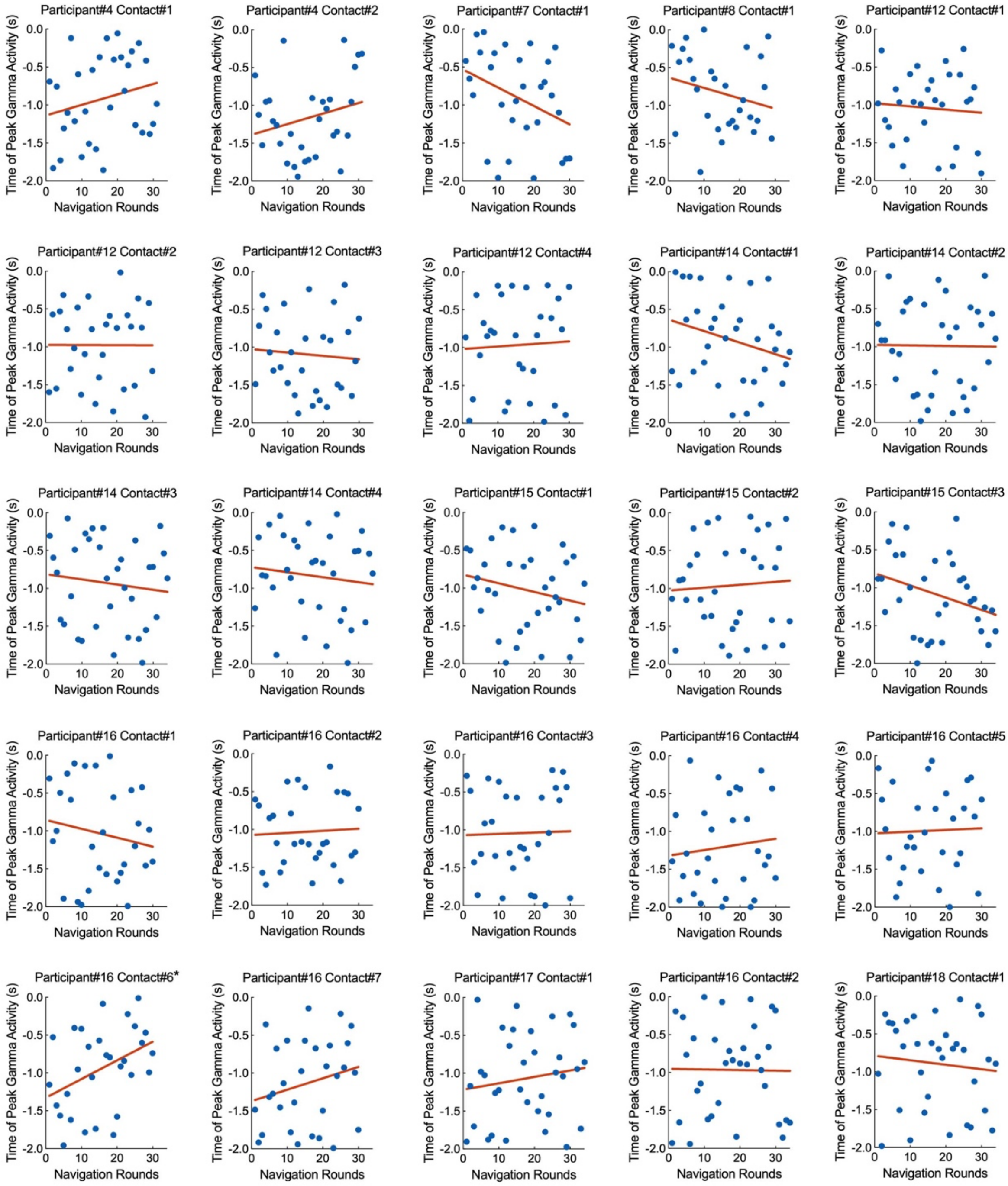

Supplementary Figure 10. Relationship between the time of peak gamma activity and the number of navigation rounds in AIC contacts. For each anterior insular cortex (AIC) contact, the time of peak gamma activity (derived from phase-amplitude coupling analysis) before entering the reward zones (ERZ event) is plotted against navigation rounds. Each panel represents one contact, blue dots indicate individual navigation rounds and orange lines represent linear regression fits. Y axis indicates the time course relative to pre-ERZ periods, with time 0 representing the ERZ event. Asterisks denote significant effects.

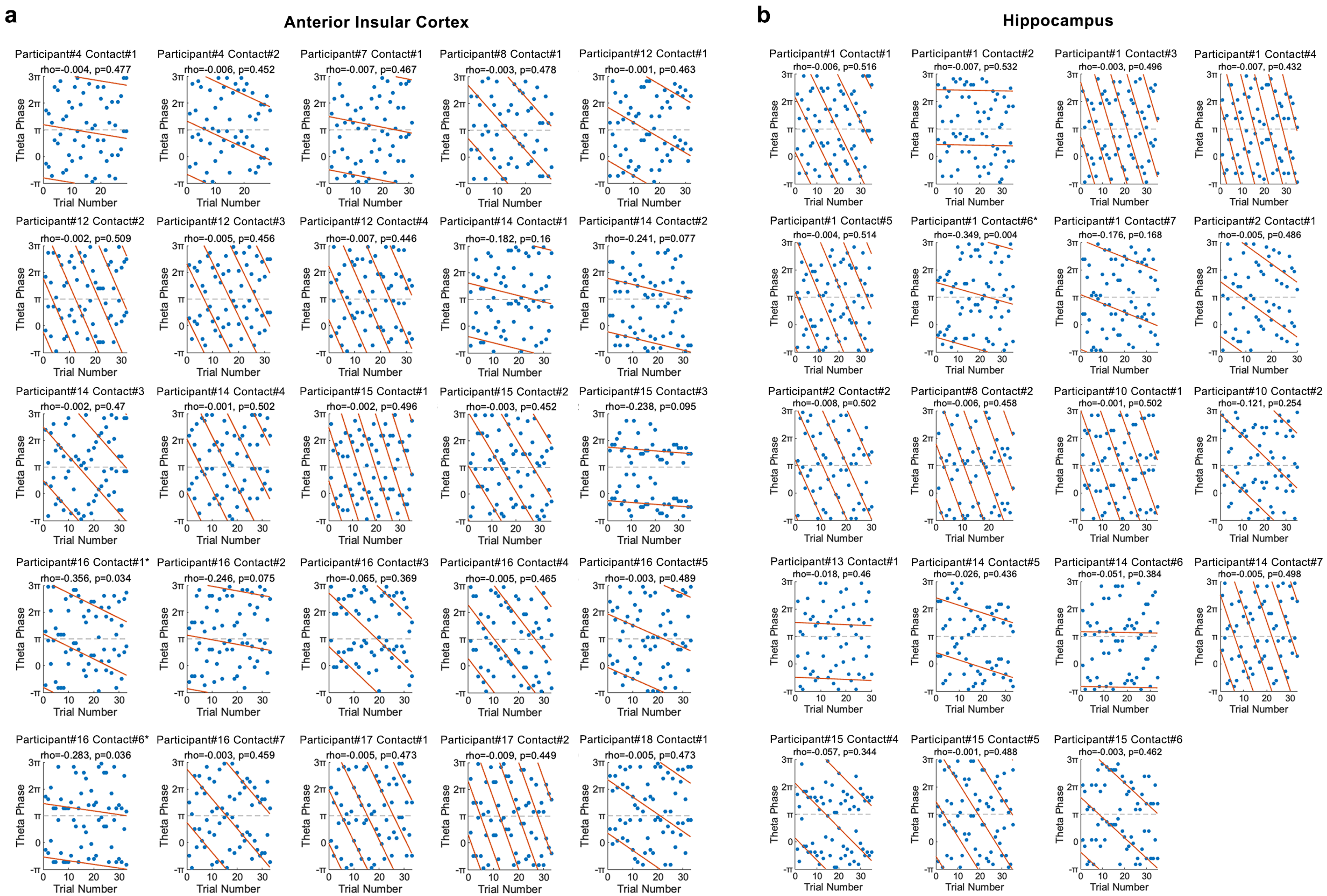

Supplementary Figure 11. PPLE before entering decision zones in AIC and hippocampal contacts. Related to Figure 4. (a and b) Phase-precession-like effect (PPLE) in the anterior insular cortex **(a)** and hippocampus **(b)**, showing the progressive advancement of preferred gamma-coupled theta phases across navigation rounds before entering the decision zones. Each panel represents one contact, blue dots indicate individual navigation rounds and orange lines represent circular-linear regression fits. Asterisks denote significant effects.

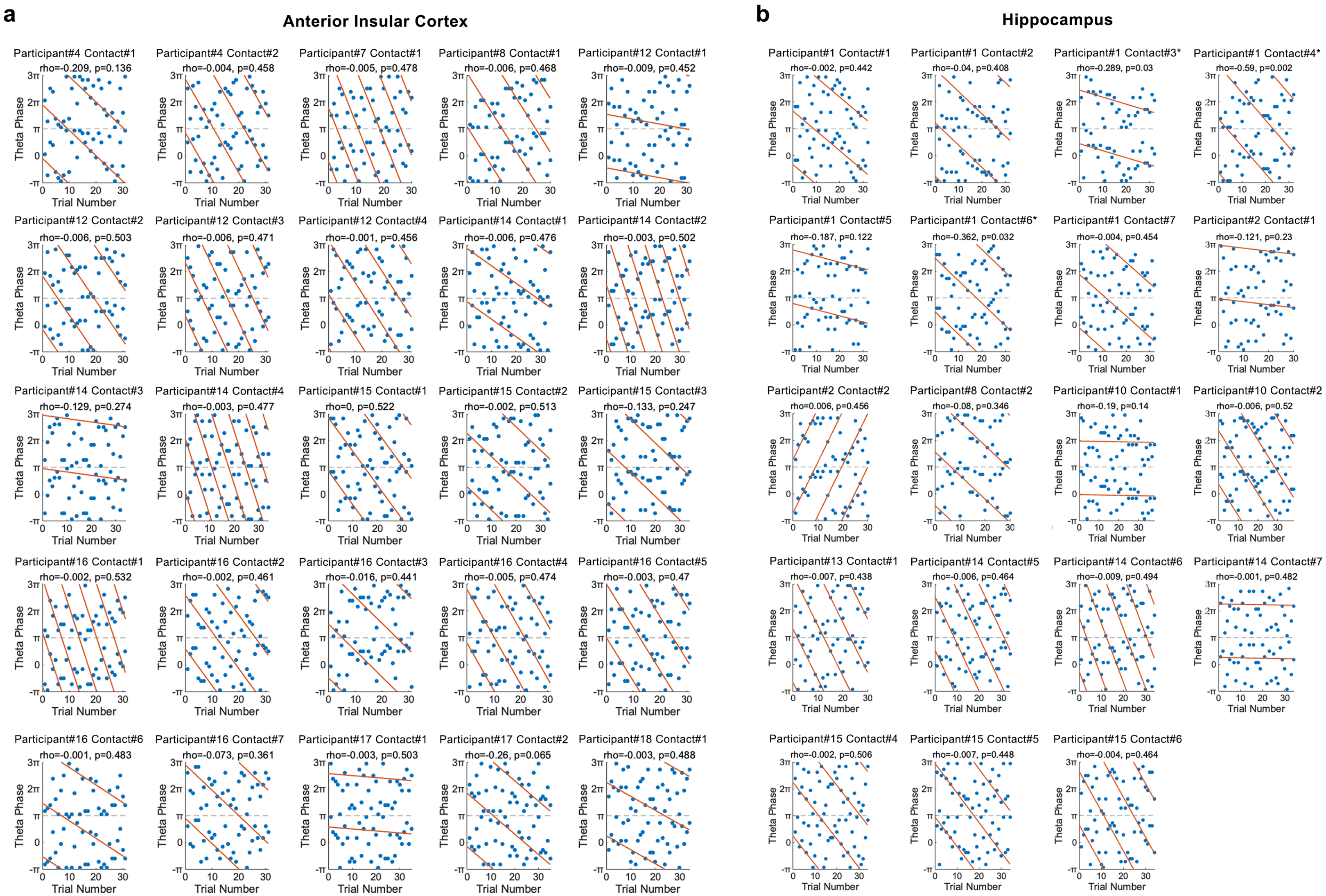

Supplementary Figure 12. PPLE before picking up rewards in AIC and hippocampal contacts. Related to Figure 4. (a and b) Phase-precession-like effect (PPLE) in the anterior insular cortex **(a)** and hippocampus **(b)**, showing the progressive advancement of preferred gamma-coupled theta phases across navigation rounds before picking up the rewards. Each panel represents one contact, blue dots indicate individual navigation rounds and orange lines represent circular-linear regression fits. Asterisks denote significant effects.

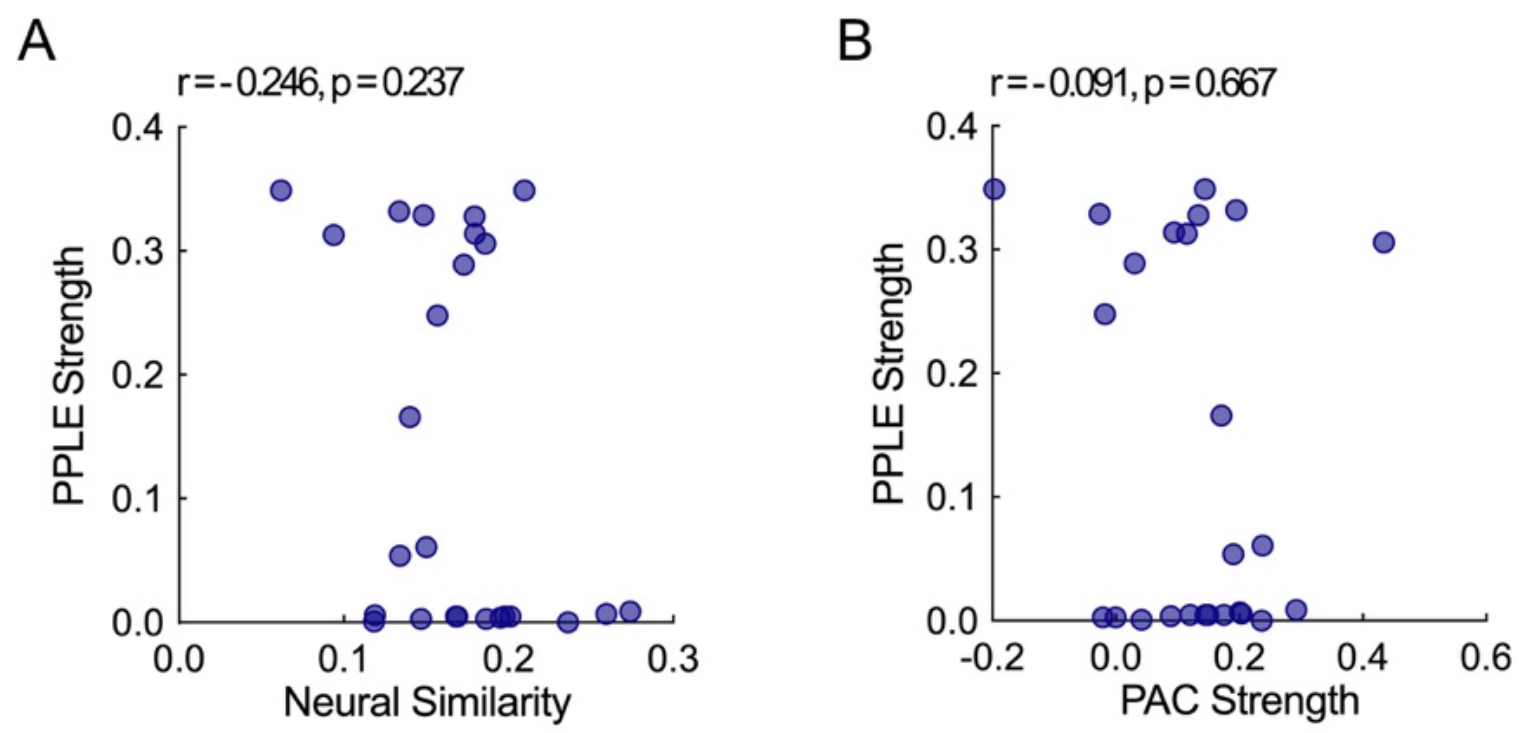

Supplementary Figure 13. The PPLE strength did not demonstrate a significant correlation with either RBP pre-activation level (a) or PAC strength (b). Each blue sphere represents one recording contact.

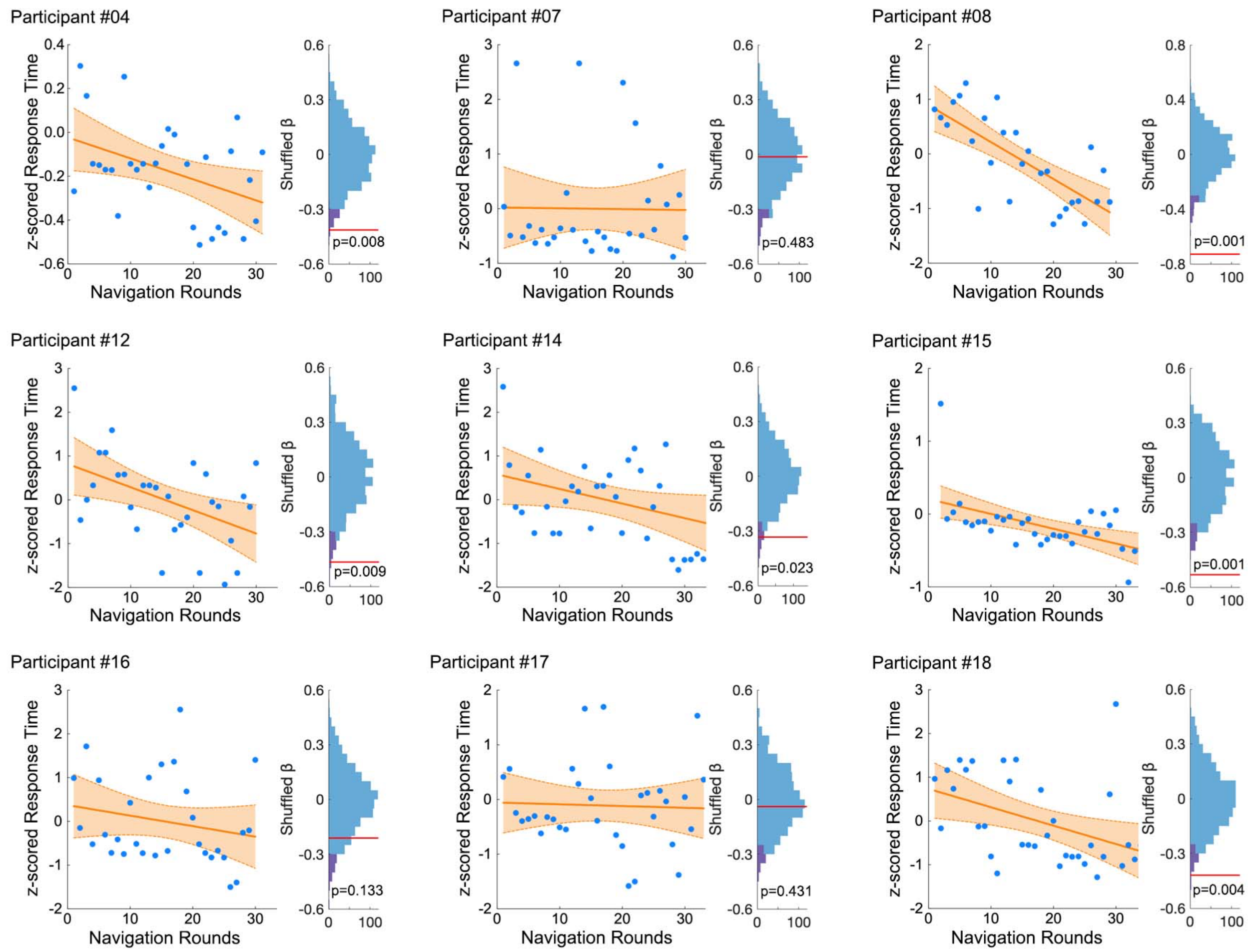

Supplementary Figure 14. Response time acceleration across navigation rounds in participants with AIC contacts. Related to Figure 1 and 5. Relationship between z-scored response times (RT) and number of navigation rounds for nine participants with contacts in the anterior insular cortex (AIC). Each scatter-plot represents one participant, each dot represents one navigation round. Orange lines indicates the fitted regression lines, and shaded area represents the 95% confidence interval. To the right of each scatter plot, a bar-plot displays the rank of the empirical data (indicated by the red vertical line) within the surrogate distribution for each participant. Purple shaded area indicates the significance level.

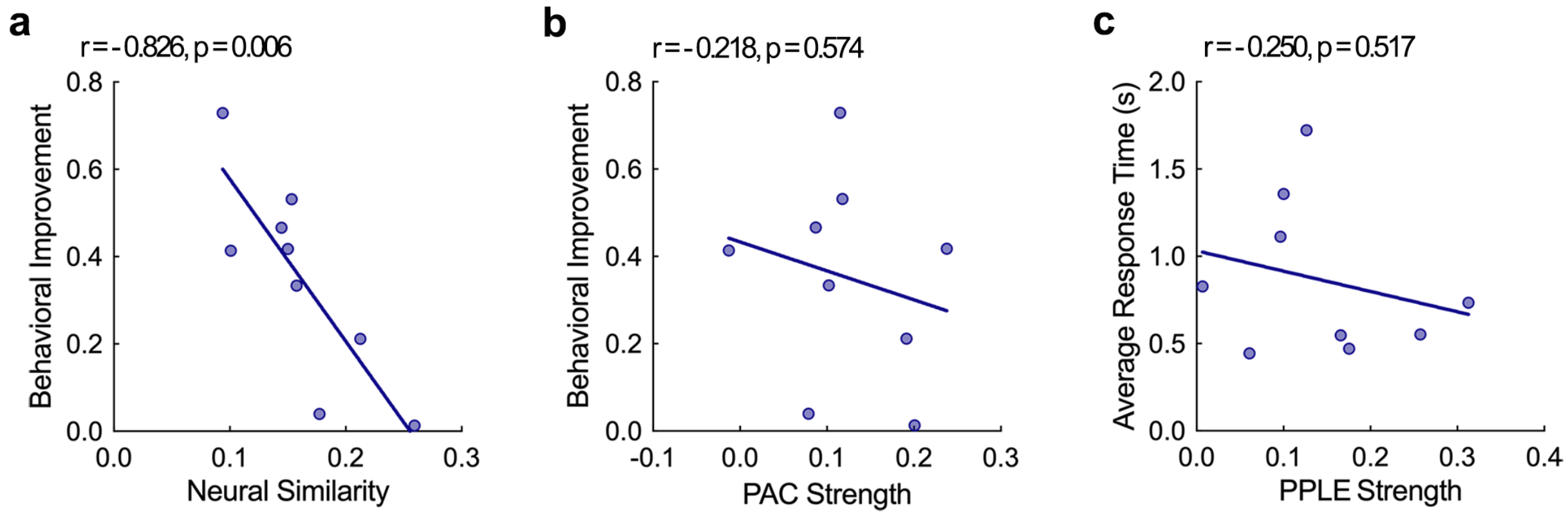

Supplementary Figure 15. Relationship between behavioral performance and identified neural signatures. Relation to Figure 5. (a) Behavioral improvement demonstrates a strong negative correlation with average pre-activation level of reward-specific brain pattern (RBP). **(b)** No significant correlation was observed between behavioral improvement and the average strength of phase-amplitude coupling (PAC). **(c)** There was no correlation between the average response time and the strength of phase-precession-like effect (PPLE). Each blue sphere represents one recording contact.

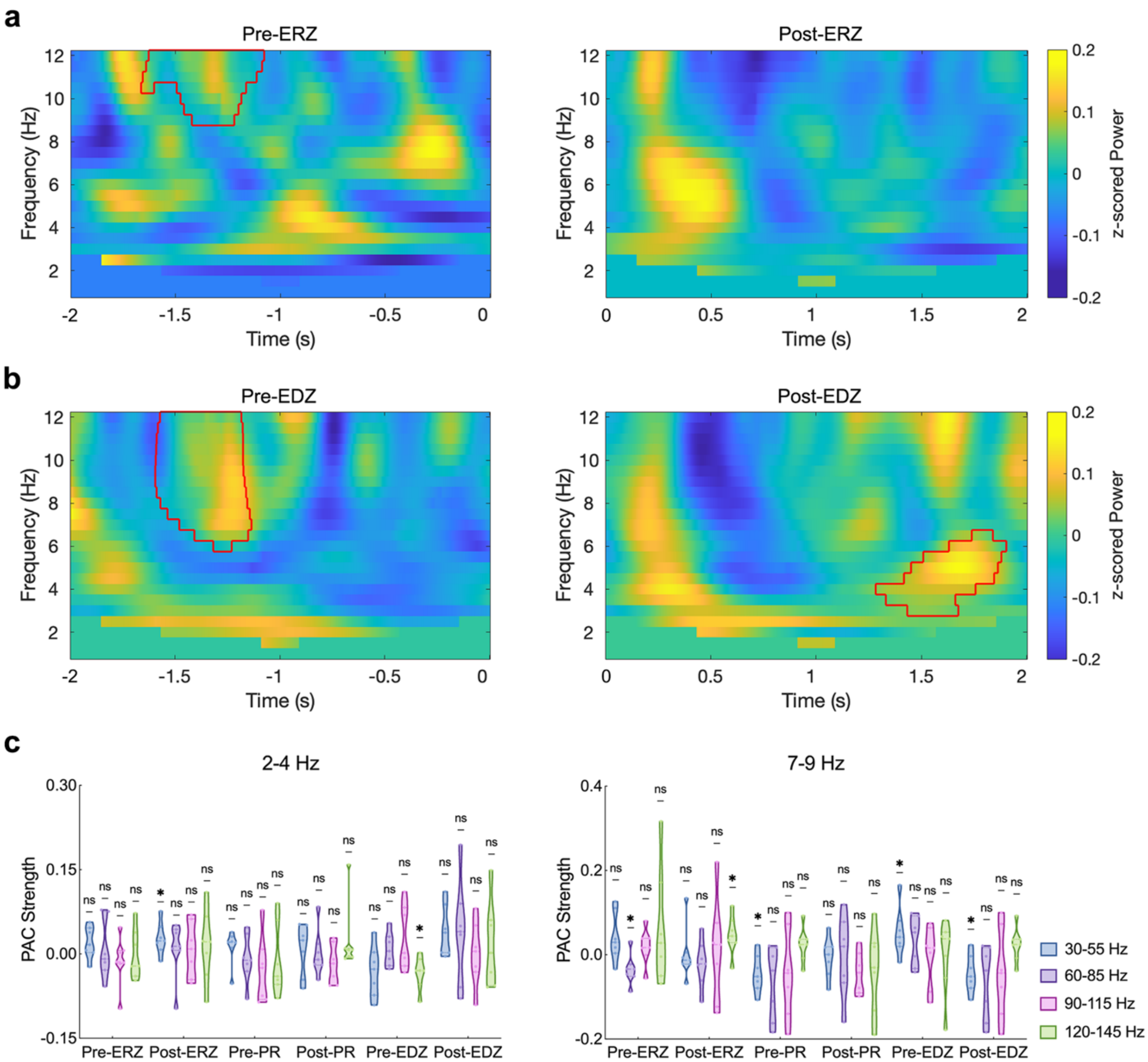

Supplementary Figure 16. Theta activity and phase-amplitude coupling (PAC) in the hippocampus. Relate to Figure 3. (a) Theta activity before and after entering the reward zone (ERZ). Red contours indicate a time-frequency cluster showing significantly increased theta power. X-axis denotes time courses relative to ERZ periods, and time 0 indicates the onset of rewards. **(b)** Theta power before and after entering the decision zone (EDZ). Red contours indicate a time-frequency cluster showing significantly enhanced theta activity. X-axis denotes time courses relative to EDZ periods, and time 0 indicates the onset of decision cues. **(c)** Average PAC strength between two low-frequency bands (2-4Hz and 7-9Hz) and four gamma sub-bands. Each violin represents one frequency pair, dots represent individual participants. *, $0.01 \leq p < 0.05$; ns, not significant.

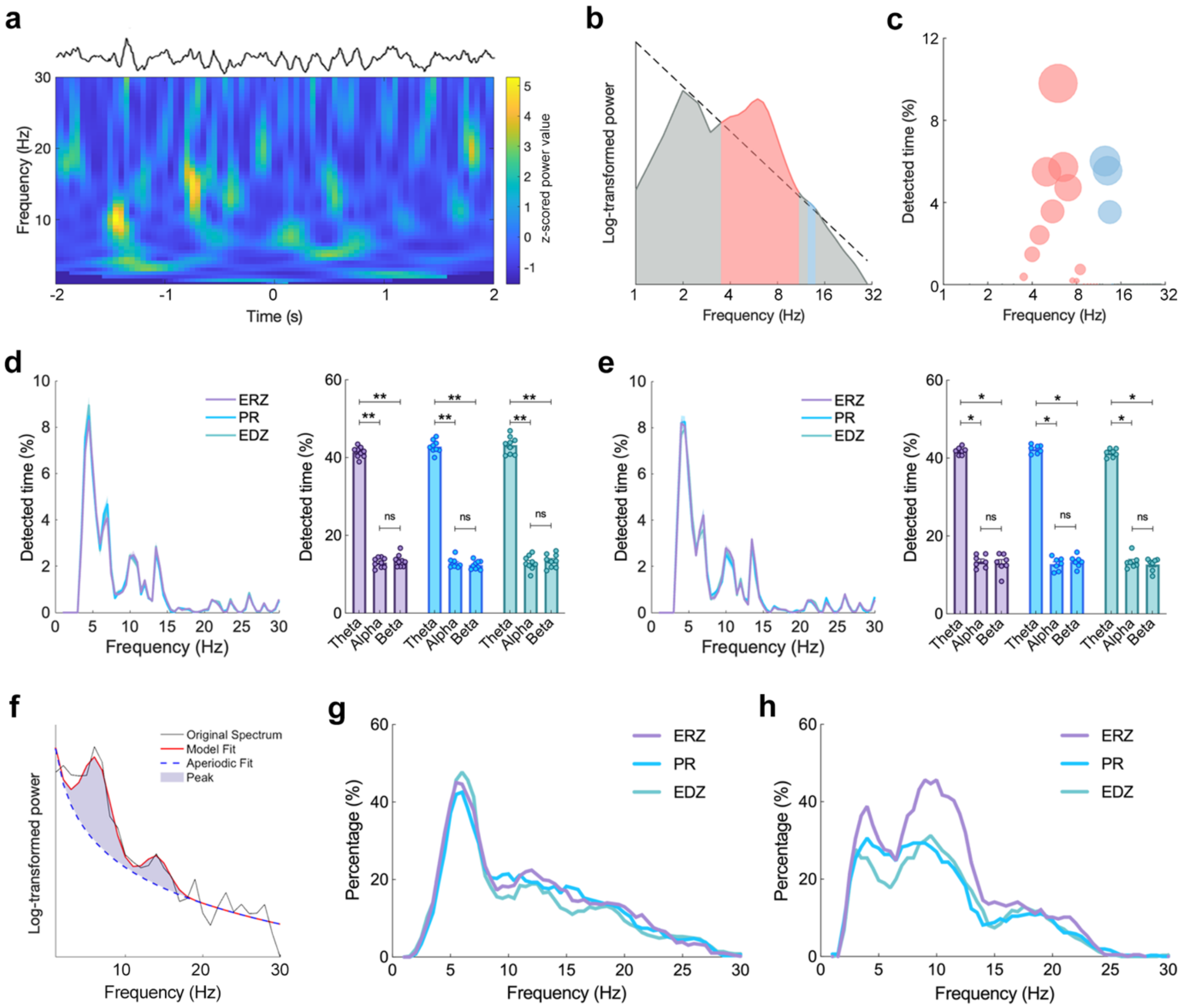

Supplementary Figure 17. Spectral characterization of low-frequency oscillations in the AIC and hippocampus during virtual navigation. (a-c) An example trial from a recording contact in the right anterior insular cortex (AIC) of participant #16. **(a)** Raw trace anda its time-frequency spectrum in the example trial. Time 0 indicates the timepoint of entering the reward zone. **(b)** Two narrow-band oscillations (3.5-11 Hz indicated by pink; 12.5-14 Hz indicated by light blue) are identified as contiguous frequencies exceeding the fitted line (dashed line) using the Multiple oscillations detection algorithm (MODAL). **(c)** Prevalence of oscillations at each frequency from the trial shown in panel B. The larger the sphere, the higher the percentage of detected time of the corresponding frequency. **(d-e)** Population-level oscillation prevalence in AIC **(d)** and hippocampal **(e)** contacts. Left: Percentage of time periods during which oscillatory activities are detected separately for each frequency. Right: Proportion of time periods for each detected oscillation band (theta: 3.5-7.5 Hz; alpha: 8-12 Hz; beta: 12.5-30 Hz). Rings represent individual participant, and error bars indicate one standard deviation (SD) from the mean. *, $0.01 \leq p < 0.05$; **, $0.005 \leq p < 0.01$; ns, no significance. **(f)** Example spectral decomposition using the Fitting Oscillations & One Over F (FOOOF) model. Narrow-band oscillations (light purple) are identified in the residual flattened spectrum after subtracting the aperiodic component (dashed line). **(g-h)** Distribution of spectral peak frequencies identified by FOOOF across experimental conditions in the AIC **(g)** and hippocampus **(h)**. Each color denotes one experimental condition. ERZ, enter the reward zone; PR, picking up the reward; EDZ, enter the decision zone.

Supplementary References

1 van Driel, J., Cox, R. & Cohen, M. X. Phase-clustering bias in phase-amplitude cross-frequency coupling and its removal. J Neurosci Methods **254**, 60-72, doi:10.1016/j.jneumeth.2015.07.014 (2015).

2 Sławińska, U. & Kasicki, S. The frequency of rat's hippocampal theta rhythm is related to the speed of locomotion. Brain Res **796**, 327-331, doi:10.1016/s0006-8993(98)00390-4 (1998).

3 Goyal, A. et al. Functionally distinct high and low theta oscillations in the human hippocampus. Nat Commun **11**, 2469, doi:10.1038/s41467-020-15670-6 (2020).

4 Bohbot, V. D., Copara, M. S., Gotman, J. & Ekstrom, A. D. Low-frequency theta oscillations in the human hippocampus during real-world and virtual navigation. Nat Commun **8**, 14415, doi:10.1038/ncomms14415 (2017).

5 Ekstrom, A. D. et al. Human hippocampal theta activity during virtual navigation. Hippocampus **15**, 881-889, doi:10.1002/hipo.20109 (2005).

6 Watrous, A. J., Fried, I. & Ekstrom, A. D. Behavioral correlates of human hippocampal delta and theta oscillations during navigation. J Neurophysiol **105**, 1747-1755, doi:10.1152/jn.00921.2010 (2011).

7 Bush, D. et al. Human hippocampal theta power indicates movement onset and distance travelled. Proc Natl Acad Sci U S A **114**, 12297-12302, doi:10.1073/pnas.1708716114 (2017).

8 Stangl, M. et al. Boundary-anchored neural mechanisms of location-encoding for self and others. Nature **589**, 420-425, doi:10.1038/s41586-020-03073-y (2021).

9 Kaplan, R. et al. Human hippocampal theta oscillations reflect sequential dependencies during spatial planning. Cogn Neurosci **11**, 122-131, doi:10.1080/17588928.2019.1676711 (2020).

10 Guitart-Masip, M. et al. Synchronization of medial temporal lobe and prefrontal rhythms in human decision making. J Neurosci **33**, 442-451, doi:10.1523/jneurosci.2573-12.2013 (2013).

11 Mao, D. Neural Correlates of Spatial Navigation in Primate Hippocampus. Neurosci Bull **39**, 315-327, doi:10.1007/s12264-022-00968-w (2023).

12 Zhang, H. & Jacobs, J. Traveling Theta Waves in the Human Hippocampus. The Journal of Neuroscience **35**, 12477-12487, doi:10.1523/jneurosci.5102-14.2015 (2015).

13 Zhang, H. in Intracranial EEG: A Guide for Cognitive Neuroscientists (ed Nikolai Axmacher) 567-577 (Springer International Publishing, 2023).

14 Tort, A. B., Komorowski, R. W., Manns, J. R., Kopell, N. J. & Eichenbaum, H. Theta-gamma coupling increases during the learning of item-context associations. Proc Natl Acad Sci U S A **106**, 20942-20947, doi:10.1073/pnas.0911331106 (2009).

15 Tort, A. B., Komorowski, R., Eichenbaum, H. & Kopell, N. Measuring phase-amplitude coupling between neuronal oscillations of different frequencies. J Neurophysiol **104**, 1195-1210, doi:10.1152/jn.00106.2010 (2010).

16 Axmacher, N. et al. Cross-frequency coupling supports multi-item working memory in the human hippocampus. Proc Natl Acad Sci U S A **107**, 3228-3233, doi:10.1073/pnas.0911531107 (2010).

17 Daume, J. et al. Control of working memory by phase-amplitude coupling of human hippocampal neurons. Nature **629**, 393-401, doi:10.1038/s41586-024-07309-z (2024).

18 Vivekananda, U. et al. Theta power and theta-gamma coupling support long-term spatial memory retrieval. Hippocampus **31**, 213-220, doi:10.1002/hipo.23284 (2021).

19 Watrous, A. J., Miller, J., Qasim, S. E., Fried, I. & Jacobs, J. Phase-tuned neuronal firing encodes human contextual representations for navigational goals. Elife **7**, doi:10.7554/eLife.32554 (2018).

20 Donoghue, T. et al. Parameterizing neural power spectra into periodic and aperiodic components. Nat Neurosci **23**, 1655-1665, doi:10.1038/s41593-020-00744-x (2020).

21 Kahana, M. J., Sekuler, R., Caplan, J. B., Kirschen, M. & Madsen, J. R. Human theta oscillations exhibit task dependence during virtual maze navigation. Nature **399**, 781-784, doi:10.1038/21645 (1999).
