# Supplementary material for: Phase-tuned modulation during reward expectancy in human anterior insular cortex
Source: Commun Biol. 2026 Jun 16;9:1148. doi: 10.1038/s42003-026-10427-1 (PMC13521940; doi:10.1038/s42003-026-10427-1)
Supplement: Supplementary file 2 [file 42003_2026_10427_MOESM2_ESM.pdf]

nature portfolio

Corresponding author(s): Hui Zhang, Shuang Wang

Last updated by author(s): May 16, 2026

Reporting Summary

Nature Portfolio wishes to improve the reproducibility of the work that we publish. This form provides structure for consistency and transparency in reporting. For further information on Nature Portfolio policies, see our Editorial Policies and the Editorial Policy Checklist.

Statistics

For all statistical analyses, confirm that the following items are present in the figure legend, table legend, main text, or Methods section.

n/a	Confirmed	
☐	☒	The exact sample size (n) for each experimental group/condition, given as a discrete number and unit of measurement
☐	☒	A statement on whether measurements were taken from distinct samples or whether the same sample was measured repeatedly
☐	☒	The statistical test(s) used AND whether they are one- or two-sided<br>*Only common tests should be described solely by name; describe more complex techniques in the Methods section.*
☐	☒	A description of all covariates tested
☐	☒	A description of any assumptions or corrections, such as tests of normality and adjustment for multiple comparisons
☐	☒	A full description of the statistical parameters including central tendency (e.g. means) or other basic estimates (e.g. regression coefficient) AND variation (e.g. standard deviation) or associated estimates of uncertainty (e.g. confidence intervals)
☐	☒	For null hypothesis testing, the test statistic (e.g. F, t, r) with confidence intervals, effect sizes, degrees of freedom and P value noted<br>*Give P values as exact values whenever suitable.*
☒	☐	For Bayesian analysis, information on the choice of priors and Markov chain Monte Carlo settings
☒	☐	For hierarchical and complex designs, identification of the appropriate level for tests and full reporting of outcomes
☐	☒	Estimates of effect sizes (e.g. Cohen's d, Pearson's r), indicating how they were calculated

Our web collection on statistics for biologists contains articles on many of the points above.

Software and code

Policy information about availability of computer code

Data collection	IEEG data was acquired using an Ad-Tech system (Hôpital de la Salpêtrière) and a Nihon-Kohden recording system (Second Affiliated Hospital of Zhejiang University).
Data analysis	Custom code written in MATLAB version R2022b (the Mathworks, Natick, MA, USA), FieldTrip functions (Fieldtrip Toolbox 20230520, https://www.fieldtriptoolbox.org/), EEGLAB toolbox (version eeglab 14_1_Ob, https:// sccn.ucsd.edu/eeglab/index.php) and Python 3.10.9 were used to analyze all data.

For manuscripts utilizing custom algorithms or software that are central to the research but not yet described in published literature, software must be made available to editors and reviewers. We strongly encourage code deposition in a community repository (e.g. GitHub). See the Nature Portfolio guidelines for submitting code & software for further information.

Data

Policy information about availability of data

All manuscripts must include a data availability statement. This statement should provide the following information, where applicable:

- Accession codes, unique identifiers, or web links for publicly available datasets
- A description of any restrictions on data availability
- For clinical datasets or third party data, please ensure that the statement adheres to our policy

The raw iEEG data are confidential medical records and cannot be publicly deposited due to ethical and privacy restrictions. All numerical data used to generate the

data shown are available at the Figshare repository (https://doi.org/10.6084/m9.figshare.30413173). Additional data that support the findings of this study are available from the corresponding author upon reasonable request.

Research involving human participants, their data, or biological material

Policy information about studies with human participants or human data. See also policy information about sex, gender (identity/presentation), and sexual orientation and race, ethnicity and racism.

Reporting on sex and gender	Gender was self-reported by participants and not included as covariates in the analyses.
Reporting on race, ethnicity, or other socially relevant groupings	They were not requested or reported and not included as covariates in the analyses.
Population characteristics	We included 13 epilepsy patients with implanted electrodes (six males; average age = 25.25 ± 8.87 years [mean ± SD]). All participants had normal or corrected-to-normal visual acuity, normal color vision, and no MRI contraindications.
Recruitment	We included all patients that accepted to participate in the study and that had electrodes implanted in the anterior insular cortex and hippocampus. Implantation sites were chosen solely on the basis of clinical criteria.
Ethics oversight	The study was approved by the Medical Ethics Committee at the hospital sites (INSERMC11-16 and StudyNo.2020-910, respectively), and written informed consent was obtained from all subjects.

Note that full information on the approval of the study protocol must also be provided in the manuscript.

Field-specific reporting

Please select the one below that is the best fit for your research. If you are not sure, read the appropriate sections before making your selection.

☐ Life sciences ☒ Behavioural & social sciences ☐ Ecological, evolutionary & environmental sciences

For a reference copy of the document with all sections, see nature.com/documents/nr-reporting-summary-flat.pdf

Behavioural & social sciences study design

All studies must disclose on these points even when the disclosure is negative.

Study description	We recorded iEEG data from presurgical epilepsy patients when they were navigating a virtual T-maze with pre-defined rewards.
Research sample	Participants were epilepsy patients with implanted electrodes for presurgical evaluation. Implantation sites were chosen solely on the basis of clinical criteria. We selected participants which had electrodes in the anterior insular cortex and hippocampus.
Sampling strategy	Our analysis is based on iEEG recorded from 13 participants. Each participant performed 34-43 trials . No statistical methods were used to pre-determine sample size. The sample size is larger or equal to those in previous publications.
Data collection	Participants navigated the virtual T-maze on a screen of a computer via button press on a keyboard. Only the participant and the researcher were present at data collection, neither was blinded to the experimental goals or the hypothesis or the study at that time.
Timing	We collected data from consecutive patients.
Data exclusions	Epochs with breaks for clinical purposes (e.g., taking medicine), missing rewards (i.e., running out of the reward zone without picking up the reward), and incorrect responses at the decision zone (i.e., turning into a wrong arm) were excluded. Epochs contaminated by artifacts such as epileptic activities were detected by visual inspection and excluded from further data analysis.
Non-participation	The study excluded patients who declined to participate in the experiment.
Randomization	For each participant, a new T-maze was set randomly. The left- and right-turn signs at the decision zone were counter-balanced.

Reporting for specific materials, systems and methods

We require information from authors about some types of materials, experimental systems and methods used in many studies. Here, indicate whether each material, system or method listed is relevant to your study. If you are not sure if a list item applies to your research, read the appropriate section before selecting a response.

Materials & experimental systems

n/a	Involved in the study
☒	☐ Antibodies
☒	☐ Eukaryotic cell lines
☒	☐ Palaeontology and archaeology
☒	☐ Animals and other organisms
☒	☐ Clinical data
☒	☐ Dual use research of concern
☒	☐ Plants

Methods

n/a	Involved in the study
☒	☐ ChIP-seq
☒	☐ Flow cytometry
☐	☒ MRI-based neuroimaging

Plants

Seed stocks: n/a

Novel plant genotypes: n/a

Authentication: n/a

Magnetic resonance imaging

Experimental design

Design type: Clinical protocol for structural scanning

Design specifications: We used structural scanning only for electrode contact localization

Behavioral performance measures: We did not record any behavioral measures as the structural scanning was used only for contact localization.

Acquisition

Imaging type(s): Structural MRI

Field strength: 3 Tesla

Sequence & imaging parameters: T1-weighted MRI

Area of acquisition: whole brain scanning

Diffusion MRI: ☐ Used ☒ Not used

Preprocessing

Preprocessing software: The exact location of each contact in the anterior insular cortex and hippocampus was defined via visual inspection in the local space of each subject using FSLeyes.

Normalization: Data were not normalized.

Normalization template: Data were not normalized.

Noise and artifact removal: No noise or artifact removal was used.

Volume censoring: No volume censoring was performed.

Statistical modeling & inference

Model type and settings: The structural scanning was used for electrode contact localization.

Effect(s) tested: No effects were tested.

Specify type of analysis: ☐ Whole brain ☒ ROI-based ☐ Both

Anatomical location(s): The anterior insular cortex and hippocampus.

Statistic type for inference (See Eklund et al. 2016): We did not run any statistical test on MRI data.

Correction: We did not apply statistical corrections.

Models & analysis

n/a	Involved in the study
☒	☐ Functional and/or effective connectivity
☒	☐ Graph analysis
☒	☐ Multivariate modeling or predictive analysis
