# Supplementary material for: Phase-tuned modulation during reward expectancy in human anterior insular cortex
Source: Commun Biol. 2026 Jun 16;9:1148. doi: 10.1038/s42003-026-10427-1 (PMC13521940; doi:10.1038/s42003-026-10427-1)
Supplement: Supplementary file 3 [file 42003_2026_10427_MOESM3_ESM.pdf]

nature portfolio

Peer Review File

Phase-tuned modulation during reward expectancy in human anterior insular cortex

Corresponding Author: Dr Linglin Yang

This file contains all reviewer reports in order by version, followed by all author rebuttals in order by version.

Version 0:

Reviewer comments:

Reviewer #1

(Remarks to the Author)
The authors analyzed ECoG data obtained while participants navigated a virtual T-Maze. The authors focused on the pre-reward window to establish a neural marker of reward expectation in the anterior insula. The chosen analysis methods are robust and adequate for the established aim. I enjoyed reading the manuscript, especially the mechanistic approach to uncover reward expectation related neural mechanisms. The authors use a novel approach to analyze neural correlates of reward expectation. I have some major and minor points that would improve the manuscript.

Introduction:

- the manuscript does not focus on mental health disorders but rather a general and important reward mechanism, thus it is a bit confusing for the reader that the introduction starts with deficits in reward expectation and their importance in psychiatric disorders. In my opinion last part of the introduction, which states the research gap, is very compelling and highlights the novelty of the study

- could you add one or two sentences on the utility of phase precession for reward encoding specifically?

results:

- I am a bit confused about the RSA contrasts. Could you define the contrasts chosen in greater detail? I naively thought the best contrast would be a reward vs. no reward trial to get a reward specific brain pattern, but as far as I understand the observer is rewarded in every round of the maze. I would suggest to add one or two sentences to the results section explaining the T-Maze (what is the goal of that maze? Do participants always get a reward?). Currently, it's a bit difficult to comment on the RSA contrasts without this crucial information.

- the RBP pre-activation and PAC results are very interesting, I wonder if you could support your findings by including time delays. Is there a fixed time delay between RBP and PAC within patients, meaning patients with earlier RBPs also show a earlier PAC modulation?

- related to my earlier comment on RBP pre-activation and PAC: I am missing a mechanistic link between RBP pre and the phase precession effect and the response times. I would assume that PPLE depends on the strength of the pre RBP on a trial level, meaning that only a strong reward expectation will lead to behavioural change. This would make the link between expectation, behaviour and neural findings even stronger.

Methods:

- could you elaborate on the approach used to compare neural patterns (l. 609 ff), specifically the linear regression model (if you used a regression)

Minor points:

- I suggest checking the manuscript for spelling mistakes and sentence logic (substance addiction instead of substance

addition, l. 51, l. 161 revels instead of reveals, ll. 444 - 446)

- unfortunately no open access analysis code available to review

Reviewer #2

(Remarks to the Author)
The manuscript "Phase-tuned modulation during reward expectancy in human anterior insular cortex" by Yang et al. investigates the electrophysiological mechanisms underlying reward expectancy in the human anterior insular cortex (AIC) using intracranial EEG recordings from epilepsy patients performing a virtual T-maze task. The authors report three main findings: 1) pre-activation of reward brain patterns (RBPs) in AIC prior to reward onset, 2) enhanced theta–gamma phase amplitude coupling during reward expectancy that follows the RBP pre-activation, and 3) a trial-by-trial shift of gamma activity toward earlier theta phases across repeated approaches to the same reward, which the authors term a phase-precession-like effect (PPLE). They further show that PPLE strength correlates with behavioral improvements in response time.

Overall, the study is conceptually and methodologically interesting, and leverages iEEG to investigate the temporal dynamics of deep cortical structures. However, several conceptual, methodological, and inferential issues limit the strength of the conclusions in the current form of the manuscript. Some points of discussion appear to over-interpret the evidence, and certain analysis choices require a clearer justification or additional controls to rule out alternative explanations.

Major comments:
While the authors integrate RBPs, PAC, and PPLE into a coherent framework, several arguments overstate the specificity of these effects to reward expectancy. Given that reward value and timing are fixed, and that the reported neural and behavioral measures co-vary with action proximity and time on task, alternative interpretations in terms of general anticipatory, salience-related, or practice-related processes cannot be fully excluded by the current analyses. A more cautious general framing of the findings as reflecting anticipatory task states during reward approach, rather than reward expectancy per se, would better align the Discussion with the evidence presented.

The use of the hippocampus as a control region is helpful to demonstrate regional differences between AIC and medial temporal lobe signals. However, the absence of comparable effects in the hippocampus should be interpreted with caution. The hippocampus is itself strongly implicated in spatial navigation, temporal coding, and theta-gamma coupling in other task contexts, and null effects here may reflect task demands, electrode coverage, or signal characteristics rather than a true functional dissociation. As such, the hippocampal comparison supports regional differentiation but does not by itself establish the specificity of reward expectancy of the AIC findings. Explicitly acknowledging this limitation in the discussion would help setting the scope of the conclusions.

RBPs are operationalized as neural patterns showing higher representational similarity between reward epochs (across locations) than between reward and non-reward epochs (reward–reward > reward–non-reward). These RBP estimates are subsequently used as a reference template to identify time windows preceding reward onset that exhibit increased similarity (“pre-activation”). The approach is technically careful, and the authors implement several important controls, including for spatial location (central vs lateral arms) and for temporal autocorrelation (excluding pre- and post-reward activity from the same trial). Moreover, RBP pre-activation is shown stronger for pre-ERZ than for pre-EDZ periods (Fig. 2D, S1), supporting contextual specificity relative to decision-related processing. However, how can we be certain that no other processes which are highly likely present (at pre-ERZ and post-ERZ periods) and which definitely have an influence on AI (and hippocampal) activity, like attention, salience, or motor preparation/execution are not being captured by that definition?
More specifically, the definition of RBPs relies on a post-ERZ contrast and subsequent pre-ERZ pre-activation, but ERZ is tightly coupled to an imminent button press (PR), raising the possibility that the identified RBPs partially reflect response urgency or motor preparation rather than a reward-specific representation. Since the data includes already PR events (and corresponding iEEG epochs), the authors could compute PR–PR similarity and compare ERZ–ERZ similarity against ERZ–PR (and/or PR–EDZ) similarity using the same analysis pipeline (multilevel regression and cluster-based permutation). Demonstrating that ERZ-defined RBPs do not simply recapitulate PR-locked patterns (i.e., limited overlap in significant time windows, reduced ERZ–PR similarity relative to ERZ–ERZ) would substantially strengthen the interpretation that RBPs are reward-specific rather than reflecting generic motor- (i.e., keypress-) related dynamics.

The main PAC effect is estimated as a difference between pre-ERZ and post-ERZ periods, witha significant theta-gamma PAC cluster in AIC that is stronger before reward onset. While this is an interesting result, the reliance on a pre–post contrast introduces ambiguity regarding whether the effect reflects a selective enhancement during reward expectancy versus a relative reduction/disruption of coupling after ERZ due to sensory transients, motor execution, or task-completion dynamics. The inclusion of a randomly sampled baseline helps, but the baseline segments are not matched for navigation state or motor readiness, limiting the ability to attribute the effect to expectancy rather than to differences in behavioral state across pre vs post epochs. A stronger test would be to compare pre-ERZ PAC directly against pre-EDZ or pre-PR periods (anticipatory epochs with different cognitive demands), or examine the time-resolved PAC dynamics within the pre-ERZ interval to assess whether coupling is specifically expressed during expectancy versus ramping with proximity to action/reward onset.

The observed association between RBP pre-activation and theta-gamma PAC is intriguing and makes the authors suggest a link between representational and oscillatory processes in AIC. However, this relationship is assessed via correlations

across contacts and by inspecting the temporal ordering of those processes. As such, inferences about functional coupling at the trial level are limited, since cross-contact correlations could reflect variance related to other factors (e.g., signal quality or electrode placement) rather than a direct mechanistic relationship, and of course the finding that one event precedes another does not imply that the first drives the second. The authors should either use more cautious language or demonstratd trial-wise associations within contacts to strengthen the interpretation that PAC specifically coordinates or refines reward-related representations.

Significant PPLE is observed in only a subset of AIC contacts (9/25), indicating spatial heterogeneity in the effect. While such sparsity is expected in intracranial recordings, it limits the strength of region-level claims and makes it difficult to infer a systematic reward-expectancy mechanism without additional controls. Moreover, in terms of the "functional relevance of PPLE", both PPLE strength and behavioral performance evolve monotonically across navigation rounds, raising the possibility that their association reflects a general time-on-task or practice effect rather than reward-specific expectancy learning. Demonstrating that PPLE predicts behavioral performance beyond the effect of round number, or adopting a more cautious framing of PPLE as reflecting dynamic phase coordination during task engagement, would better align the interpretation with the evidence presented.

Minor:
Although this does not detract from the presence of theta–gamma coupling, demonstrating that the PAC effect persists after accounting for gamma power fluctuations would further strengthen the interpretation that coupling reflects coordinated oscillatory dynamics rather than amplitude-driven effects. I ention this because PAC metrics are sensitive to changes in high-frequency amplitude, and the manuscript reports increased gamma power during epochs associated with reward approach.

Figure 1 (perhaps 1B and 1D) could be adjusted to reflect the events of interest ERZ, EDZ, and PR.

There are typos here and there across the manuscript (e.g., “To test this hypothesise” in page 7) that should be corrected.

Version 1:

Reviewer comments:

Reviewer #1

(Remarks to the Author)
The authors have addressed all of my previous comments and included additional analyses and figures that significantly strengthen the manuscript and offer important mechanistic insights into the reported effects. I have only a few minor comments regarding the revised manuscript.

R1.5

The correlation between RBP and reaction times appears quite strong, whereas there is no significant correlation with PAC. This is an interesting observation; however, I would refrain from interpreting this as evidence against a simple linear cascade in the absence of causal manipulation of the temporal sequence. Given that PAC estimation is generally more sensitive to methodological choices and may exhibit higher variability than power-based measures (see van Driel et al., 2015), it is possible that detecting PAC–behavior relationships requires greater statistical power (lines 585–590, page 17).

van Driel, J., Cox, R., & Cohen, M. (2015). Phase-clustering bias in phase–amplitude cross-frequency coupling and its removal. Journal of Neuroscience Methods, 254, 60-72. https://doi.org/10.1016/j.jneumeth.2015.07.014

R1.6

I am still unsure about the multilevel linear regression model. Could the authors please specify the dependent variable and the predictors included in the model? It would also be helpful to include the regression specification (e.g., model syntax), along with the beta coefficients and the variance explained (R^2).

Thank you for providing the code and data. I did not run the scripts but briefly reviewed the Python code. I was unable to locate code related to the statistical analyses (in particular, the regression model mentioned above). Could the authors provide the code used to run the statistical analysis?

I congratulate the authors on a well-designed study, the novel dataset, and the thorough revision of the manuscript.

Reviewer #2

(Remarks to the Author)
I appreciate the time and effort that the authors spent on this revision. I think the manuscript has improved substantially, the additional analyses help support their arguments and I appreciate the newly included cautionary statements which help contextualize their interpretations with respect to the limitations of the study. The authors have addressed my comments and I recommend the manuscript for publication.

Open Access This Peer Review File is licensed under a Creative Commons Attribution 4.0 International License, which permits use, sharing, adaptation, distribution and reproduction in any medium or format, as long as you give appropriate credit to the original author(s) and the source, provide a link to the Creative Commons license, and indicate if changes were made.
In cases where reviewers are anonymous, credit should be given to 'Anonymous Referee' and the source.
The images or other third party material in this Peer Review File are included in the article's Creative Commons license, unless indicated otherwise in a credit line to the material. If material is not included in the article's Creative Commons license and your intended use is not permitted by statutory regulation or exceeds the permitted use, you will need to obtain permission directly from the copyright holder.
To view a copy of this license, visit https://creativecommons.org/licenses/by/4.0/

Point-by-point response

Referee #1 (Remarks to the Author):

R1.1. The manuscript does not focus on mental health disorders but rather a general and important reward mechanism, thus it is a bit confusing for the reader that the introduction starts with deficits in reward expectation and their importance in psychiatric disorders.

Response:

Thank you for your thoughtful review and constructive suggestions regarding the manuscript's focus and clarity. We agree that starting the introduction with deficits in psychiatric disorders could distract from the study's primary focus on general reward mechanisms. Following your suggestion, we have revised the opening of the introduction to more directly highlight the fundamental role of reward expectation in adaptive behavior.

Manuscript Revisions:

We have revised the Introduction section (lines 79-81 on page 3) accordingly. It now reads: "*Many daily behaviors of humans are driven by the expectancy of rewards, whether material or spiritual*[1,2]. *Reward expectancy has been proposed to boost behavioral performance*[2], *particularly when the expected reward is ultimately obtained*[3,4]."

R1.2. In my opinion last part of the introduction, which states the research gap, is very compelling and highlights the novelty of the study. Could you add one or two sentences on the utility of phase precession for reward encoding specifically?

Response:

We appreciate your constructive suggestion. This addition would strengthen the theoretical background linking oscillatory dynamics to reward processing.

Manuscript Revisions:

We have revised the Introduction section (lines 124-128 on page 4) accordingly. It now reads: "*Notably, phase precession has been implicated in reward-related spatial encoding, potentially facilitating the precise coordination of anticipation and action. Evidence from rodent studies have demonstrated this phenomenon in salience network nodes (e.g., the ventral striatum and lateral septum) during reward expectancy, where it selectively tracks the location of rewards*[41-44]."

R1.3. I am a bit confused about the RSA contrasts. Could you define the contrasts chosen in greater detail? I naively thought the best contrast would be a reward vs. no reward trial to get a reward specific brain pattern, but as far as I understand the observer is rewarded in every round of the maze. I would suggest to add one or two sentences to the results section explaining the T-Maze (what is the goal of that maze? Do participants always get a reward?). Currently, it's a bit difficult to comment on the RSA contrasts without this crucial information.

Response:

Thank you for raising this important point regarding the RSA contrasts. We agree that a direct comparison between reward and no-reward trials would be conceptually valuable. However, in the present task design,

rewards were delivered in all 469 ERZ (entering reward zone) events on the central corridor and in nearly all (459/464) ERZ events on the lateral arms. The very limited number of unrewarded trials precluded a statistically meaningful comparison. We now acknowledge the limitation in the revised manuscript.

We therefore selected the EDZ (entering decision zone) event as the primary contrast against the ERZ event on the central corridor for methodological considerations. First, the trial counts for EDZ (32.23 ± 2.39 [mean ± SD]) and ERZ events (32.08 ± 2.40) were closely matched, ensuring statistical balance. Second, both events involve comparable cognitive components (e.g., visual stimulus processing, spatial anticipation, and action preparation) while occurring in distinct spatial locations. This design helps isolate reward-specific encoding from general task-related activity by minimizing potential confounds related to visual input, motor preparation, and spatial context. In the original manuscript, we provided a detailed description of the T-maze paradigm and the rationale for this contrast in the Methods section (lines 1239-1243 on page 27) to ensure our approach is clear: "To minimize the influence of learning biases, we specifically focused on the reward zone in the central corridor, where participants' expectations for receiving a reward were consistently fulfilled (i.e., the reward would certainly appear). Another advantage is ensuring the balance among the trial numbers under the three conditions included in the analysis. Nevertheless, ERZ events occurred on the lateral roads were also extracted to identify the reward-specific brain patterns (RBPs)."

We apologize for the confusion caused by the unclear description of the T-Maze and have now add a clearer description in the Methods and Results section.

Manuscript Revisions:

We have revised the Results section (lines 150-162 on page 5). It now reads: "*Participants navigated a virtual T-maze to collect rewards while iEEG activity was recorded (Figure 1B-D). During each navigation round, they first entered the reward zone on the central corridor (where a reward was always delivered), and then the reward zone on a lateral arm (where reward was presented in 98.90 ± 3.28% [mean ± SD] trials following correct responses at the decision zone; see Methods). To minimize the influence of spatial heterogeneity, we focused on iEEG activity time-locked to three key events occurring on the central corridor: entering the reward zone (ERZ, experimental condition), picking up the reward (PR, control condition), and entering the decision zone (EDZ, control condition). The number of trials included for each event was matched (32.08 ± 2.40 [mean ± SD] for ERZ, 32.23 ± 2.42 for PR, and 32.23 ± 2.39 for EDZ; $F_{(2, 24)} = 0.13$, $p = 0.79$, one-way ANOVA; Figure 1E and Supplementary Table 1). Additionally, ERZ events occurring on lateral arms (13.23 ± 3.42 for left arm, and 13.46 ± 3.93 for right arm) were included to identify reward-specific brain patterns (RBPs) while accounting for potential spatial confounds. For each ERZ event on the central corridor, the response time (RT) for picking up the reward was calculated for further analysis (Figure 1F; see Methods).*"

In the Discussion section (lines 886-988 on pages 21-22), we have added the limitations: "*Moreover, the extremely limited number of unrewarded trials prevented a statistically meaningful comparison between reward and no-reward conditions. To mitigate this limitation, we employed trial-matched EDZ periods as a control condition for ERZ, effectively accounting for confounding factors such as visual stimulus processing, spatial anticipation, and action preparation. This approach enabled maximal control for general task-related and motor-preparatory activity, thereby isolating neural signals specific to reward expectancy.*"

In the Methods section (lines 1162-1192 on page 26), it now reads: "*Participants initiated each navigation round in the designed starting zone, advancing along the central corridor that contained a guaranteed-reward zone (0.15 € coin). Upon reaching the designated decision zone, a wall-mounted directional cue (circle for left-turn, and triangle for right-turn) indicated the correct lateral-arm path leading to an additional reward (Figure 1C and D). If the incorrect lateral arm was chosen, no reward was available on that route. Visible coins*

required a prompt "Enter" key press for collection before automatic disappearance upon reward-zone exit. Following reward collection, participants returned to the starting zone along the lateral arm to begin subsequent navigation round."

R1.4. The RBP pre-activation and PAC results are very interesting, I wonder if you could support your findings by including time delays. Is there a fixed time delay between RBP and PAC within patients, meaning patients with earlier RBPs also show a earlier PAC modulation?

Response:

Thank you for this insightful suggestion. We agree that investigating whether there exists a fixed temporal relationship between RBP pre-activation and PAC modulation across individuals is important. To address this question, we performed systematic analyses at multiple levels (trials, contacts, and participants) as presented in the revised manuscript. Across all trials and contacts, peak gamma activity consistently occurred after RBP pre-activation ($p < 0.001$; Wilcoxon signed-rank test). However, the magnitude of the time delay was highly variable showing a high dispersed distribution (coefficient of variation [CV] = 85.77%) across all trials and contacts. We also investigate the temporal order effect at trial level by comparing the time onset of RBP pre-activation and peak PAC across trials within each contact. The same temporal order was found in 24 out of 25 contacts ($ps < 0.001$), which was significantly above chance level ($p = 1.42\times10^{-30}$; Binomial test). It indicates that the RBP pre-activation reliably precedes PAC enhancement at trial level. Again, we further checked the magnitude of the time delay and found high dispersed distribution of the time lag in all contacts (CVs ≥ 62%). We applied the same analysis at contact and participant levels and found the same temporal order effect but a moderate dispersed distribution of the time lag between the time onset of RBP pre-activation and peak PAC ($CV_{contact}$ = 23.0%; $CV_{participant}$=18.8%). These results suggest that while the sequential order is constant (RBP pre-activation → PAC effect), the absolute temporal interval between these two events is not fixed but rather exhibits considerable flexibility. They lead us to a more nuanced interpretation: the AIC exhibits a robust sequential engagement of representational (RBP) and oscillatory (PAC) processes during reward expectancy, but the precise temporal coupling between them is not governed by a fixed delay. We thank the reviewer for prompting this deeper characterization of the RBP-PAC temporal dynamics, which we believe substantially strengthens our understanding of the sequential relations between RBP pre-activation and PAC effects in the AIC during reward expectancy.

Manuscript Revisions:

In the Abstract section (lines 31-32 on page 2), we revised as follows: "*This pre-activation was followed by phase-amplitude coupling (PAC) between theta oscillations and gamma activity, with coupling strength positively correlated with the pre-activation level across contacts.*"

In the Results section (lines 399-455 on pages 12-13), we have updated the text to present systematic analyses across multiple levels (trials, contacts, and participants). "*Additionally, we examined the temporal relationship between the peak theta-gamma PAC effect and the onset of RBP pre-activation. The peak PAC effect for each pre-ERZ period was defined as the emergence of peak gamma activity (75-110 Hz) with the maximal amplitude at the preferred PAC phase (5-7 Hz; see Methods). Across all trials and contacts, the peak gamma activity occurred markedly later than the initial RBP pre-activation (-1.00 ± 0.02 seconds [mean ± SEM] vs. -1.70 ± 9.7×10^{-3} seconds, $p < 0.001$; Wilcoxon matched-pairs signed rank test; Figure 3G), with a mean time lag of 0.73 ± 0.02 seconds. The coefficient of variation (CV, 85.77%) indicated a high dispersed distribution of the time lag. In individual contacts, the peak PAC effect was significantly delayed relative to RBP pre-activation*

across trials in 24 out of 25 contacts (ps < 0.001), with no significant difference in the remaining contact (p = 0.697; Supplementary Figure 7). We again investigated the time lag within each contact and found high dispersed distributions of the time lag in all contacts (CVs ≥ 62%; Supplementary Table 2). We then averaged the timing of both events across trials for each contact and compared them at the contact level (Supplementary Table 2 and Supplementary Figure 8A). Consistent with the above results, the peak PAC effect occurred significantly later than RBP pre-activation across the 25 contacts (-1.00 ± 0.004 seconds vs. -1.72 ± 0.004 seconds, p < 0.001; Supplementary Figure 8B), with delays ranging from 0.04 to 0.92 seconds (CV = 23%), suggesting a moderate dispersed distribution of the time lag across contacts. To preclude potential bias introduced by uneven contact numbers across participants, we performed the analysis at the participant level by averaging across contacts within each participant. The peak PAC effect (-0.97 ± 0.08 seconds [mean ± SD]; n = 9) again occurred significantly later than the initial RBP pre-activation (-1.70 ± 0.09 seconds; p = 0.0039; Supplementary Figure 8C). The time lags at the participant level ranged from 0.45 to 0.90 seconds (CV = 18.8%), further indicating the absence of a fixed temporal delay between the onset of RBP pre-activation and the peak of theta-gamma oscillatory coordination. These findings demonstrate a temporal sequence in which RBP pre-activation consistently precedes before the peak of theta-gamma oscillatory coordination during the pre-ERZ period. Furthermore, this temporal sequence pattern was robust across trials, contacts, and participants. However, the interval between these two events is highly variable, suggesting that the precise timing of their interaction is not fixed, but may instead be modulated by ongoing cognitive demands."

In the Discussion section, we have explicitly acknowledged the heterogeneity across contacts and adopted more circumspect language.

> At lines 682-683 on page 18, "*Subsequent observation revealed enhanced theta-gamma PAC, the peak of which occurred following the rise of RBP pre-activation in most recording contacts.*"
>
> At lines 835-863 on pages 20-21, "*Collectively, these findings indicate a potential sequence in which preactivated RBPs provide the representational content ("what"), and subsequent theta-gamma PAC in the AIC contributes to the temporal structure ("when" and "how") of reward expectancy.*"
>
> At lines 883-989 on pages 21-22, "*Taken together, our population-level findings reveal a temporally ordered and correlated interplay between representational pre-activation and oscillatory coordination within human AIC for reward expectancy. However, it should be noted that the observed correlation and temporal sequence between PAC and RBP pre-activation exhibited heterogeneity across individual recording contacts. Moreover, the extremely limited number of unrewarded trials prevented a statistically meaningful comparison between reward and no-reward conditions. To mitigate this limitation, we employed trial-matched EDZ periods as a control condition for ERZ, effectively accounting for confounding factors such as visual stimulus processing, spatial anticipation, and action preparation. This approach enabled maximal control for general task-related and motor-preparatory activity, thereby isolating neural signals specific to reward expectancy. Future studies incorporating more extensive electrode coverage and causal manipulations will be essential to clarify the consistency and directionality of this relationship.*"

In the Supplementary Information, we added two new supplementary figures and a supplementary table to illustrate the temporal sequence of RBP pre-activation and PAC at trial (Supplementary Figure 7), contacts and participant level (Supplementary Figure 8 and Supplementary Table 2).

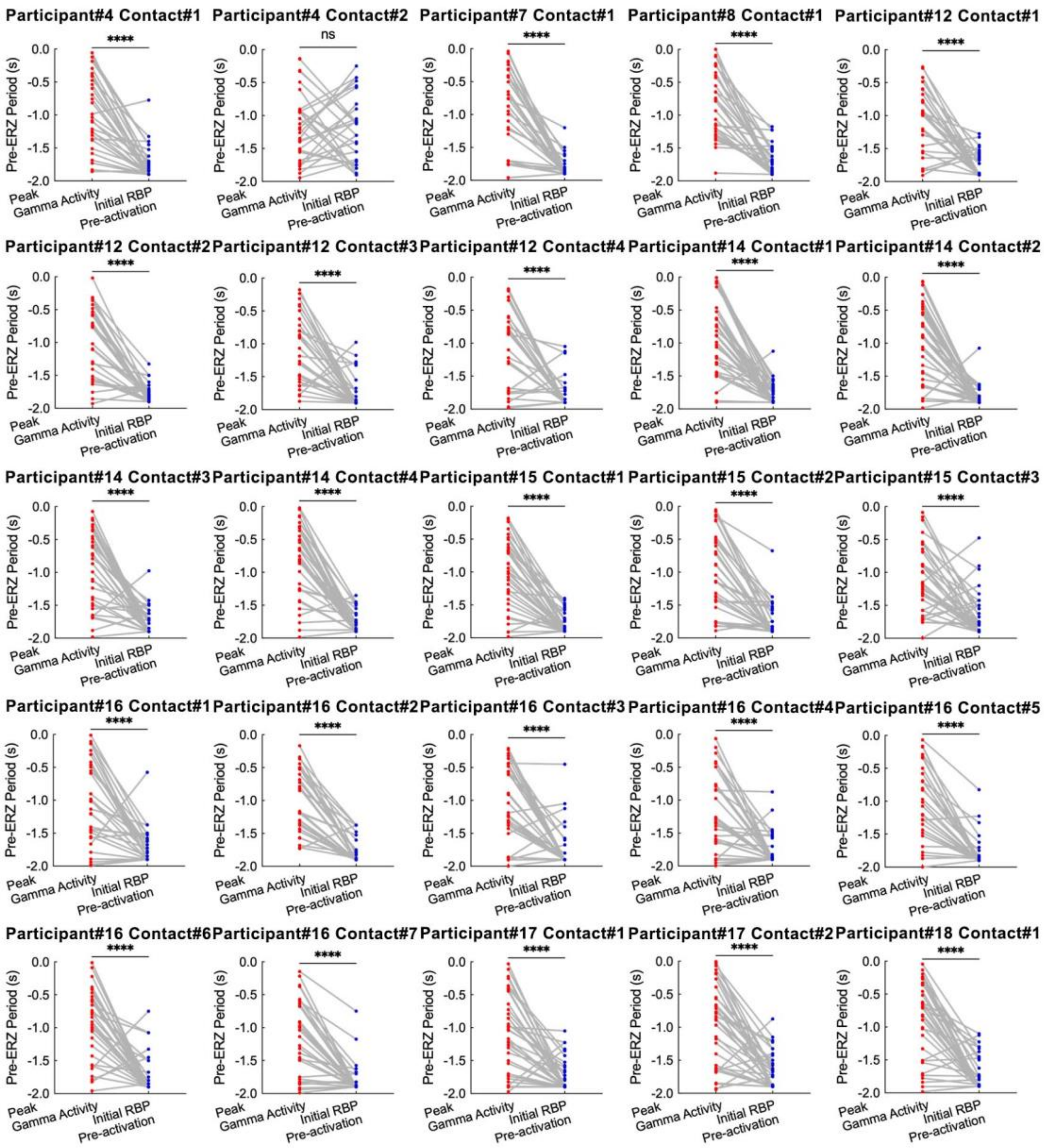

Supplementary Figure 7. Temporal sequence of RBP pre-activation and PAC effect at the trial level. Related to Figure 3. *Trial-wise time points of peak gamma activity (red) and initial RBP pre-activation (blue) within each contact. Each panel represents one contact, and dots indicate individual trials. Y-axis shows time relative to pre-ERZ periods; time 0 represents reward onset. ****, $p < 0.001$; ns, not significant.*

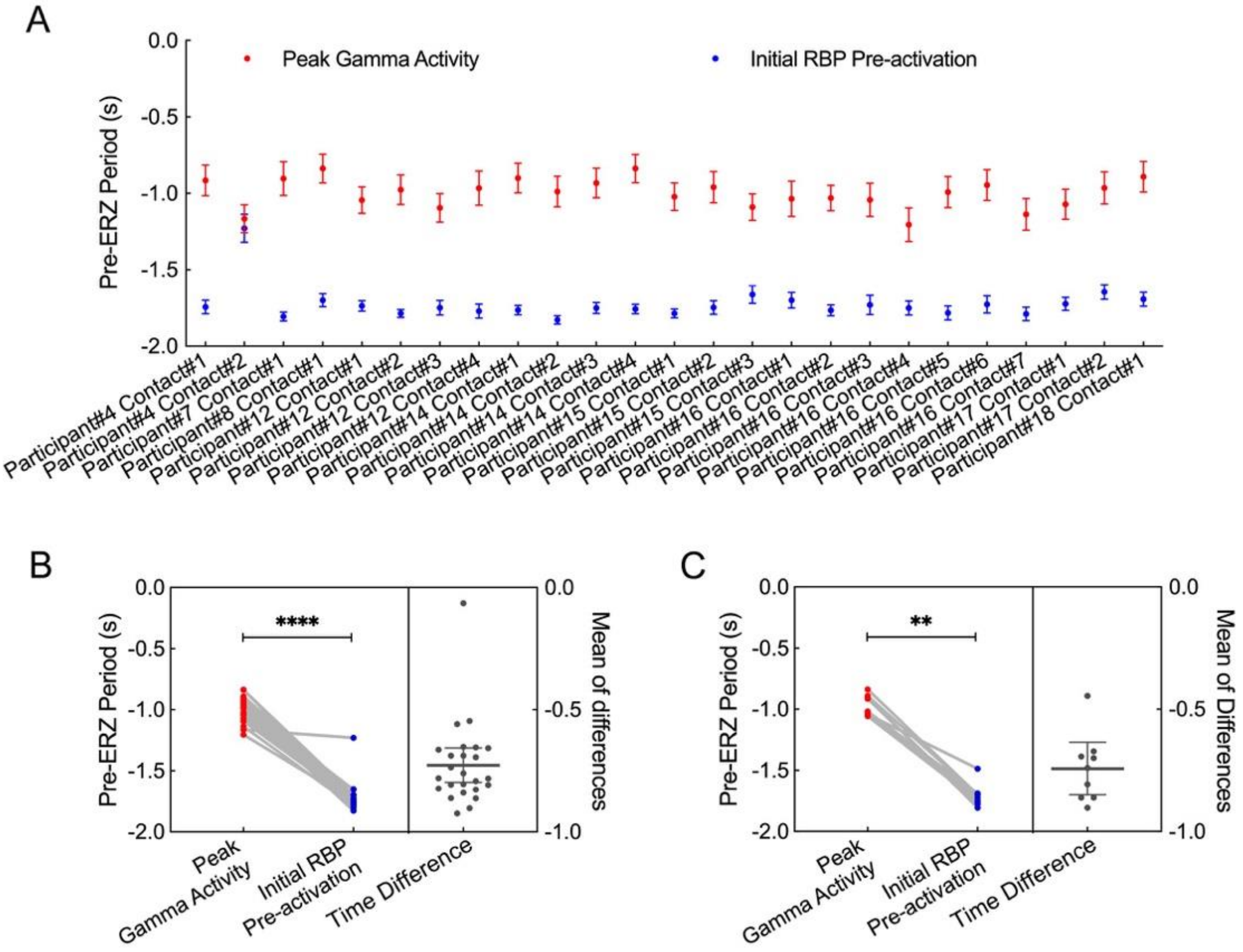

Supplementary Figure 8. Temporal sequence of RBP pre-activation and PAC effect at the contact and participant level. Related to Figure 3. (A) *The initial pre-activation of reward-specific brain patterns (RBPs, blue) precedes the peak gamma activity (red) at the preferred theta phase (derived from phase-amplitude coupling, PAC) in the 24 out of 25 contacts. Dots represent individual recording contacts, and error bars denote SEM.* ***(B)*** *Time difference between peak gamma activity and initial RBP pre-activation across contacts.* ***(C)*** *Time difference between peak gamma activity and initial RBP pre-activation across participants. Y axis indicates the time course relative to pre-ERZ periods, and time 0 indicates reward onset. **, $0.005 \leq p < 0.01$; ****, $p < 0.001$.*

Supplementary Table 2. Time difference between the initial pre-activation of reward-specific brain patterns and the peak gamma activity in individual contacts. Related to Figure 3.

Contact	ERZ Trials	Time of Initial Pre-activation		Time of Peak Gamma Activity		Time Lag	
		Mean ± SEM (s)	CV (%)	Mean ± SEM (s)	CV (%)	Mean ± SEM (s)	CV (%)
Participant#4 Contact#1	31	-1.74 ± 0.01	14.33	-0.92 ± 0.02	60.83	-0.83 ± 0.02	69.40
Participant#4 Contact#2	31	-1.23 ± 0.02	41.57	-1.19 ± 0.02	41.58	-0.04 ± 0.02	1875.09
Participant#7 Contact#1	30	-1.81 ± 0.01	9.01	-0.90 ± 0.02	67.15	-0.90 ± 0.02	67.94
Participant#8 Contact#1	29	-1.70 ± 0.01	13.63	-0.84 ± 0.02	60.60	-0.86 ± 0.02	63.29
Participant#12 Contact#1	30	-1.74 ± 0.01	11.20	-1.04 ± 0.02	45.73	-0.69 ± 0.02	82.13
Participant#12 Contact#2	30	-1.79 ± 0.00	8.08	-0.98 ± 0.02	54.39	-0.81 ± 0.02	66.57
Participant#12 Contact#3	30	-1.75 ± 0.01	15.10	-1.09 ± 0.02	46.99	-0.65 ± 0.02	91.63
Participant#12 Contact#4	30	-1.77 ± 0.01	14.05	-0.97 ± 0.02	63.86	-0.80 ± 0.02	89.77
Participant#14 Contact#1	34	-1.76 ± 0.01	10.12	-0.90 ± 0.02	63.96	-0.86 ± 0.02	70.71
Participant#14 Contact#2	34	-1.83 ± 0.00	8.91	-0.99 ± 0.02	60.83	-0.84 ± 0.02	79.36
Participant#14 Contact#3	34	-1.75 ± 0.01	12.26	-0.93 ± 0.02	61.40	-0.82 ± 0.02	81.42
Participant#14 Contact#4	34	-1.76 ± 0.01	10.14	-0.84 ± 0.02	67.62	-0.92 ± 0.02	66.45
Participant#15 Contact#1	34	-1.78 ± 0.00	9.51	-1.02 ± 0.02	52.33	-0.76 ± 0.02	69.49
Participant#15 Contact#2	34	-1.74 ± 0.01	15.00	-0.96 ± 0.02	60.64	-0.78 ± 0.02	70.07
Participant#15 Contact#3	34	-1.65 ± 0.01	20.98	-1.09 ± 0.02	46.81	-0.56 ± 0.02	112.00
Participant#16 Contact#1	30	-1.70 ± 0.01	16.22	-1.04 ± 0.02	61.44	-0.66 ± 0.02	102.90
Participant#16 Contact#2	30	-1.77 ± 0.01	11.34	-1.03 ± 0.02	44.77	-0.74 ± 0.02	62.00
Participant#16 Contact#3	30	-1.73 ± 0.01	19.78	-1.04 ± 0.02	56.55	-0.69 ± 0.02	96.97
Participant#16 Contact#4	30	-1.75 ± 0.01	14.63	-1.20 ± 0.02	50.60	-0.55 ± 0.02	118.64
Participant#16 Contact#5	30	-1.78 ± 0.01	14.05	-0.99 ± 0.02	56.70	-0.79 ± 0.02	70.00
Participant#16 Contact#6	30	-1.73 ± 0.01	17.75	-0.95 ± 0.02	57.74	-0.78 ± 0.02	89.01
Participant#16 Contact#7	30	-1.79 ± 0.01	13.95	-1.14 ± 0.02	50.26	-0.65 ± 0.02	82.56
Participant#17 Contact#1	34	-1.73 ± 0.01	14.79	-1.07 ± 0.02	55.37	-0.66 ± 0.02	99.63
Participant#17 Contact#2	34	-1.65 ± 0.01	16.64	-0.97 ± 0.02	61.70	-0.69 ± 0.02	95.54
Participant#18 Contact#1	34	-1.70 ± 0.01	16.09	-0.89 ± 0.02	66.45	-0.81 ± 0.02	81.04

Abbreviations: ERZ, entering the reward zone; SEM, standard error of the mean; CV, coefficient of variation.

R1.5. Related to my earlier comment on RBP pre-activation and PAC: I am missing a mechanistic link between RBP pre and the phase precession effect and the response times. I would assume that PPLE depends on the strength of the pre RBP on a trial level, meaning that only a strong reward expectation will lead to behavioural change. This would make the link between expectation, behaviour and neural findings even stronger.

Response:

Thank you for raising this essential point regarding the mechanistic link between RBP pre-activation, PPLE, and behavioral improvement. Your insightful suggestion prompted us to perform the direct correlation analyses you proposed, and the results have refined our interpretation.

Specifically, we examined the relationship between these metrics. PPLE strength was found to be independent of both average PAC strength ($r = -0.09$, $p = 0.67$) and average RBP pre-activation level ($r = -0.25$, $p = 0.24$) across contacts. At the participant level, behavioral improvement showed a strong negative correlation with average RBP pre-activation level ($r = -0.83$, $p = 0.006$), but no significant correlation with average PAC strength ($r = -0.22$, $p = 0.57$).

Together with our previously reported findings that RBP pre-activation correlates positively with PAC strength and consistently precedes it in time, and that the preferred PAC phase exhibits PPLE which correlates with behavioral improvement, these findings support an integrated "phase-tuned modulation" model. In this model, theta-gamma PAC serves as a critical intermediary linking representational content to behavioral outcomes through two distinct functional characteristics. First, PAC strength reflects the engagement of oscillatory dynamics following representational activation. The positive correlation between RBP pre-activation and PAC strength, together with their temporal sequence (RBP pre-activation → PAC), suggests that reward expectancy-related pre-activation may trigger or facilitate oscillatory coordination in the AIC. However, PAC strength itself does not directly predict behavior, indicating that it represents a permissive condition rather than the ultimate coding mechanism. Second, the preferred PAC phase provides temporally precise coding. The preferred phase exhibits PPLE, whose strength correlates with the magnitude of behavioral improvement. Crucially, PPLE strength is independent of both PAC strength and RBP pre-activation level, indicating that phase coding quality is a distinct property that operates separately from the magnitude of oscillatory engagement.

We have incorporated these new results and the refined mechanistic framework into the revised manuscript and sincerely appreciate your suggestion, which has significantly deepened our conclusions.

Manuscript Revisions:

In the Results section, we added the results of supplementary analysis.

At lines 538-544 on page 16, "*Subsequently, we examined whether PPLE strength correlated with either average PAC strength or average RBP pre-activation level across contacts in the AIC during the pre-ERZ period. PPLE strength was quantified as the absolute value of the circular-linear coefficient for each contact. The result showed that the PPLE strength was independent of both average PAC strength ($r = -0.09$, $p = 0.67$; Spearman's correlation) and average RBP pre-activation level ($r = -0.25$, $p = 0.24$; Supplementary Figure 13). These results indicate that PPLE, characterized by a progressive shift in PAC phase across rounds, represents a distinct phenomenon from both the enhanced theta-gamma PAC and the earlier RBP pre-activation.*"

At lines 585-590 on page 17, "*We first examined how behavioral improvement related to RBP pre-activation level and PAC strength. The result showed that the magnitude of behavioral improvement showed a negative correlation with the average level of RBP pre-activation across participants ($r = -0.83$, $p = 0.006$; Pearson's correlation), but no significant correlation with average PAC strength ($r = -0.22$, $p = 0.57$; Supplementary Figure 15A and B). These results indicate that reward-directed behavioral improvement is not supported by a simple linear cascade of representational pre-activation and cross-frequency coupling.*"

In the Discussion section (lines 687-789 on pages 18-19), we provided a more detailed description of the "phase-tuned modulation" model. "*Collectively, these results delineate a dynamic temporal structure that supports an integrated "phase-tuned modulation" model in which theta-gamma PAC serves as a critical intermediary linking representational content to behavioral outcomes through two functionally distinct characteristics (Figure 6). In this framework, PAC strength reflects the engagement of oscillatory dynamics following representational (RBP) pre-activation, while the preferred PAC phase provides temporally precise coding manifesting as PPLE that translates expectational states into behavioral optimization. This framework highlights phase-tuned oscillatory dynamics (e.g., PAC and PPLE) as candidate electrophysiological mechanisms through which the AIC facilitate reward expectancy.*"

In the Figure 6 (pages 19-20), we added panel C delineating the integrated "phase-tuned modulation" model.

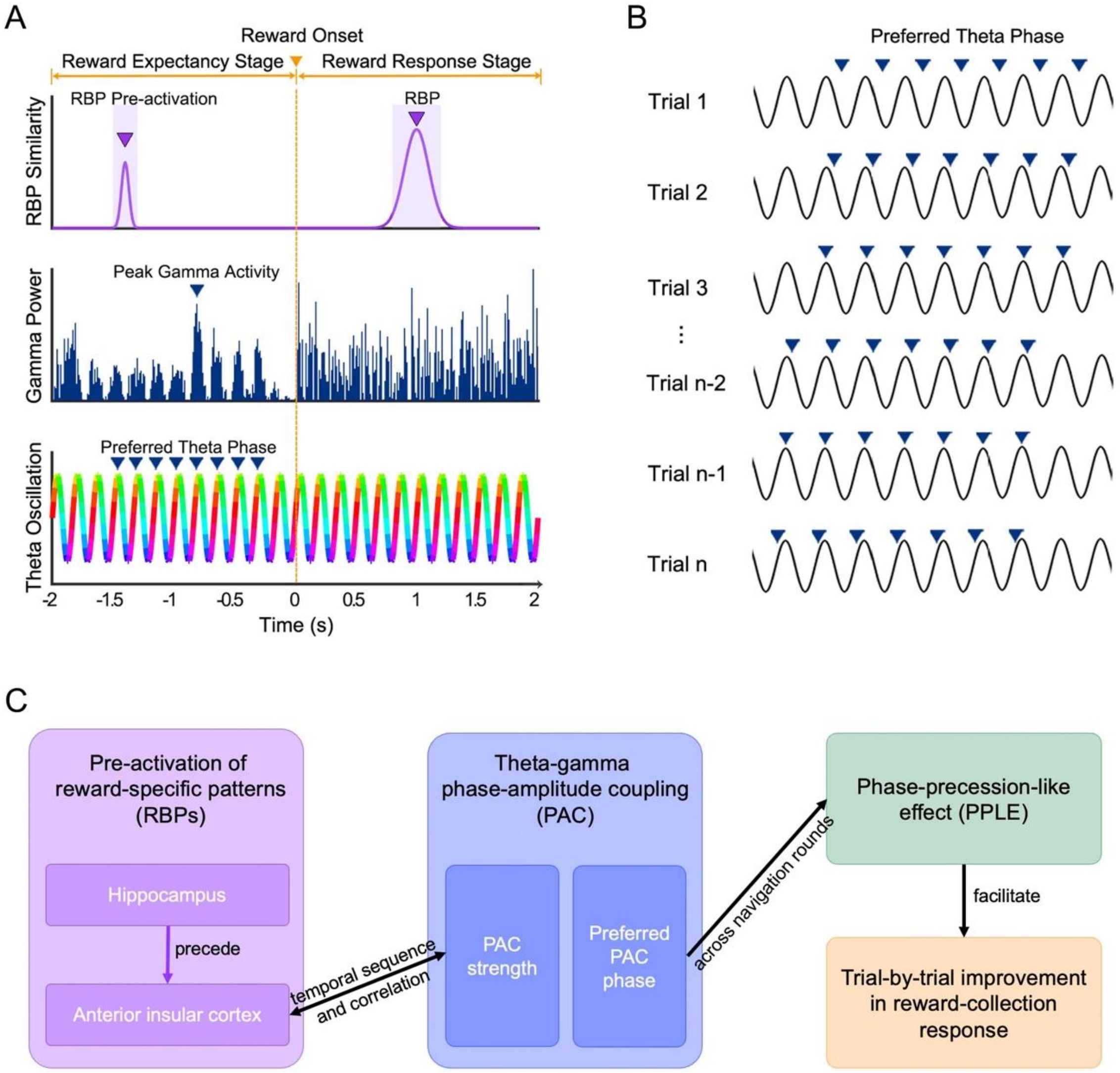

Figure 6. Dual phase-tuned modulation mechanisms underlying reward expectancy in the AIC. (A) *Schematic of single-trial neural dynamics. Top: The reward-specific brain pattern (RBP, purple trace) shows anticipatory pre-activation (shaded area) during the reward expectancy stage, peaking at the critical timepoint (purple triangle). Middle: Gamma-band power (blue bars) exhibits phase-tuned modulation by theta oscillations. Peak gamma activity (blue triangle), which is coupled to the preferred theta phase, emerges after the RBP pre-activation. Bottom: Theta oscillations with continuous phase encoding (0-2π gradient color map). Blue triangles mark the preferred phases of phase-amplitude coupling (PAC).* ***(B)*** *Trial-to-trial phase-precession-like effect (PPLE). Gamma activity are coupled with advanced theta phases (blue triangles) across trials. Individual trials demonstrate progressive phase advancement of theta-gamma PAC (blue triangles) across successive reward experiences.* ***(C)*** *PAC serves as a critical bridge in two senses: its strength links back to representational content (RBP pre-activation), reflecting whether oscillatory coordination is engaged; its phase links forward to behavioral outcomes via PPLE, determining how that coordination is temporally organized to support behavioral optimization.*

In the Supplementary Information, we added two new supplementary figures corresponding to additional analysis.

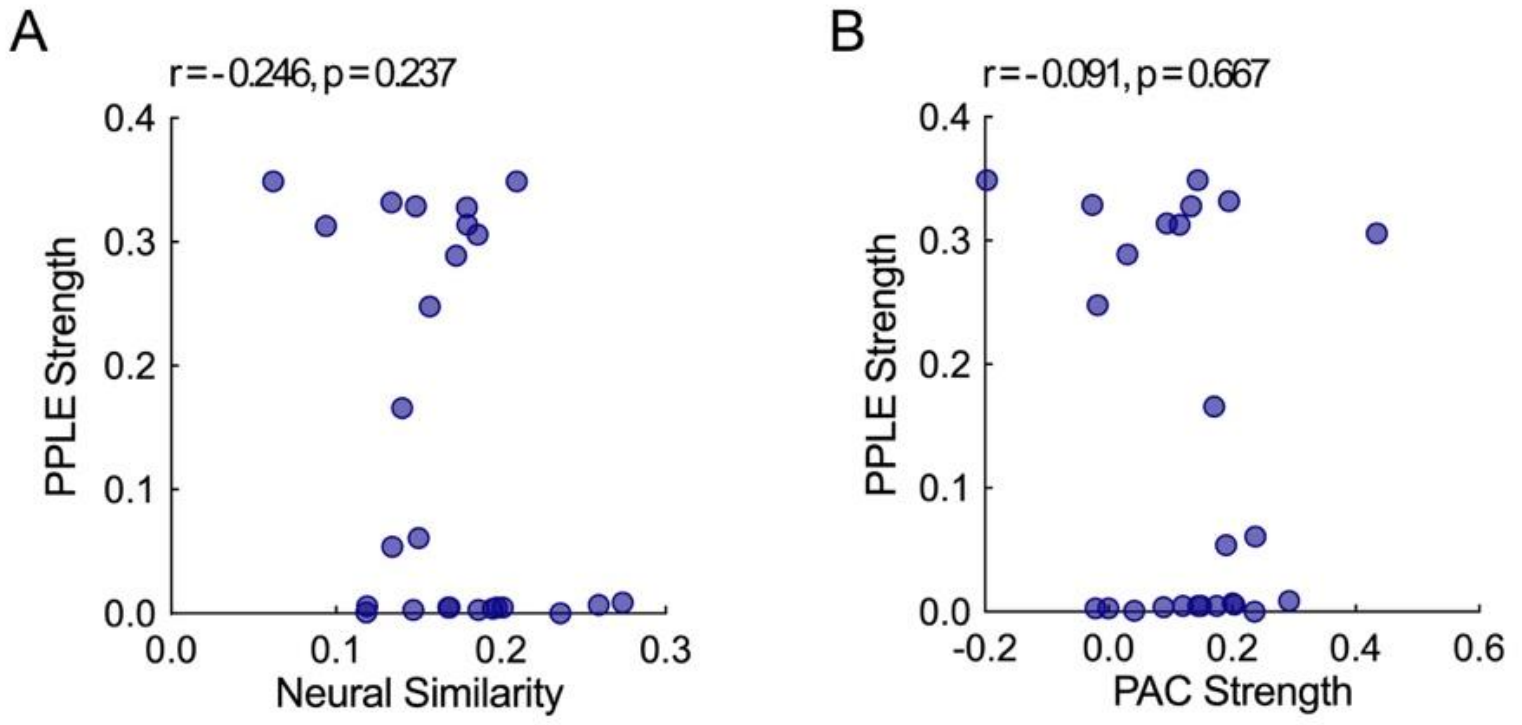

Supplementary Figure 13. The PPLE strength did not demonstrate a significant correlation with either RBP pre-activation level (A) or PAC strength (B). *Each blue sphere represents one recording contact.*

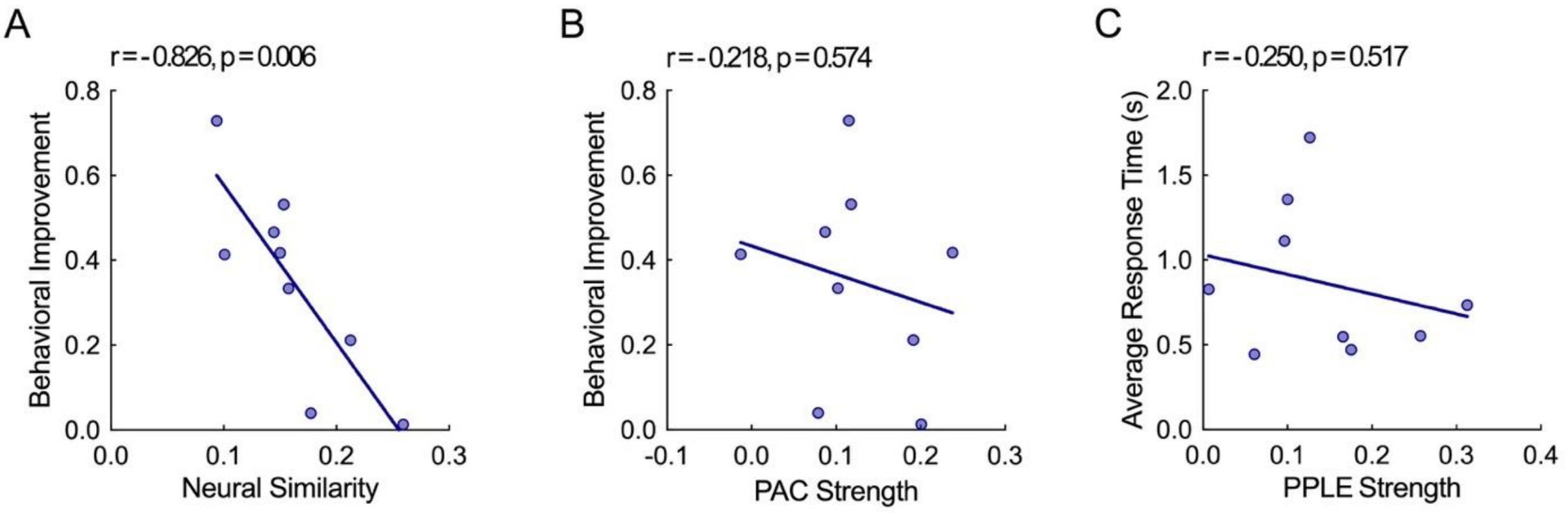

Supplementary Figure 15. Relationship between behavioral performance and identified neural signatures. Relation to Figure 5. (A) *Behavioral improvement demonstrates a strong negative correlation with average pre-activation level of reward-specific brain pattern (RBP).* ***(B)*** *No significant correlation was observed between behavioral improvement and the average strength of phase-amplitude coupling (PAC).* ***(C)*** *There was no correlation between the average response time and the strength of phase-precession-like effect (PPLE). Each blue sphere represents one recording contact.*

R1.6. could you elaborate on the approach used to compare neural patterns (l. 609 ff), specifically the linear regression model (if you used a regression)

Response:

Thank you for your question regarding the details of our neural pattern comparison and the use of linear regression. We have updated the Methods section to provide a clearer and more detailed description.

Manuscript Revisions:

In the Methods section, we have elaborated on the analysis pipeline.

At lines 1318-1324 on page 29, "*To isolate RBPs from general neural activity, we compared brain patterns between post-ERZ and post-EDZ periods. Using the same procedure, a similarity vector for each post-ERZ/post-EDZ pair ($RSA_{postERZ/postEDZ}$) was generated. Crucially, pairs from the same navigation round or same spatial location were excluded to control for potential confounds due to temporal proximity and location-specific effects. To assess the statistical difference between $RSA_{postERZ/postERZ}$ and $RSA_{postERZ/postEDZ}$, we conducted*

multi-level linear regression analysis accounting for variance at both the participant and contact levels for each time bin."

At lines 1371-1379 on page 30, we have further detailed the analysis of RBP pre-activation. "*To control for location-specific confounds, we compared brain patterns in each pre-ERZ time window (on the central corridor) with RBPs derived from post-ERZ periods (on the lateral roads) using Spearman's correlations. This generated a 1×73 similarity vector for each pre-ERZ period and each recording contact ($RSA_{preERZ/RBP}$; Figure 2C). As a control analysis, the same procedure was applied to pre-EDZ periods (i.e., from 2 seconds before EDZ event), yielding $RSA_{preEDZ/RBP}$. To avoid temporal autocorrelations, similarity values ($RSA_{preERZ/RBP}$ and $RSA_{preEDZ/RBP}$) from the same navigation round were excluded for further analysis. We then performed a statistical comparison between $RSA_{preERZ/RBP}$ and $RSA_{preEDZ/RBP}$ for each time window, using multi-level linear regression that considered participant- and contact-level variances.*"

R1.7. I suggest checking the manuscript for spelling mistakes and sentence logic (substance addiction instead of substance addition, l. 51, l. 161 revels instead of reveals, ll. 444 - 446)

Response:

We apologize for the spelling mistakes and thank you for your meticulous review and for identifying these language issues. We have carefully checked the entire manuscript for spelling, grammar, and logical coherence.

Manuscript Revisions:

The phrase containing "substance addition" (page 3) was removed during the broader revision of the introduction's opening, following your earlier suggestion; while "revels" has been corrected to "reveals" (line 244 on page 8).

We have also reviewed and refined the sentence logic throughout to ensure clarity and accuracy. For example, in the section of Discussion (lines 1011-1033 on pages 22-23), we revised as: "*During reward expectancy, peak gamma activity derived from PAC analysis was found to be systematically advanced towards earlier theta phases as rewards approached across trials, a phenomenon termed PPLE.*"

R1.8. unfortunately no open access analysis code available to review

Response:

Thank you for noting this. The analysis code for this study has now been deposited in the public repository figShare and is available for review (https://figshare.com/s/7d4ec0ac23271ca0549d). Please let us know if you encounter any issues accessing the repository or if further clarification regarding the code is needed.

Referee #2 (Remarks to the Author):

R2.1. While the authors integrate RBPs, PAC, and PPLE into a coherent framework, several arguments overstate the specificity of these effects to reward expectancy. Given that reward value and timing are fixed, and that the reported neural and behavioral measures co-vary with action proximity and time on task, alternative interpretations in terms of general anticipatory, salience-related, or practice-related processes cannot be fully excluded by the current analyses. A more cautious general framing of the findings as reflecting anticipatory task states during reward approach, rather than reward expectancy per se, would better align the Discussion with the evidence presented.

Response:

We sincerely thank you for raising this important point regarding the specificity of our findings. We fully agree that in a task with fixed reward timing, it is crucial to carefully distinguish reward-specific expectancy from more general processes related to task engagement, action proximity, or practice. The experimental design was meticulously crafted to address this challenge.

To eliminate the confounding effects, we introduced the EDZ (entering the decision zone) event as a tightly matched control for the ERZ (entering the reward zone) event during data analysis. These two events are highly comparable in their cognitive and motor demands (both involve visual stimulus processing, spatial anticipation, button preparation, and action execution), with the critical difference being that EDZ is decoupled from immediate reward delivery. This allows us to maximally control for general task-related and motor-preparatory activity, thereby isolating neural signals specific to reward expectancy.

This matched control logic is consistently applied across our key analyses:

1. Pre-activation for reward-specific brain patterns (RBPs): We directly compared $RSA_{postERZ/postERZ}$ with $RSA_{postERZ/postEDZ}$. The significantly higher similarity within postERZ pairs indicates neural patterns specific to the reward context. Moreover, these patterns were pre-activated before reward onset, by comparing $RSA_{preERZ/RBP}$ with $RSA_{preEDZ/RBP}$. This series of contrasts strongly suggests that RBP pre-activation reflect anticipation of the upcoming reward rather than general task preparation.
2. Theta-gamma phase-amplitude coupling (PAC): The enhanced PAC observed during pre-ERZ period was specific relative to baselines, pre-EDZ (which controls for visual, navigational, general anticipatory, and motor-preparatory components) and pre-PR period (which controls for reward itself). Therefore, this enhancement can be more confidently attributed to the cognitive process of reward expectancy.
3. Phase-Precession-Like Effect (PPLE): PPLE occurred specifically during pre-ERZ period, rather than pre-EDZ and pre-PR periods. Furthermore, PPLE strength correlated with the degree of trial-over-trial behavioral improvement rather than with average reaction time. Together, these findings suggest that the functional relevance of PPLE extends beyond simple time-on-task or practice effects, pointing to its specific role in behavioral optimization related to reward expectancy.

We have revised the Discussion to adopt more cautious and precise language. In line with this point, we have conducted additional control analysis and revised the Results section according to R2.4, as well as revised the Methods section by adding more details of the practical session of the experiment according to R2.7.

Manuscript Revisions:

In the Discussion section

At lines 1085-1092 on page 24, we revised the manuscript as follows: "*We observed that the strength of PPLE was correlated with the magnitude of trial-by-trial behavioral improvement. This suggests that PPLE reflects a precise phase-tuned timing mechanism engaged during reward expectancy. Future studies with larger*

sample size and greater statistical power are needed to determine whether the PPLE specifically tracks reward-specific processes or is related to general practice effects. Nevertheless, the relationship between PPLE strength and the progressive optimization of responses towards upcoming rewards highlights a potential role for phase-tuned oscillatory dynamics in optimizing behavioral performances[1,2,4]*.*"

At lines 1099-1102 on page 24, we outline key directions for further studies. "*Future research should employ multidimensional reward paradigms that systematically vary reward types (e.g., psychological versus material), intensity, salience, and predictability. These designs will allow for a more precise characterization of the spatiotemporal dynamics underlying reward expectancy, including representational (e.g., RBP) and oscillatory (e.g., PAC) mechanisms.*"

In the Results section (lines 322-344 on pages 10-11), we added the findings of supplementary analyses to further verify the PAC effect when participants were approaching rewards: "*Additionally, we found that the PAC strength is different among the pre-ERZ, pre-PR, and pre-EDZ periods ($F_{(2,16)}$ = 8.72, p = 0.006; Figure 3D). Further analysis showed that the theta-gamma PAC effect was stronger during the pre-ERZ period than during the pre-PR (0.02 ± 0.09, $t_{(8)}$ = 3.01, p = 0.017) and pre-EDZ (0.01 ± 0.04, $t_{(8)}$ = 3.62, p = 0.007) periods.*"

In the Figure 3 (page 11), we added a new panel, Figure 3D, demonstrating the difference in PAC strength among pre-ERZ, pre-PR, and pre-EDZ conditions.

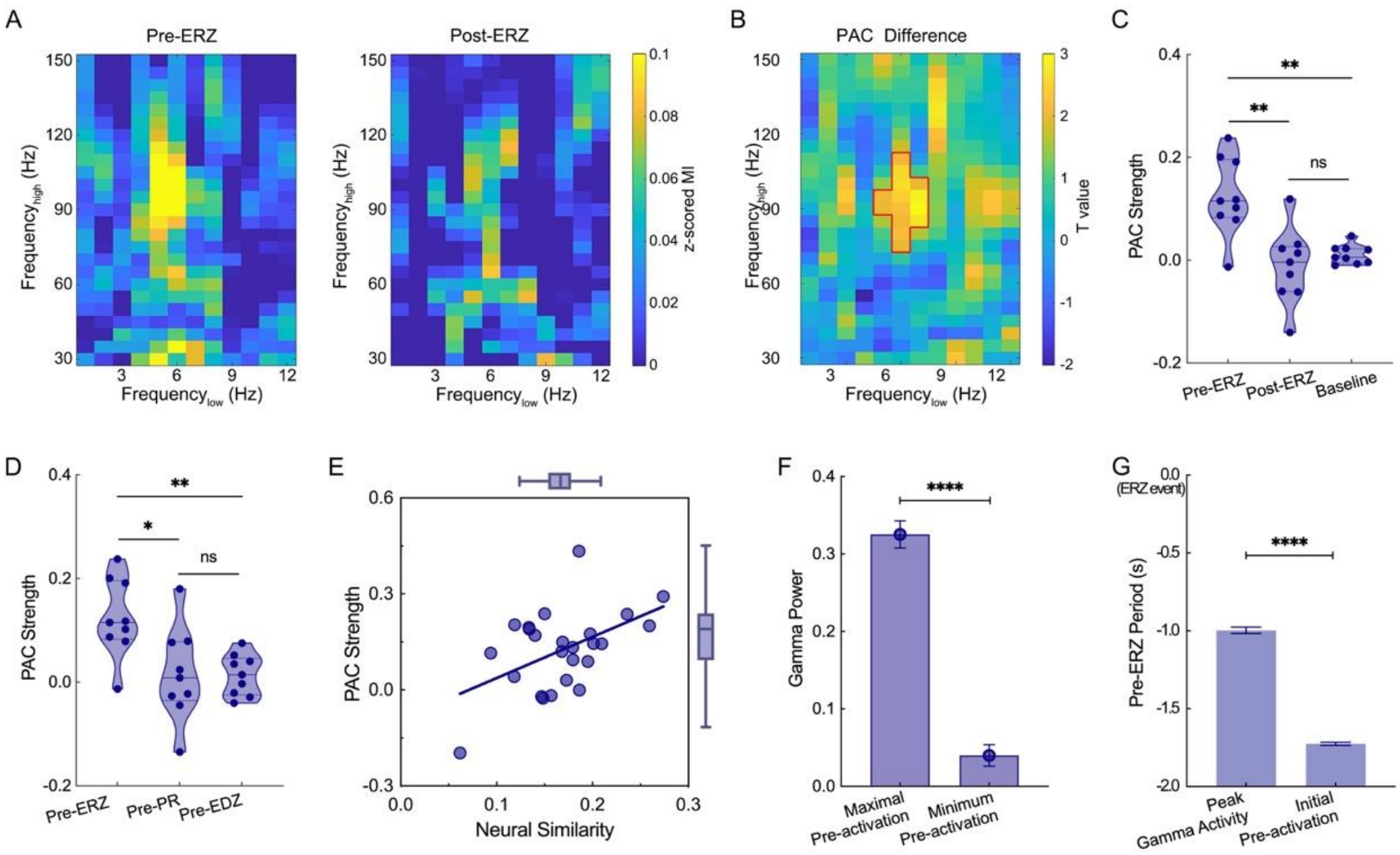

Figure 3. Theta-gamma PAC and its relationship to RBP pre-activation in the AIC before entering reward zones. (A) *Comodulograms illustrate the z-scored modulation index (MI) during the pre-ERZ (left) and post-ERZ (right) periods. ERZ, entering the reward zone.* ***(B)*** *Difference in phase-amplitude coupling (PAC) between pre-ERZ and post-ERZ periods. Red contours indicate a $frequency_{low}$-$frequency_{high}$ cluster showing significantly enhanced PAC during pre-ERZ relative to post-ERZ periods.* ***(C)*** *Average PAC values within the $frequency_{low}$-$frequency_{high}$ cluster from panel B for individual participants in the pre-ERZ, post-ERZ, and baseline conditions. Each violin plot indicates one condition, and each dot within violin plots indicates one participant. **, 0.005 ≤ p < 0.01; ns, no significant difference.* ***(D)*** *Average PAC values within the identified cluster from panel B for individual participants in the pre-ERZ, pre-PR, and pre-EDZ conditions. Each violin plot indicates one condition, and each dot within violin plots indicates one participant. *, 0.01 ≤ p < 0.05; **, 0.005 ≤ p < 0.01; ns, no significant difference.* ***(E)*** *The PAC strength positively correlate with the neural similarities of pre-ERZ periods relative to reward-specific brain patterns (RBP) across contacts. Each sphere indicates*

one recording contact, and the blue line indicates the fitted line. Blue box plots indicate the distribution of PAC strength or neural similarities. ***(F)*** *Gamma power showing PAC effects is higher at the time bins with maximal RBP pre-activation than those with minimum pre-activation. Error bar indicates SEM. ****, $p < 0.001$.* ***(G)*** *Peak gamma activity showing PAC effects occur later than initial RBP pre-activation. Y axis indicates the time course relative to pre-ERZ periods, and time 0 indicates the onset of rewards. Error bar indicates SEM. ****, $p < 0.001$.*

In the Methods section (lines 1198-1201 on page 26), the practice session was documented in details. *"Before the experiment, participants completed a practice session in a similar virtual maze. This session was designed to fully familiarize participants with both maze navigation (e.g., walking straight and turning) and the experimental procedure (e.g., picking up the reward upon its display), thereby minimizing potential practice effects in the main task.*"

R2.2. The use of the hippocampus as a control region is helpful to demonstrate regional differences between AIC and medial temporal lobe signals. However, the absence of comparable effects in the hippocampus should be interpreted with caution. The hippocampus is itself strongly implicated in spatial navigation, temporal coding, and theta-gamma coupling in other task contexts, and null effects here may reflect task demands, electrode coverage, or signal characteristics rather than a true functional dissociation. As such, the hippocampal comparison supports regional differentiation but does not by itself establish the specificity of reward expectancy of the AIC findings. Explicitly acknowledging this limitation in the discussion would help setting the scope of the conclusions.

Response:

Thank you for this insightful perspective on the interpretation of our hippocampal findings. We agree that caution is warranted when interpreting null results, and that the absence of certain effects in the hippocampus does not establish a strict functional dissociation.

We have performed a sanity check of iEEG signal in the hippocampus by analyzing theta activities during spatial navigation and decision making. The result showed pronounced theta activities during navigation and enhanced theta power during decision making, which are consistent with previous electrophysiological studies in hippocampus (*Ekstrom et al., Hippocampus, 2005; Watrous et al., Elife, 2018; Kaplan et al., Cogn Neurosci, 2020*). Specifically, we identified low-frequency oscillations (LFOs) in both the AIC and hippocampus using the Multiple Oscillations Detection Algorithm (MODAL) and the Fitting Oscillations & One Over F (FOOOF) algorithm (see Supplementary Text 1 and Supplementary Figure 16). The hippocampus exhibited a broader spectral profile than the AIC, with peaks spanning the theta and alpha bands that were dynamically modulated by behavioral context, aligning with the observation of a wider frequency range and greater inter-individual variability compared to rodents (*Bohbot et al., Nat Commun, 2017; Watrous et al., Elife, 2018; Mao, Neurosci Bull, 2023*). We further investigated difference in low-frequency power between pre- and post-event (ERZ or EDZ) periods. Higher-frequency theta activity (6-12 Hz) increased during movement (pre-ERZ and pre-EDZ periods), while lower-frequency theta activity (3-6.5 Hz) enhanced during decision-making (post-EDZ periods). These findings align with previous studies and reveal functionally distinct theta rhythms in the human hippocampus during virtual navigation (*Ekstrom et al., Hippocampus, 2005; Watrous et al., Elife, 2018; Kaplan et al., Cogn Neurosci, 2020*). These results are now added to Supplementary Text 2 and Supplementary Figure 17.

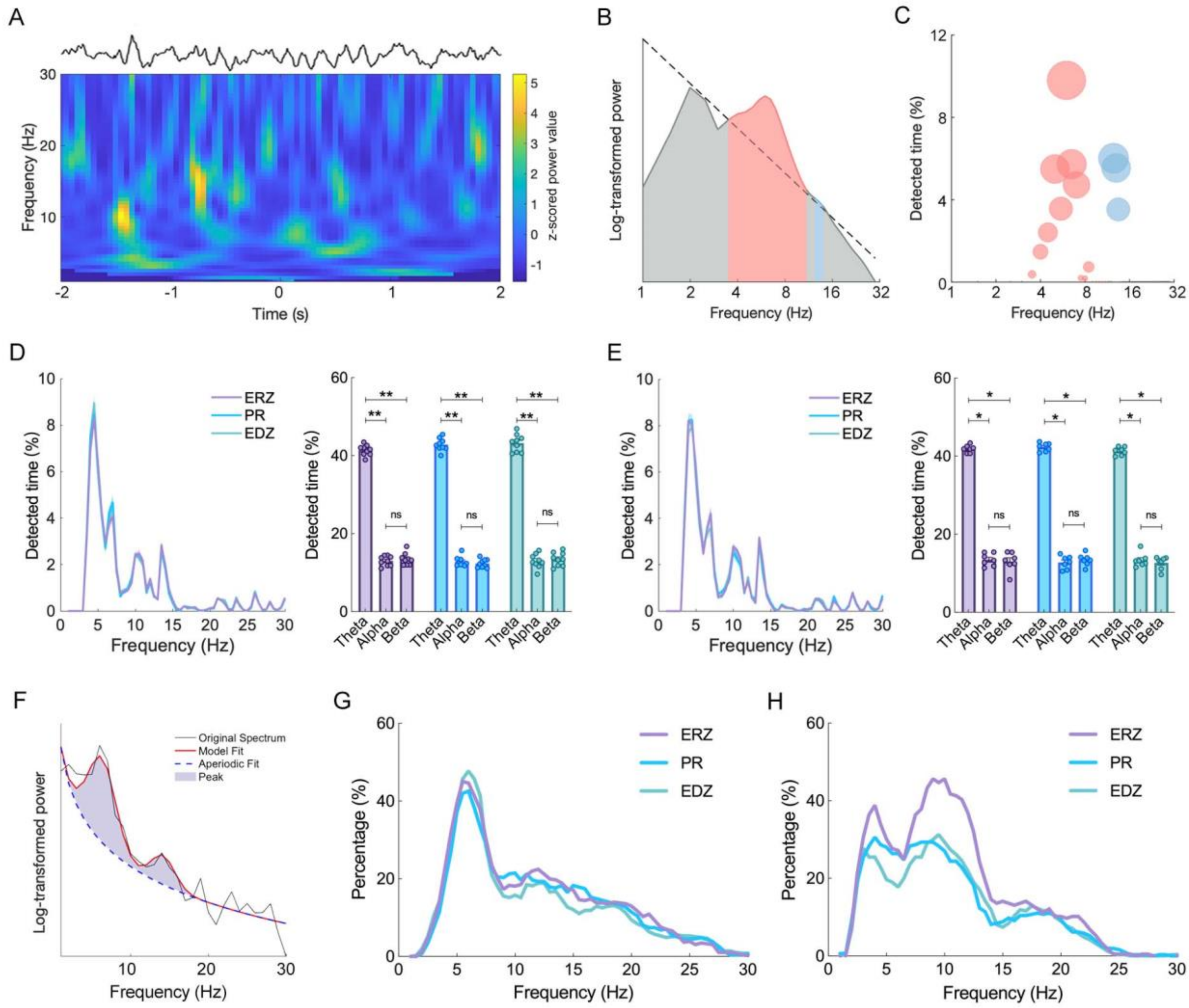

Supplementary Figure 16. Spectral characterization of low-frequency oscillations in the AIC and hippocampus during virtual navigation. (A-C) *An example trial from a recording contact in the right anterior insular cortex (AIC) of participant #16.* ***(A)*** *Raw trace and its time-frequency spectrum in the example trial. Time 0 indicates the timepoint of entering the reward zone.* ***(B)*** *Two narrow-band oscillations (3.5-11 Hz indicated by pink; 12.5-14 Hz indicated by light blue) are identified as contiguous frequencies exceeding the fitted line (dashed line) using the Multiple oscillations detection algorithm (MODAL).* ***(C)*** *Prevalence of oscillations at each frequency from the trial shown in panel B. The larger the sphere, the higher the percentage of detected time of the corresponding frequency.* ***(D-E)*** *Population-level oscillation prevalence in AIC (D) and hippocampal (E) contacts. Left: Percentage of time periods during which oscillatory activities are detected separately for each frequency. Right: Proportion of time periods for each detected oscillation band (theta: 3.5-7.5 Hz; alpha: 8-12 Hz; beta: 12.5-30 Hz). Rings represent individual participant, and error bars indicate one standard deviation (SD) from the mean. *, 0.01⩽p<0.05; **, 0.005⩽p<0.01; ns, no significance.* ***(F)*** *Example spectral decomposition using the Fitting Oscillations & One Over F (FOOOF) model. Narrow-band oscillations (light purple) are identified in the residual flattened spectrum after subtracting the aperiodic component (dashed line).* ***(G-H)*** *Distribution of spectral peak frequencies identified by FOOOF across experimental conditions in the AIC (G) and hippocampus (H). Each color denotes one experimental condition. ERZ, enter the reward zone; PR, picking up the reward; EDZ, enter the decision zone.*

Representational similarity analysis (RSA) for hippocampal data, however, reveals a more nuanced picture which we wish to clarify, and we have revised the Discussion to provide a balanced interpretation. We have

presented evidence of hippocampal role in reward processing and observed reward-specific brain pattern (RBP) and its pre-activation in the hippocampus. Critically, this hippocampal RBP pre-activation occurred earlier than its counterpart in the anterior insular cortex (AIC). This temporal precedence is consistent with the established role of the hippocampus in encoding and retrieving reward-context associations (*Bein et al., Neurosci Biobehav Rev, 2023; John et al., Hippocampus, 2001*), a process that logically precedes the integration of reward memory and contextual cues coordinated by the AIC. This temporal dynamic is a key point already elaborated in the manuscript (Figure 2 on page 8; lines 224-227 on page 7; lines 275-276 on page 9).

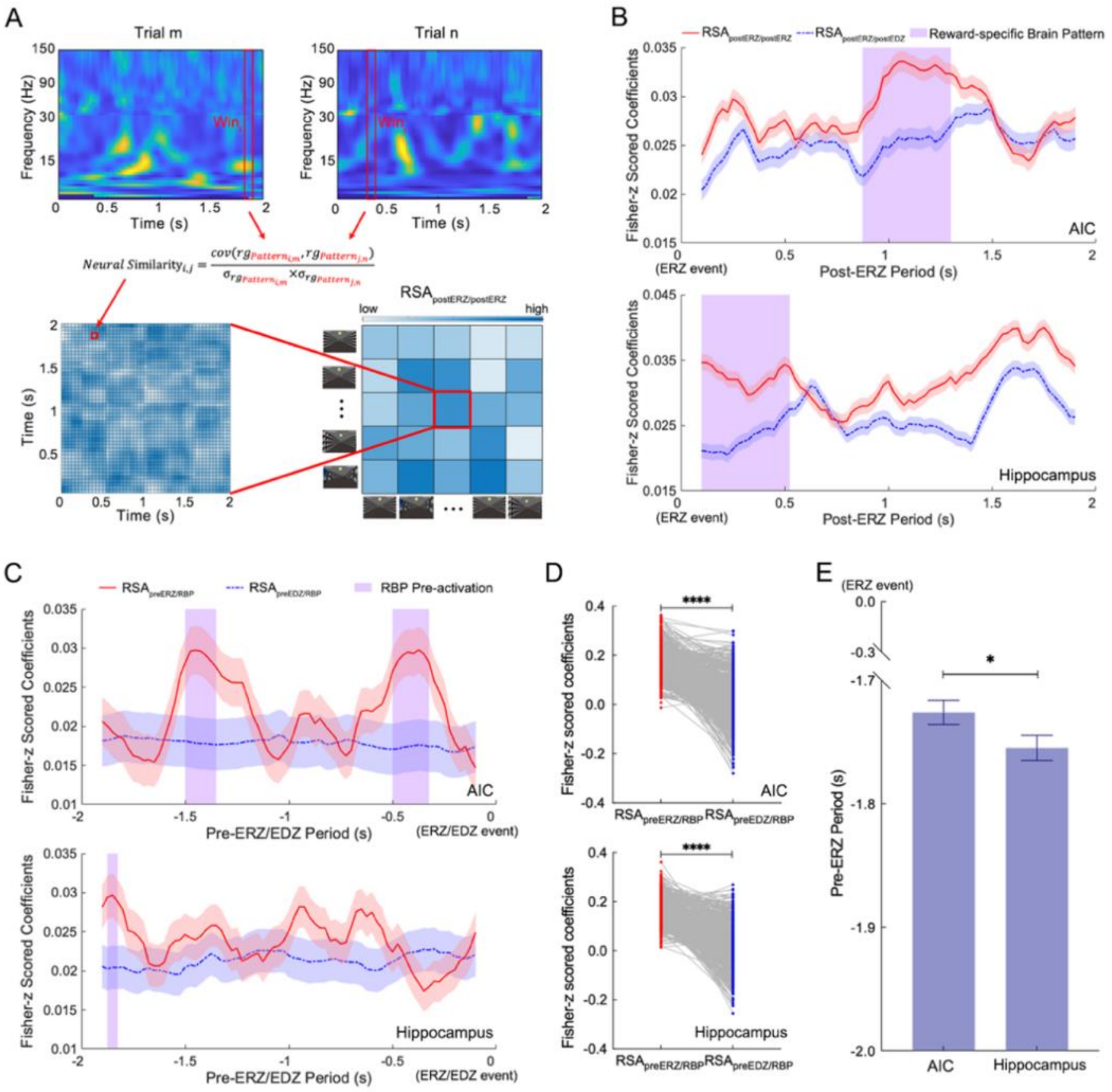

***Figure 2. Pre-activation of RBPs before entering reward zones in the AIC and hippocampus. (A)** Scheme of representational similarity analysis (RSA) to compute reward-specific brain patterns (RBPs). Representational similarity matrix (bottom left) illustrates the neural pattern similarities of the spectrum within each time window between different trials (top). Representational similarity matrix of $RSA_{postERZ/postERZ}$ (bottom right) displays the categorical information structure of viewing reward at different reward zones (on the central or two lateral roads). **(B)** Time-resolved category specificity reveals RBPs in the anterior insular cortex (AIC, top) and hippocampus (bottom). Each line represents time courses of $RSA_{postERZ/postERZ}$ (red; mean ± SEM) or $RSA_{postERZ/postEDZ}$ (blue). Purple shading indicates time windows carrying RBPs (i.e., $RSA_{postERZ/postERZ}$ > $RSA_{postERZ/postEDZ}$). X-axis denotes time course relative to post-ERZ periods, and time 0 indicates the onset of rewards (i.e., ERZ events). ERZ, entering the reward zone; EDZ, entering the decision zone. **(C)** Pre-activation of RBPs before entering the reward zones in the AIC (top) and hippocampus (bottom). Each line represents time-resolved similarity trajectories for $RSA_{preERZ/RBP}$ (red; mean ± SEM) or $RSA_{preEDZ/RBP}$ (blue). Purple shading highlights pre-ERZ time windows for RBP pre-activation (i.e., $RSA_{preERZ/RBP}$ > $RSA_{preEDZ/RBP}$). X axis indicates time course relative to pre-ERZ or pre-EDZ periods, and time 0 indicates ERZ or EDZ event. **(D)** Different magnitude of RBPs pre-activation between $RSA_{preERZ/RBP}$ (red) and $RSA_{preEDZ/RBP}$ (blue) in the AIC (top) and hippocampus (bottom).*

*Dots represent individual trials. ****, p< 0.001.* ***(E)*** *The RBP pre-activations in the AIC occur later than those in the hippocampus. Y axis indicates time course relative to pre-ERZ periods, and time 0 indicates the onset of rewards. Note that there is a discontinuity in the y-axis to enhance visualization. Error bar indicates SEM. *, 0.01* $\leqslant p < 0.05$.

Phase-amplitude coupling (PAC) between LFOs and gamma activity is a fundamental organizing principle of hippocampal neural dynamics (*Axmacher et al., Proc Natl Acad Sci USA, 2010; Daume et al., Nature, 2024; Vivekananda et al., Hippocampus, 2021*). However, direct evidence linking specific PAC configurations to spatial navigation in humans remains limited. One potential reason is that multiple processes are involved in the navigation process (e.g., spatial mapping, decision-making, and reward processing), which makes it difficult to isolate condition-specific PAC effects. As an exploratory analysis, we computed PAC strength between two LFO bands (2-4 Hz and 7-9 Hz) and four gamma sub-bands (30-55 Hz, 60-85 Hz, 90-115 Hz, and 120-150 Hz) identified in prior human navigation studies (*Bohbot et al., Nat Commun, 2017; Watrous et al., Elife, 2018; Mao, Neurosci Bull, 2023*). Hippocampal PAC varied across task conditions, with distinct frequency pairs showing enhanced or suppressed PAC effects depending on different conditions (i.e., pre-ERZ, post-ERZ, pre-PR, pre-EDZ, and post-EDZ) which may due to different cognitive demands. These findings suggest that the hippocampus is continuously engaged during virtual spatial navigation. However, its activity is reconfigured across frequency pairs depending on cognitive demands. These data are now added to Supplementary Text 2 and Supplementary Figure 17.

In summary, we have refined our interpretation of the hippocampal findings. We now explicitly state that the comparison involving the hippocampus illustrates its distinct temporal and functional engagement in reward processing, rather than a mere absence of involvement. While our data highlight the precise, temporally coordinated role of the AIC in orchestrating reward expectancy, they do not preclude a complementary, context-associative role for the hippocampus. Additionally, we acknowledge the inherent limitations of clinical iEEG studies, including sparse spatial sampling and potential coverage bias. We thank the reviewer for prompting this important clarification, which has strengthened the nuance and scope of our conclusions.

Manuscript Revisions:

In the Discussion section, we have revised as following:

At lines 875-882 on page 21, "*Concurrently, the enhancement of PAC prior to reward onset was observed in the AIC, but not the hippocampus. This dissociation may be indicative of distinct dopaminergic mechanisms involved*[61]*. The hippocampus appears to prioritize novelty detection via transient dopamine bursts, which enhance theta-gamma PAC during exploratory navigation (see Supplementary Text 2; Supplementary Figure 17). In the context of our paradigm, which relies on well-established and predictable rewards, such a mechanism would be less engaged. Conversely, the AIC may integrate predictable reward information through more sustained dopaminergic modulation*[61,63,64]*, thereby supporting the stable anticipatory theta-gamma coordination observed in this study.*"

At lines 883-989 on pages 21-22, "*Taken together, our population-level findings reveal a temporally ordered and correlated interplay between representational pre-activation and oscillatory coordination within human AIC for reward expectancy. However, it should be noted that the observed correlation and temporal sequence between PAC and RBP pre-activation exhibited heterogeneity across individual recording contacts. Moreover, the extremely limited number of unrewarded trials prevented a statistically meaningful comparison between reward and no-reward conditions. To mitigate this limitation, we employed trial-matched EDZ periods as a control condition for ERZ, effectively accounting for confounding factors such as visual stimulus processing, spatial anticipation, and action preparation. This approach enabled maximal*

control for general task-related and motor-preparatory activity, thereby isolating neural signals specific to reward expectancy. Future studies incorporating more extensive electrode coverage and causal manipulations will be essential to clarify the consistency and directionality of this relationship."

In the Supplementary Information, we added the text and figure corresponding to theta activity and PAC in the hippocampus.

"***Supplementary Text 2. Theta activity and phase-amplitude coupling (PAC) in the hippocampus during virtual spatial navigation***

Previous research has demonstrated that rodent hippocampus exhibits prominent theta activity (5-10 Hz) during locomotion, with both frequency and power increasing as the running speed increases[6]. In humans, however, theta oscillations during virtual navigation are more heterogeneous, spanning a broader range (2-9 Hz)[4,7,8]. While human theta power is indeed sensitive to movement (e.g., higher during locomotion than stillness)[8-10], it is also influenced by attention and memory demands[8,11]. For instance, theta power increases significantly during decision-making, and correlates with decision speed and choice accuracy[12,13]. Notably, the dominant theta frequency can shift depending on task demands[5], with higher-frequency theta activity potentially supporting tasks requiring concentrated spatial attention[5]. This frequency-specific modulation suggests that distinct cognitive processes may be supported by different theta sub-bands.

In the present study, we investigated low-frequency power dynamics by computing z-scored power (1-12 Hz in 0.5-Hz steps; see Methods) for time windows surrounding ERZ and EDZ events. To explore differences in low-frequency power between pre- and post-event (ERZ or EDZ) periods, we employed a multi-level linear regression analysis on the z-scored power values, accounting for variances at both the participant and contact levels for each time-frequency bin. Bins with a p-value < 0.05 were considered significant, and adjacent significant bins were clustered together[14]. For a given cluster, a summed t-value across all bins was assigned as the statistical value of that cluster. To compute the significance level of each cluster, we implemented a cluster-based permutation analysis[15]. Within each permutation test, we randomly shuffled condition labels (i.e., pre- or post-event period) and repeated the multi-level linear regression analysis. This generated a set of surrogate clusters, from which the cluster with the maximum statistical value was retained. This procedure was repeated 500 times, generating 500 surrogate clusters. Empirical clusters exceeding the 95th percentile of the surrogate distribution were considered significant (p < 0.05). For ERZ event, higher-frequency theta activity (9-12 Hz) was enhanced during pre-ERZ (i.e., moving) relative to post-ERZ (i.e., stillness) periods (-1.662 to -1.078 seconds; p = 0.009; Supplementary Figure 17A), reflecting locomotion-related theta activity [6,9,10]. A parallel analysis of EDZ events similarly showed enhanced higher-frequency theta (6-12 Hz) during movement (pre-EDZ periods; -1.592 to -1.136 seconds; p = 0.001; Supplementary Figure 17B), further confirming locomotion-related theta modulation[6,9,10]. Additionally, lower-frequency theta activity (3-6.5 Hz) increased after EDZ events (1.284 to 1.902 seconds; p = 0.007), suggesting decision-related neural correlates[12]. These findings reveal functionally distinct theta rhythms in the human hippocampus during virtual navigation, aligning with prior work[5,8-10,12]. Specifically, higher-frequency theta activity associated with locomotion and lower-frequency theta activity linked to decision process.

PAC between LFOs and gamma activity represents a fundamental organizing principle of hippocampal neural dynamics, enabling the integration of information across multiple temporal scales[16,17]. Accumulating evidence from rodents has established that theta-gamma PAC plays a critical role in spatial coding and path integration during navigation[16], and invasive intracranial electroencephalography (iEEG) studies in humans have similarly implicated hippocampal PAC in supporting working and long-term memory[18-20], with different PAC configurations supporting distinct cognitive processes. However, direct evidence for task-dependent modulation of hippocampal PAC during virtual spatial navigation in humans remains limited. This gap may

be accounted for by two factors. First, spatial navigation involves a complex interplay of cognitive processes, including spatial mapping, decision-making, and salience processing, which makes it difficult to isolate PAC signatures associated with specific cognitive subcomponents[5]*. Second, the human hippocampus exhibits considerable heterogeneity in its low-frequency spectral profile compared to the more stereotyped theta rhythm observed in rodents. Rather than displaying a narrow-band oscillation concentrated around a fixed frequency, human hippocampal LFOs show marked interindividual variability*[1,4,5]*. Some participants exhibit clear spectral peaks in the 7-9 Hz range during memory-guided navigation in virtual environments, while others show peaks in the 2-4 Hz range*[5]*. This broad-band and bimodal distribution may reflect the fact that human hippocampal oscillations are not exclusively dedicated to spatial processing, but also participate in internally generated activities, contextual construction, and cognitive control processes*[2]*. For each experimental condition, we computed average PAC strength between two LFO bands of interest (2-4 Hz and 7-9 Hz) and four gamma sub-bands (30-55 Hz, 60-85 Hz, 90-115 Hz, and 120-145 Hz) which were reported in previous studies*[4,5,9]*. Our analyses revealed that hippocampal PAC hippocampal PAC varied dynamically across conditions (Supplementary Figure 17C). Prior to entering the reward zones, PAC between 7-9 Hz and 60-85 Hz was significantly decreased, suggesting a reorganization of information integration as participants approached the reward zone. Upon entering the reward zone, we observed enhanced PAC in two distinct frequency pairs—2-4 Hz with 30-55 Hz and 7-9 Hz with 120-145 Hz—pointing to a shift in oscillatory coupling configurations during reward acquisition and contextual updating. Immediately prior to reward collection, PAC between 7-9 Hz and 30-55 Hz was decreased, potentially reflecting reduced information integration or network reconfiguration prior to action execution. During the pre-EDZ period, PAC between 2-4 Hz and 120-145 Hz was reduced while coupling between 7-9 Hz and 30-55 Hz was enhanced, suggesting that different low-frequency rhythms differentially regulate distinct information channels during path selection and strategy evaluation. Upon entering the decision zone, PAC between 7-9 Hz and 30-55 Hz was decreased, possibly reflecting inhibition or resetting of task-related network activity following decision completion.*

Collectively, these findings support the view that the hippocampus is continuously engaged in virtual spatial navigation, though its specific frequency composition undergoes dynamic reconfiguration according to changing cognitive demands. The dynamic reorganization of PAC likely constitutes a key mechanism through which the hippocampus integrates and allocates information across navigation, decision-making, and reward processing. Future studies that refine the dissection of cognitive subcomponents are essential to determining the specific functional contributions of each frequency pair. Moreover, higher-density sampling along the longitudinal axis of hippocampus may reveal regional specialization[14]*."*

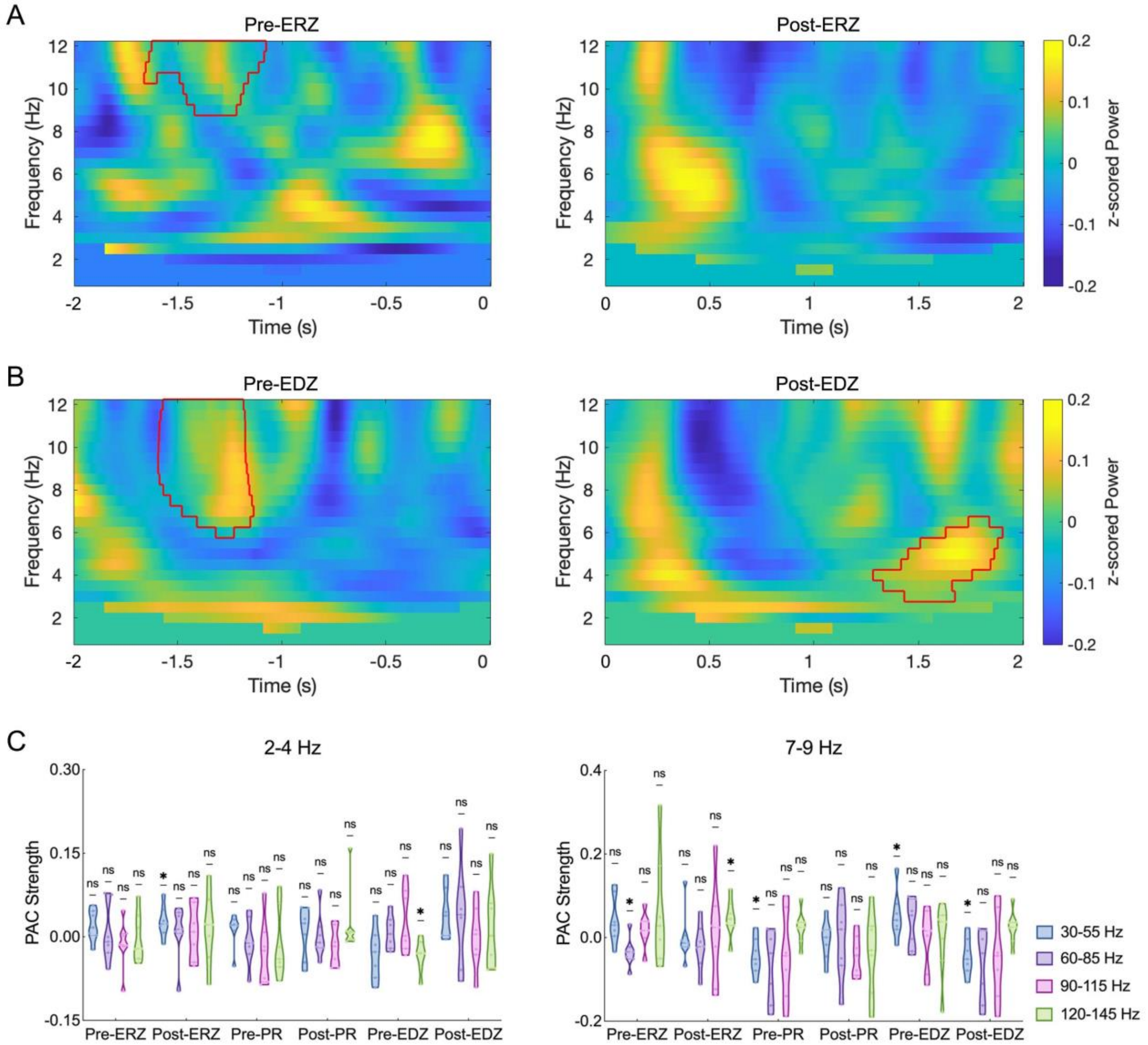

Supplementary Figure 17. Theta activity and phase-amplitude coupling (PAC) in the hippocampus. Relate to Figure 3. (A) *Theta activity before (left) and after (right) entering the reward zone (ERZ). Red contours indicate a time-frequency cluster showing significantly increased theta power. X-axis denotes time courses relative to ERZ periods, and time 0 indicates the onset of rewards.* ***(B)*** *Theta power before (left) and after (right) entering the decision zone (EDZ). Red contours indicate a time-frequency cluster showing significantly enhanced theta activity. X-axis denotes time courses relative to EDZ periods, and time 0 indicates the onset of decision cues.* ***(C)*** *Average PAC strength between two low-frequency bands (2-4Hz, left; 7-9Hz, right) and four gamma sub-bands. Each violin represents one frequency pair, dots represent individual participants. *, $0.01 \leq p < 0.05$; ns, not significant.*

R2.3. RBPs are operationalized as neural patterns showing higher representational similarity between reward epochs (across locations) than between reward and non-reward epochs (reward–reward > reward–non-reward). These RBP estimates are subsequently used as a reference template to identify time windows preceding reward onset that exhibit increased similarity ("pre-activation"). The approach is technically careful, and the authors implement several important controls, including for spatial location (central vs lateral arms) and for temporal autocorrelation (excluding pre- and post-reward activity from the same trial). Moreover, RBP pre-activation is shown stronger for pre-ERZ than for pre-EDZ periods

(Fig. 2D, S1), supporting contextual specificity relative to decision-related processing. However, how can we be certain that no other processes which are highly likely present (at pre-ERZ and post-ERZ periods) and which definitely have an influence on AI (and hippocampal) activity, like attention, salience, or motor preparation/execution are not being captured by that definition?
More specifically, the definition of RBPs relies on a post-ERZ contrast and subsequent pre-ERZ pre-activation, but ERZ is tightly coupled to an imminent button press (PR), raising the possibility that the identified RBPs partially reflect response urgency or motor preparation rather than a reward-specific representation. Since the data includes already PR events (and corresponding iEEG epochs), the authors could compute PR–PR similarity and compare ERZ–ERZ similarity against ERZ–PR (and/or PR–EDZ) similarity using the same analysis pipeline (multilevel regression and cluster-based permutation). Demonstrating that ERZ-defined RBPs do not simply recapitulate PR-locked patterns (i.e., limited overlap in significant time windows, reduced ERZ–PR similarity relative to ERZ–ERZ) would substantially strengthen the interpretation that RBPs are reward-specific rather than reflecting generic motor- (i.e., keypress-) related dynamics.

Response:
Thank you for raising this important methodological point regarding the specificity of reward-specific brain patterns (RBPs). We agree that the temporal proximity between the ERZ and PR events necessitates careful consideration of whether RBPs could reflect motor preparation rather than reward-specific encoding.
The present study selected EDZ as the primary control condition because it aligns with ERZ in core cognitive and motor demands; both events involve visual stimulus processing, attention engagement, salience detection, and preparation of a button press. It is important to note that EDZ event occurs in a distinct spatial location and is cued by a neutral geometric figure rather than a reward signal. The objective of this design was to identify neural patterns associated with reward expectation, controlling for general processes such as attention, salience, visual processing, and motor preparation. The finding that RBP pre-activation is more pronounced before ERZ than before EDZ supports its specificity to reward expectancy beyond these shared components.
In order to directly address the possibility that RBPs might reflect PR-related motor dynamics, a supplementary analysis was conducted by taking PR as a control condition. This analysis compared neural pattern similarity within post-ERZ epochs ($RSA_{postERZ/postERZ}$) against $RSA_{postERZ/prePR}$. The results demonstrate that within a 0.975-1.35 seconds window following reward onset, neural similarity is significantly higher for reward-viewing pairs than for reward-viewing pairs and reward-picking pairs ($p_{corrected} = 0.023$). These findings are consistent with the RBP time window identified by the original contrast ($RSA_{postERZ/postERZ} > RSA_{postERZ/postEDZ}$) and provide further evidence that the identified RBP do not merely reflect motor preparation linked to the upcoming button press. We have revised the manuscript by adding results of this new analysis and the corresponding figure (Supplementary Figure 1).

Manuscript Revisions:
In the Results section (lines 228-235 on page 7), we added the results from the new analysis. "*Furthermore, to rule out the possibility that RBPs identified in the AIC were confounded by PR-related motor preparation, we conducted additional control analyses using the RSA approach described above. Specifically, we compared neural pattern similarity for reward-viewing pairs ($RSA_{postERZ/postERZ}$) against that for reward-viewing versus reward-picking pairs ($RSA_{postERZ/prePR}$). Neural activity within 0.975-1.3 seconds following reward onset showed significantly higher $RSA_{postERZ/postERZ}$ than $RSA_{postERZ/prePR}$ ($p_{corrected} = 0.023$; Supplementary Figure 1). The observation aligns with the contrast between $RSA_{postERZ/postERZ}$ and $RSA_{postERZ/postEDZ}$ reported earlier, further*

supporting that the identified RBPs reflect reward-specific representation, rather than motor preparation for the impending button press."

In the Supplementary Information, we added a figure corresponding to the additional analysis.

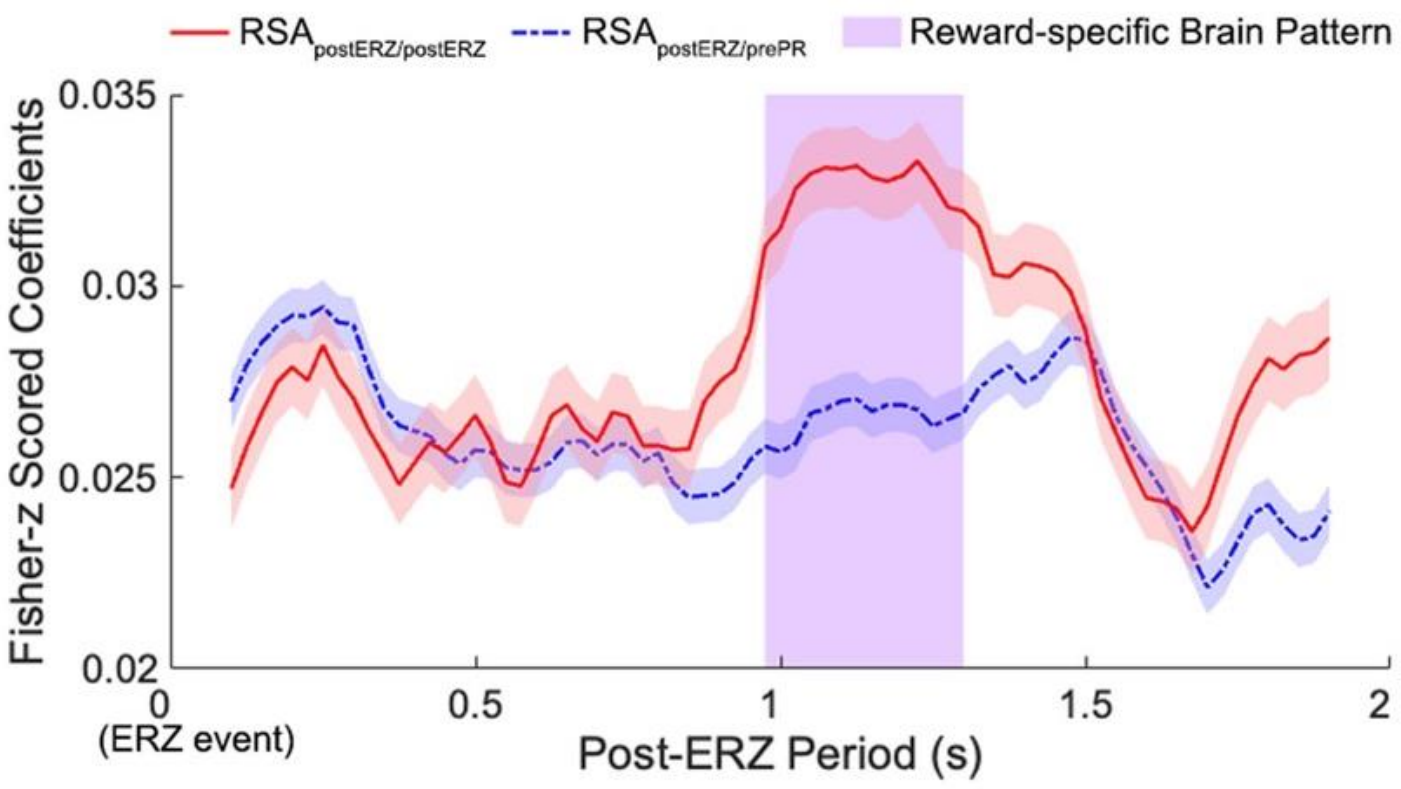

Supplementary Figure 1. Reward-specific brain pattern (RBPs) in the anterior insular cortex. Related to Figure 2. *Time-resolved category specificity reveals RBPs during reward viewing. Each line represents time courses of $RSA_{postERZ/postERZ}$ (red; mean ± SEM) or $RSA_{postERZ/prePR}$ (blue). Purple shading indicates time windows carrying RBPs (i.e., $RSA_{postERZ/postERZ} > RSA_{postERZ/prePR}$). X-axis denotes time courses relative to post-ERZ periods, and time 0 indicates the onset of rewards (i.e., ERZ events). ERZ, entering the reward zone; PR, picking up the reward.*

R2.4. The main PAC effect is estimated as a difference between pre-ERZ and post-ERZ periods, with a significant theta-gamma PAC cluster in AIC that is stronger before reward onset. While this is an interesting result, the reliance on a pre–post contrast introduces ambiguity regarding whether the effect reflects a selective enhancement during reward expectancy versus a relative reduction/disruption of coupling after ERZ due to sensory transients, motor execution, or task-completion dynamics. The inclusion of a randomly sampled baseline helps, but the baseline segments are not matched for navigation state or motor readiness, limiting the ability to attribute the effect to expectancy rather than to differences in behavioral state across pre vs post epochs. A stronger test would be to compare pre-ERZ PAC directly against pre-EDZ or pre-PR periods (anticipatory epochs with different cognitive demands), or examine the time-resolved PAC dynamics within the pre-ERZ interval to assess whether coupling is specifically expressed during expectancy versus ramping with proximity to action/reward onset.

Response:

Thank you for these insightful comments. We fully agree that relying on a pre-post contrast can introduce ambiguity about whether the observed effect reflects a selective enhancement during reward expectancy or a general reduction in coupling after the ERZ event due to sensory, motor, or task-completion processes. Although our original design included comparisons of pre-ERZ activity with both post-ERZ and a randomly sampled baseline, the incomplete matching of navigation states between these epochs limited a definitive interpretation. As you suggested, to test more directly whether the enhanced PAC is specific to reward anticipation rather than reflecting a post-ERZ reduction due to sensory/motor/task-completion dynamics, we have now directly compared PAC strength during the pre-ERZ period against that during the pre-EDZ and pre-PR periods—anticipatory epochs with different cognitive demands. The result further confirmed the enhanced PAC effect during pre-ERZ period. We have revised the Results section by adding this new analysis.

Manuscript Revisions:

In the Results section (lines 322-344 on pages 10-11), we added the findings of supplementary analyses to further verify the PAC effect when participants were approaching rewards: "*Additionally, we found that the PAC strength is different among the pre-ERZ, pre-PR, and pre-EDZ periods ($F_{(2,16)}$ = 8.72, p = 0.006; Figure 3D). Further analysis showed that the theta-gamma PAC effect was stronger during the pre-ERZ period than during the pre-PR (0.02 ± 0.09, $t_{(8)}$ = 3.01, p = 0.017) and pre-EDZ (0.01 ± 0.04, $t_{(8)}$ = 3.62, p = 0.007) periods.*"

In the Figure 3 (page 11), we added a new panel, Figure 3D, demonstrating the difference in PAC strength among pre-ERZ, pre-PR, and pre-EDZ conditions.

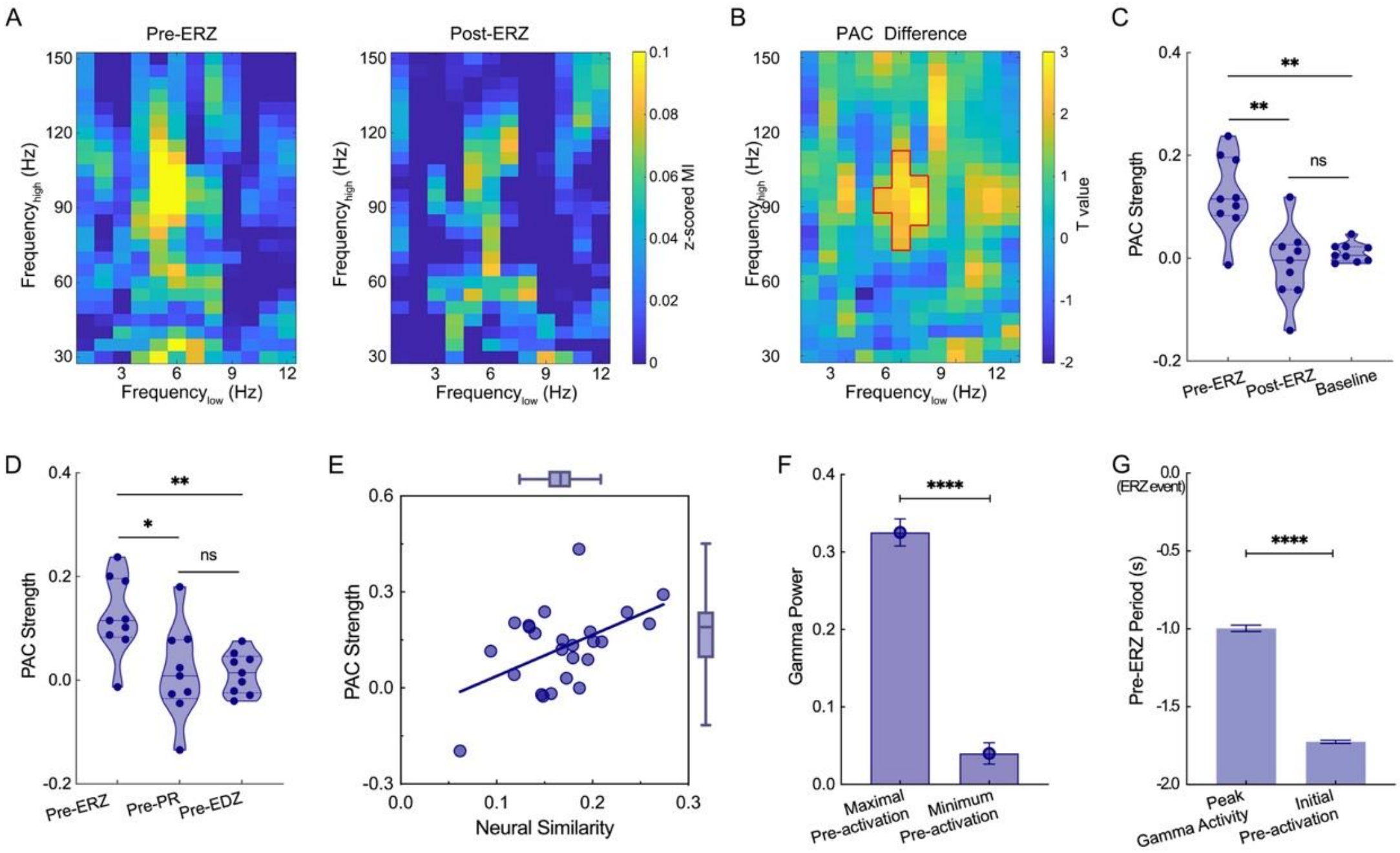

Figure 3. Theta-gamma PAC and its relationship to RBP pre-activation in the AIC before entering reward zones. (A) *Comodulograms illustrate the z-scored modulation index (MI) during the pre-ERZ (left) and post-ERZ (right) periods. ERZ, entering the reward zone.* ***(B)*** *Difference in phase-amplitude coupling (PAC) between pre-ERZ and post-ERZ periods. Red contours indicate a $frequency_{low}$-$frequency_{high}$ cluster showing significantly enhanced PAC during pre-ERZ relative to post-ERZ periods.* ***(C)*** *Average PAC values within the $frequency_{low}$-$frequency_{high}$ cluster from panel B for individual participants in the pre-ERZ, post-ERZ, and baseline conditions. Each violin plot indicates one condition, and each dot within violin plots indicates one participant. **, 0.005 ≤ p < 0.01; ns, no significant difference.* ***(D)*** *Average PAC values within the identified cluster from panel B for individual participants in the pre-ERZ, pre-PR, and pre-EDZ conditions. Each violin plot indicates one condition, and each dot within violin plots indicates one participant. *, 0.01 ≤ p < 0.05, **, 0.005 ≤ p < 0.01; ns, no significant difference.* ***(E)*** *The PAC strength positively correlate with the neural similarities of pre-ERZ periods relative to reward-specific brain patterns (RBP) across contacts. Each sphere indicates one recording contact, and the blue line indicates the fitted line. Blue box plots indicate the distribution of PAC strength or neural similarities.* ***(F)*** *Gamma power showing PAC effects is higher at the time bins with maximal RBP pre-activation than those with minimum pre-activation. Error bar indicates SEM. ****, p < 0.001.* ***(G)*** *Peak gamma activity showing PAC effects occur later than initial RBP pre-activation. Y axis indicates the time course relative to pre-ERZ periods, and time 0 indicates the onset of rewards. Error bar indicates SEM. ****, p < 0.001.*

R2.5. The observed association between RBP pre-activation and theta-gamma PAC is intriguing and makes the authors suggest a link between representational and oscillatory processes in AIC. However,

this relationship is assessed via correlations across contacts and by inspecting the temporal ordering of those processes. As such, inferences about functional coupling at the trial level are limited, since cross-contact correlations could reflect variance related to other factors (e.g., signal quality or electrode placement) rather than a direct mechanistic relationship, and of course the finding that one event precedes another does not imply that the first drives the second. The authors should either use more cautious language or demonstrated trial-wise associations within contacts to strengthen the interpretation that PAC specifically coordinates or refines reward-related representations.

Response:

Thank you for raising this crucial point regarding the functional relationship between RBP pre-activation and theta-gamma PAC. We agree that cross-contact correlations and temporal precedence alone are insufficient to establish trial-level mechanistic coupling or causality, and that a more cautious interpretation is warranted.

To directly address your concern, we performed additional within-contact, trial-wise analyses. The key outcomes are:

1) Correlation between PAC strength and RBP pre-activation level: While a positive correlation between PAC strength and RBP pre-activation level was observed across contacts, this relationship was not uniform. Specifically, significant positive correlations across trials were found in 15 out of 25 contacts (Supplementary Figure 6), indicating that the group-level effect is supported by a majority of sites but also reveals notable contact-specific variability.
2) Temporal sequence of theta-gamma PAC and RBP pre-activation: The peak gamma activity associated with PAC occurred significantly later than the onset of RBP pre-activation at the group level. This temporal pattern was highly consistent across trials, observed in 24 out of 25 individual contacts (Supplementary Figure 7). While this robust sequence is consistent with RBP pre-activation preceding PAC enhancement, we fully acknowledge that temporal order does not imply causality.

Manuscript Revisions:

In the Abstract section (lines 31-32 on page 2), we revised as follows: "*This pre-activation was followed by phase-amplitude coupling (PAC) between theta oscillations and gamma activity, with coupling strength positively correlated with the pre-activation level across contacts*."

In the Results section, we updated the text to include additional within-contact, trial-wise analyses.

At lines 386-393 on page 12, "*Subsequently, we investigated the relationship between PAC dynamics and RBP pre-activation preceding reward onset. The result showed that the PAC strength was positively correlated with the level of RBP pre-activation across all AIC contacts (r = 0.49, p = 0.012, Pearson's correlation; Figure 3E). To further verify the relation between PAC strength and RBP pre-activation level, we compute the correlation across trials within individual contacts. A significant positive correlation was observed in 15 out of 25 contacts (Supplementary Figure 6), a proportion significantly exceeding chance level (i.e., 5%; p =* 5.97×10^{-14}*; Binomial test). It indicates that the group-level effect was supported by the majority of recording contacts, while also revealing some contact-specific variability.*"

At lines 399-435 on pages 12-13, "*Additionally, we examined the temporal relationship between the peak theta-gamma PAC effect and the onset of RBP pre-activation. The peak PAC effect for each pre-ERZ period was defined as the emergence of peak gamma activity (75-110 Hz) with the maximal amplitude at the preferred PAC phase (5-7 Hz; see Methods). Across all trials and contacts, the peak gamma activity occurred markedly later than the initial RBP pre-activation (-1.00 ± 0.02 seconds [mean ± SEM] vs. -1.70 ±* 9.7×10^{-3} *seconds, p < 0.001; Wilcoxon matched-pairs signed rank test; Figure 3G), with a mean time lag of 0.73 ±*

0.02 seconds. The coefficient of variation (CV, 85.77%) indicated a high dispersed distribution of the time lag. In individual contacts, the peak PAC effect was significantly delayed relative to RBP pre-activation across trials in 24 out of 25 contacts (ps < 0.001), with no significant difference in the remaining contact (p = 0.697; Supplementary Figure 7)."

In the Discussion section, we have explicitly acknowledged the heterogeneity across contacts and adopted more circumspect language.

At lines 682-683 on page 18, "*Subsequent observation revealed enhanced theta-gamma PAC, the peak of which occurred following the rise of RBP pre-activation in most recording contacts.*"

At lines 835-863 on pages 20-21, "*Collectively, these findings indicate a potential sequence in which preactivated RBPs provide the representational content ("what"), and subsequent theta-gamma PAC in the AIC contributes to the temporal structure ("when" and "how") of reward expectancy.*"

At lines 883-989 on pages 21-22, "*Taken together, our population-level findings reveal a temporally ordered and correlated interplay between representational pre-activation and oscillatory coordination within human AIC for reward expectancy. However, it should be noted that the observed correlation and temporal sequence between PAC and RBP pre-activation exhibited heterogeneity across individual recording contacts. Moreover, the extremely limited number of unrewarded trials prevented a statistically meaningful comparison between reward and no-reward conditions. To mitigate this limitation, we employed trial-matched EDZ periods as a control condition for ERZ, effectively accounting for confounding factors such as visual stimulus processing, spatial anticipation, and action preparation. This approach enabled maximal control for general task-related and motor-preparatory activity, thereby isolating neural signals specific to reward expectancy. Future studies incorporating more extensive electrode coverage and causal manipulations will be essential to clarify the consistency and directionality of this relationship.*"

In the Supplementary Information, we added two new supplementary figures to support these points.

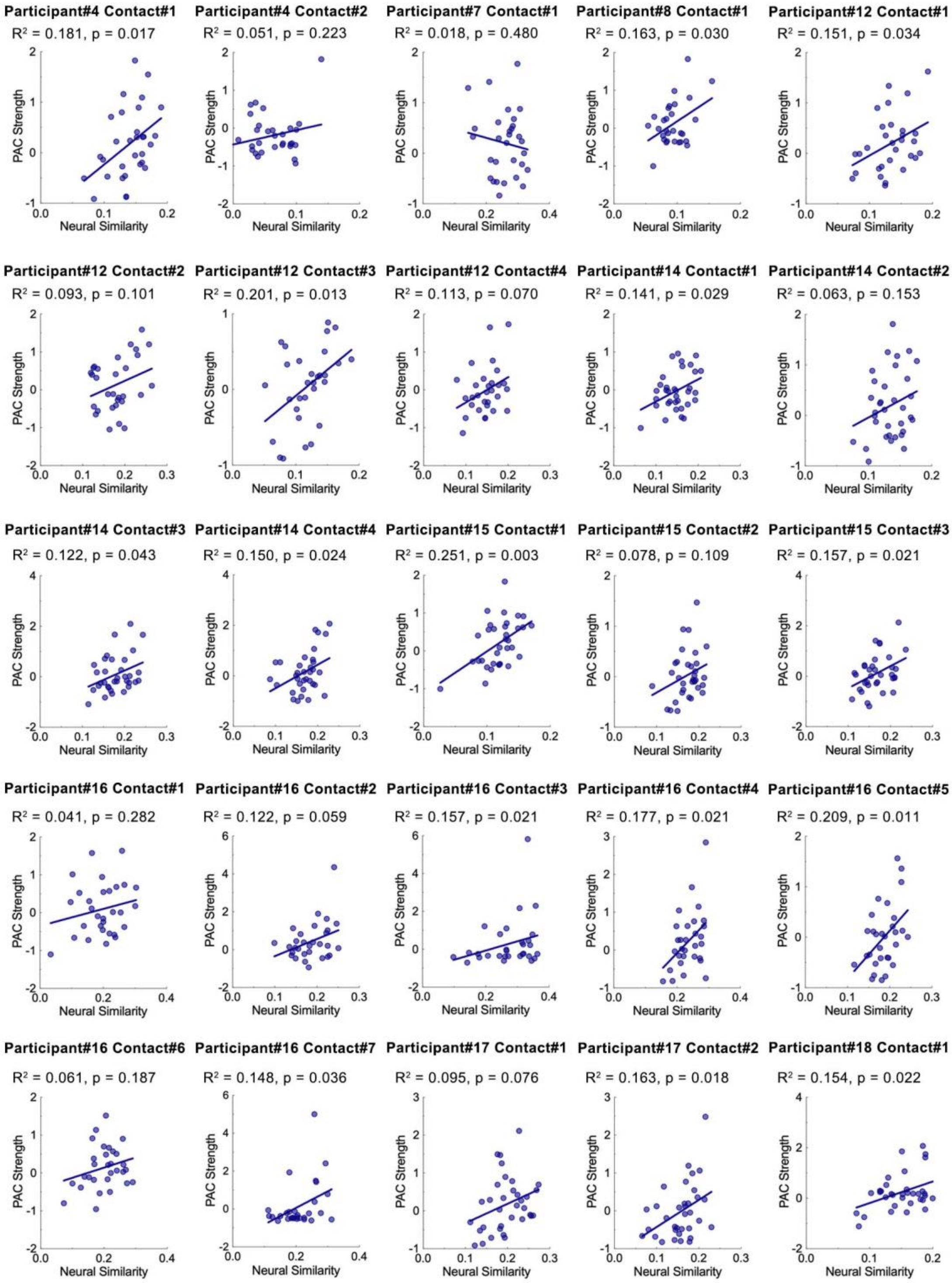

Supplementary Figure 6. Theta–gamma PAC exhibits positive correlation with RBP pre-activation level. Relate to Figure 3. *A significant positive correlation between the strength of phase-amplitude coupling (PAC) and the pre-activation level of reward-specific pattern (RBP) was observed in majority (15/25) of anterior insular contacts. Each panel represents one recording contact; blue dots indicate individual trials.*

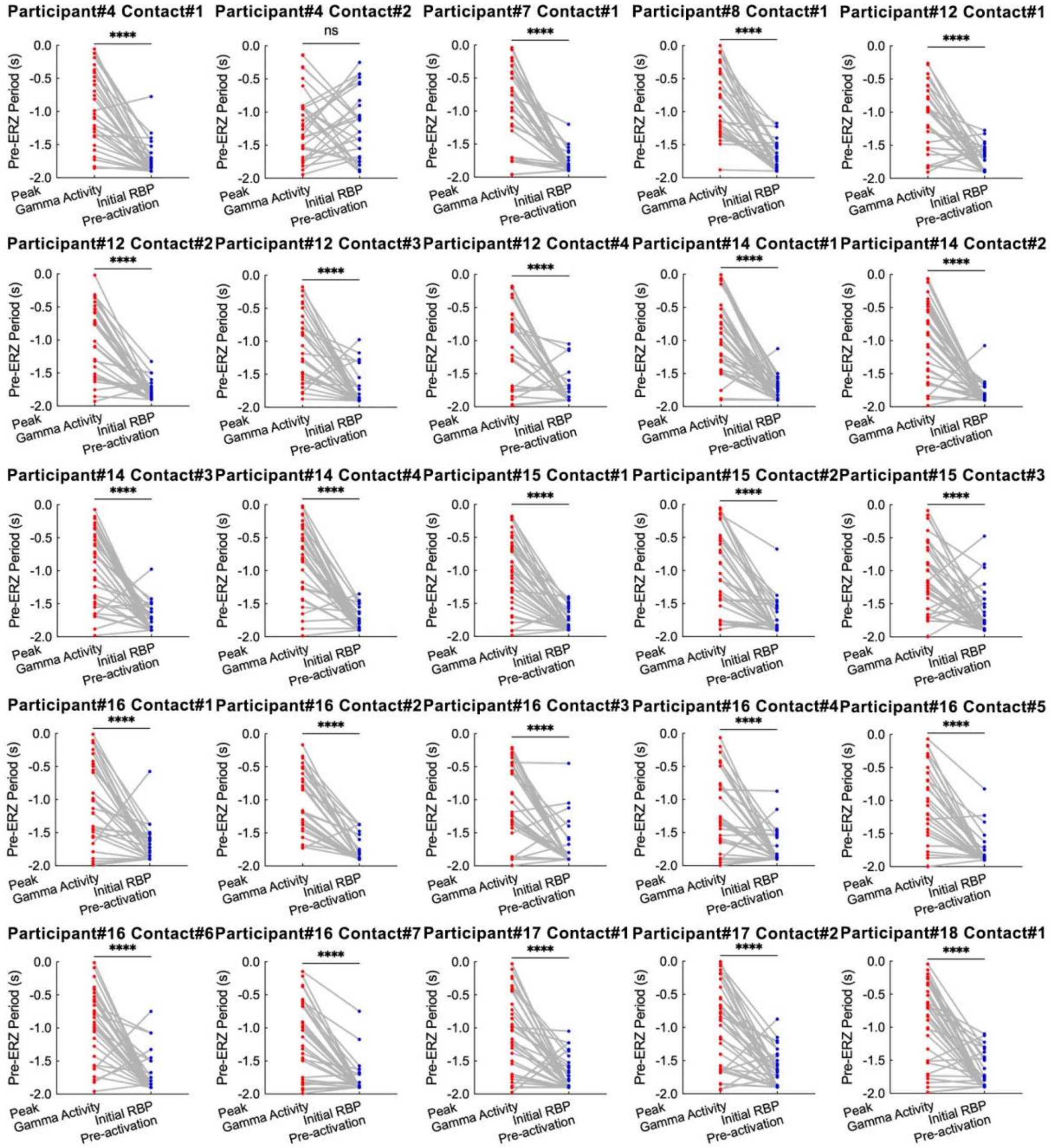

Supplementary Figure 7. Temporal sequence of RBP pre-activation and PAC effect at the trial level. Related to Figure 3. *Trial-wise time points of peak gamma activity (red) and initial RBP pre-activation (blue) within each contact. Each panel represents one contact, and dots indicate individual trials. Y-axis shows time relative to pre-ERZ periods; time 0 represents reward onset. ****, $p < 0.001$; ns, not significant.*

R2.6. Significant PPLE is observed in only a subset of AIC contacts (9/25), indicating spatial heterogeneity in the effect. While such sparsity is expected in intracranial recordings, it limits the strength of region-level claims and makes it difficult to infer a systematic reward-expectancy mechanism without additional controls.

Response:

Thank you for this thoughtful comment. We agree that the observation of PPLE in only a subset of AIC contacts (9/25; 36%) reflects spatial heterogeneity in its expression. Such sparsity is common and expected in intracranial recordings due to the precise spatial scale of neurophysiological signal relative to electrode placement. Nevertheless, PPLE occurred selectively during the pre-ERZ periods, rather than during the pre-EDZ and pre-PR periods. The incidence was significantly above chance level ($p \leq 10^{-6}$, Binomial test), indicating a non-random effect. Furthermore, this prevalence is markedly higher than the previously reported prevalence of phase precession in humans (35/1,074 neurons; *Qasim et al., Cell 2021*), further supporting the physiological relevance of our observations.

We acknowledge that the heterogeneity of contacts constrains any broad claims regarding the AIC as a functionally homogeneous region. Therefore, we have reframed our interpretation to propose that the PPLE represents a detectable and significant mechanism within the AIC circuitry that contributes to reward expectancy, without implying it is ubiquitously expressed in every neuron or subregion. Future studies with higher-density coverage are needed to map its precise spatial topography.

Manuscript Revisions:

In the Results section (line 482-484 on page 14), we added the following: "*The prevalence of PPLE significantly exceeded the chance level (i.e., 5%) at both the contact and participant levels (ps* $\leq 10^{-6}$*; Binomial test), confirming the neurophysiological relevance of PPLE.*"

In the Discussion section (lines 1050-1053 on page 23), we acknowledged the following limitation: "*Nevertheless, the observation that the PPLE was present in 9 out of 25 contacts implies the spatial specificity corresponding to the AIC's known fine-grained functional architecture*[74]*. Further investigation employing higher-density electrode coverage is warranted to map the precise topography of PPLE and clarify its relationship with functional subdomains of the AIC.*"

R2.7. Moreover, in terms of the "functional relevance of PPLE", both PPLE strength and behavioral performance evolve monotonically across navigation rounds, raising the possibility that their association reflects a general time-on-task or practice effect rather than reward-specific expectancy learning. Demonstrating that PPLE predicts behavioral performance beyond the effect of round number, or adopting a more cautious framing of PPLE as reflecting dynamic phase coordination during task engagement, would better align the interpretation with the evidence presented.

Response:

Thank you for raising this essential methodological point about the potential confounding factor of practice effects. We agree that a careful distinction between reward-specific expectancy processes and general time-on-task or practice effects is required.

Although we implemented several experimental controls to mitigate practice effects, including an extensive practice session prior to the main task to ensure all participants were fully familiarized with the procedure, we acknowledge that the monotonic trends observed across navigation rounds warrant further statistical consideration.

To address this concern, we conducted additional control analyses. First, we confirmed that PPLE strength showed no significant correlation with average response time across participants, either before (r = -0.25, p = 0.52) or after controlling for navigation rounds (r = -0.18, p = 0.66). This indicates that PPLE does not simply track overall response speed or stable individual differences in performance. More critically, to test whether the association between PPLE strength and the degree of behavioral improvement is independent of general task

progression, we performed a partial correlation analysis between PPLE strength and the magnitude of behavioral improvement, controlling for navigation rounds. The result showed a strong positive effect size (*r* = 0.66), though it did not reach conventional statistical significance (*p* = 0.076). Given the modest sample size and the preliminary nature of this analysis, we interpret this result with caution. The result suggests a tendency for PPLE to relate to behavioral improvement beyond mere round progression. However, we acknowledge that a practice-related component cannot be fully ruled out based on the current evidence.

We appreciate your suggestion, which has helped us refine our analyses and present a more cautious and evidence-aligned interpretation of the findings.

Manuscript Revisions:

In the Results section, we added the results of supplementary correlation analyses described above.

> At lines 594-672 on pages 17-18, "*To address the potential confounding factor of general practice, we performed a partial correlation analysis controlling for navigation rounds. The association remained substantial in effect size (r = 0.66), yet it was not statistically significant (p = 0.076). While this indicates the relationship may not be solely attributed to time-on-task effects, the interpretation requires caution due to the small number of participants. Additionally, participants with a detectable PPLE (n = 6) showed numerically higher but statistically comparable behavioral improvement to those without (0.45 ± 0.18 [mean ± SD] vs. 0.16 ± 0 .23, p = 0.17; Wilcoxon matched-pairs signed rank test; Figure 5C). These results suggest that the strength, rather than the presence, of PPLE is related to behavioral improvement. Further studies with larger sample sizes are needed to investigate the functional relevance of PPLE strength.*
>
> *To determine whether the PPLE was specifically related to dynamic behavioral optimization rather than overall response speed, we tested its correlation with average RT across participants. The PPLE strength showed no significant correlation with average RT, either before (r = -0.25, p = 0.52; Supplementary Figure 15C) or after controlling for navigation rounds (r = -0.18, p = 0.66). This dissociation indicates that the PPLE strength tracks trial-by-trial behavioral improvement, rather than stable individual differences in general performance.*"

In the Discussion section, we have revised our interpretations and acknowledged the limitations more explicitly.

> At lines 1085-1092 on page 24, we revised the manuscript as follows: "*We observed that the strength of PPLE was correlated with the magnitude of trial-by-trial behavioral improvement. This suggests that PPLE reflects a precise phase-tuned timing mechanism engaged during reward expectancy. Future studies with larger sample size and greater statistical power are needed to determine whether the PPLE specifically tracks reward-specific processes or is related to general practice effects. Nevertheless, the relationship between PPLE strength and the progressive optimization of responses towards upcoming rewards highlights a potential role for phase-tuned oscillatory dynamics in optimizing behavioral performances*[1,2,4]."
>
> At lines 1099-1102 on page 24, we outline key directions for further studies. "*Future research should employ multidimensional reward paradigms that systematically vary reward types (e.g., psychological versus material), intensity, salience, and predictability. These designs will allow for a more precise characterization of the spatiotemporal dynamics underlying reward expectancy, including representational (e.g., RBP) and oscillatory (e.g., PAC) mechanisms.*"

In the Methods section (lines 1198-1201 on page 26), the practice session was documented in details. "*Before the experiment, participants completed a practice session in a similar virtual maze. This session was designed to fully familiarize participants with both maze navigation (e.g., walking straight and turning) and the experimental procedure (e.g., picking up the reward upon its display), thereby minimizing potential practice effects in the main task.*"

R2.8. Although this does not detract from the presence of theta–gamma coupling, demonstrating that the PAC effect persists after accounting for gamma power fluctuations would further strengthen the interpretation that coupling reflects coordinated oscillatory dynamics rather than amplitude-driven effects. I mention this because PAC metrics are sensitive to changes in high-frequency amplitude, and the manuscript reports increased gamma power during epochs associated with reward approach.

Response:
Thank you for this valuable methodological point. To assess whether the observed theta-gamma PAC is independent of gamma amplitude fluctuations, we performed a correlation analysis at the single-contact level between averaged gamma amplitude and PAC strength. In vast majority of contacts (23/25) the correlations were not significant, indicating that the PAC effect is not trivially explained by changes in gamma amplitude. We have revised the Results section by integrating results from this new analysis.

Manuscript Revisions:
In the Results section (lines 378-385 on page 12), we added the following paragraph, "*To rule out the possibility that the observed enhancement in theta-gamma PAC was confounded by fluctuations in gamma-band amplitude, we conducted an additional control analysis. For each recording contact, we assessed the correlation between PAC strength and averaged gamma amplitude (75-110 Hz) during the pre-ERZ period across trials. In 23 out of 25 contacts, gamma amplitude and PAC strength were not significantly correlated (ps ≥ 0.15, Pearson's correlation; Supplementary Figure 5). This finding indicates that the reported theta-gamma PAC effect is largely independent of variations in gamma amplitude, supporting the interpretation that it reflects genuine oscillatory coordination rather than an amplitude-driven artifact.*"
In the Supplementary Information, we have added:

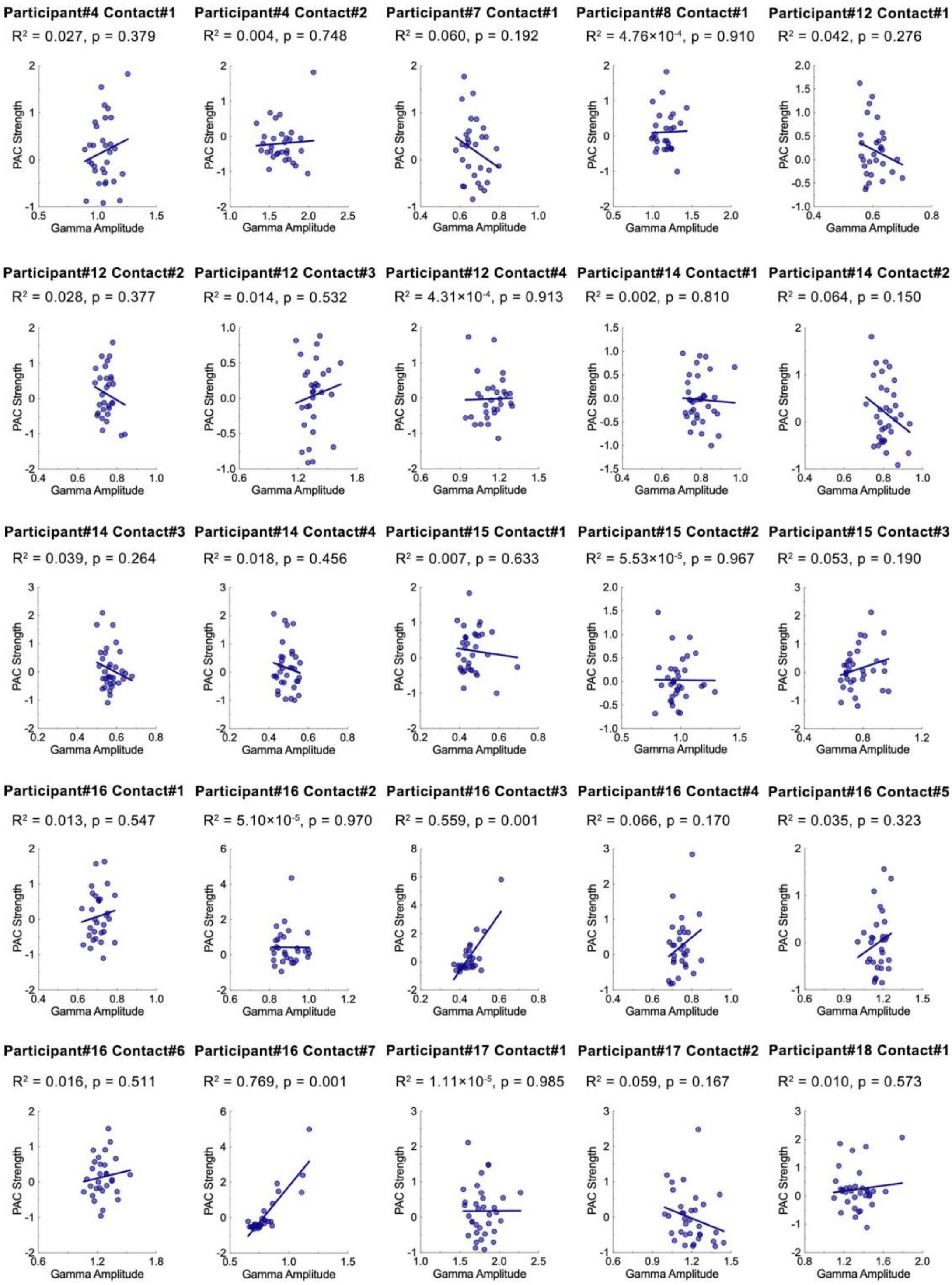

Supplementary Figure 5. Theta–gamma PAC exhibits substantial independence from gamma-band amplitude fluctuations. *Across most anterior insular contacts (23/25), theta-gamma phase-amplitude coupling (PAC) strength was not significantly correlated with gamma-band amplitude. Each panel represents one recording contact; blue dots indicate individual trials.*

R2.9. Figure 1 (perhaps 1B and 1D) could be adjusted to reflect the events of interest ERZ, EDZ, and PR.

Response:

Thank you for this constructive suggestion. We have revised Figure 1 (page 6) to better illustrate the events of interest.

Manuscript Revisions:

In Figure 1B, blue arrows now indicate the precise spatial locations of the ERZ and EDZ events, along with the approximate location of the PR event.

In Figure 1D, we have revised descriptive labels above the corresponding task-stage schematics: "ERZ (Central Corridor)", "EDZ" and "ERZ (Right Arm)".

The figure legend has been updated as follows:

At lines 194-196 on page 6, we added "*Blue arrows indicate the precise spatial locations of the ERZ (entering the reward zone) and EDZ (entering the decision zone) events, and the approximate location of the PR (picking up the reward) event.*"

At line 200 on page 6, we removed "ERZ, entering the reward zone; PR, picking up the reward; EDZ, entering the decision zone."

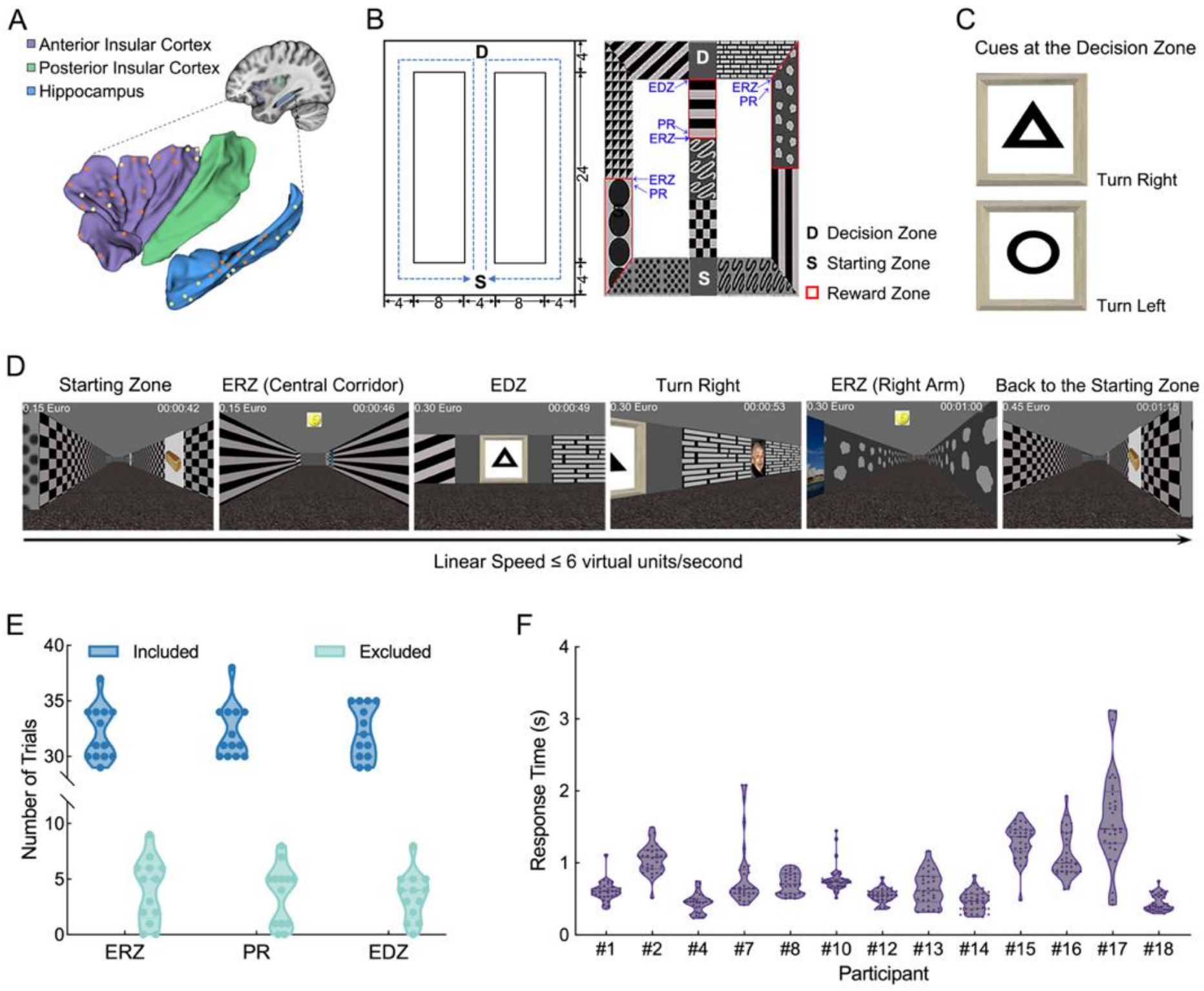

Figure 1. Contact locations, experimental paradigm, and behavioral performance. (A) *Spatial distribution of contacts in the anterior insular cortex (purple), posterior insular cortex (green), and hippocampus (blue). Each orange sphere indicates a contact in the right hemisphere, and each yellow sphere indicates a contact in the left hemisphere.* ***(B)*** *Overhead view of the T maze. Left: Diagram of the virtual maze. Each blue dashed line represents one navigation round. Right: T maze adorned with various patterns on its walls. The gray square marked with 'S' represents the starting zone where each navigation round begins and ends, while the gray square marked with 'D' represents the decision zone where participants are expected to choose the correct direction according to the cue. Areas circled with red lines represent*

reward zones, where participants are expected to collect gold coins by pressing the "Enter" button as soon as possible. Blue arrows indicate the precise spatial locations of the ERZ (entering the reward zone) and EDZ (entering the decision zone) events, and the approximate location of the PR (picking up the reward) event. ***(C)*** *Cues on the wall of the decision zone. Circle represents turning left, and triangle represents turning right.* ***(D)*** *An example navigation round from the first-person perspective in the maze.* ***(E)*** *Number of trials are similar across conditions for both included (blue) and excluded (green) trials. Note that there is a discontinuity in the y-axis to enhance visualization. Each dot within violin plots indicates one participant.* ***(F)*** *Response time for picking up rewards at the reward zone on the central corridor. Each violin plot indicates one participant, and each dot within violin plots indicates one navigation round.*

R2.10. There are typos here and there across the manuscript (e.g., "To test this hypothesise" in page 7) that should be corrected.

Response:
Thank you for your careful reading and for pointing out the typographical errors throughout the manuscript. We have thoroughly proofread the entire text and corrected all identified spelling, grammar, and phrasing issues.

Manuscript Revisions:
At line 212 on page 7, "To test this hypothesise" has been corrected to "To test this hypothesis".

Point-by-point response

Referee #1 (Remarks to the Author):

The authors have addressed all of my previous comments and included additional analyses and figures that significantly strengthen the manuscript and offer important mechanistic insights into the reported effects. I have only a few minor comments regarding the revised manuscript.

R1.5. The correlation between RBP and reaction times appears quite strong, whereas there is no significant correlation with PAC. This is an interesting observation; however, I would refrain from interpreting this as evidence against a simple linear cascade in the absence of causal manipulation of the temporal sequence. Given that PAC estimation is generally more sensitive to methodological choices and may exhibit higher variability than power-based measures (see van Driel et al., 2015), it is possible that detecting PAC–behavior relationships requires greater statistical power (lines 585–590, page 17).

Response:

We appreciate your thoughtful comment. We have carefully read van Driel et al. (2015) and agree that phase-amplitude coupling (PAC) measures are more susceptible to phase-clustering bias and noise than power-based measures. We have performed a series of methodological manipulation to eliminate the phase-clustering bias (lines 671-685 on page 26). First, we band-pass filtered the signals in narrow bands of 1 Hz width from 1 to 12 Hz in 1 Hz steps, and performed visual inspection to confirm their sinusoidal nature. Second, we computed the Modulation Index (MI; Tort et al, 2010), which is robust against phase-clustering bias and noise (Driel et al., 2015; Hülsemann, Mareike J et al., 2019). Finally, we conducted permutation tests and z-transformation, which also eliminates phase-clustering bias (Driel et al., 2015).

Despite these methodological manipulations, the lack of a significant correlation between the strength of PAC and the magnitude of behavioral improvement – as reported in our manuscript – cannot be interpreted as direct evidence against a linear cascade in the absence of causal manipulation. To reflect this, we have modified the original claim and added the van Driel et al. (2015) to the bibliography. The interpretation is now more appropriately qualified.

Manuscript Revisions:

We have revised the Results section (lines 322-330 on page 10) accordingly. It now reads: "*We first examined how behavioral improvement related to RBP pre-activation level and PAC strength. The result showed that the magnitude of behavioral improvement showed a negative correlation with the average level of RBP pre-activation across participants ($r = -0.83$, $p = 0.006$; Pearson's correlation), but no significant correlation with average PAC strength ($r = -0.22$, $p = 0.57$; Supplementary Figure 15A and B). These results do not support a simple linear cascade in which representational pre-activation and cross-frequency coupling directly drive reward-directed behavioral improvement. Notably, the absence of a significant correlation with PAC should be interpreted with caution, as PAC estimates are more variable than power-based measures[53], potentially requiring greater statistical power to detect behavioral links.*"

R1.6. I am still unsure about the multilevel linear regression model. Could the authors please specify the dependent variable and the predictors included in the model? It would also be helpful to include the regression specification (e.g., model syntax), along with the beta coefficients and the variance explained (R^2).

Response:

Thank you for your careful reading. We have now fully specified the multilevel linear regression model as follows:

Dependent variable: single-trial RSA value
Fixed effect: cue condition (postERZ/postERZ vs. postERZ/postEDZ)
Random effects: by-participant intercepts and by-contact intercepts
Model syntax: RSA value ~ Cue + (1|Participant) + (1|Contact)

The coefficients (Estimate, SE tStat, pValue) for the fixed effect of Cue, as well as the conditional R^2 (lme.Rsquared.Ordinary), are now provided in Supplementary Data (coefficients_for_RSAlme.xlsx). Full time-bin-wise results are available in the supplementary file, and the beta coefficients and R^2 within the significant time clusters are highlighted in red.

Manuscript Revisions:

In the Methods section (lines 633-641 on pages 25-26), we elaborated the model syntax in detail. "*To assess the statistical difference between $RSA_{postERZ/postERZ}$ and $RSA_{postERZ/postEDZ}$, we conducted multi-level linear regression analysis for each time bin using fitlme with the following formula in MATLAB:*

RSA value ~ Cue + (1|Participant) + (1|Contact)

in which the single-trial RSA value was the dependent variable, and the cue condition (postERZ/postERZ vs. postERZ/postEDZ) was the fixed effect. Random effects included by-participant intercepts and by-contact intercepts."

In the Results section, we added the resource for beta coefficients and conditional R^2.

At line 133 on page 6, "*Significant RBPs emerged in both regions during reward viewing. In the AIC, neural activity within 0.875-1.3 seconds following reward onset showed significantly higher neural pattern similarity for reward viewing pairs ($RSA_{postERZ/postERZ}$) compared to reward viewing and non-reward viewing pairs ($RSA_{postERZ/postEDZ}$, $p_{corrected} = 0.047$; Figure 2B, top; see Supplementary Data).*"

At line 157 on page 7, "*During these time windows, the $RSA_{preERZ/RBP}$ values were significantly higher than the average $RSA_{preEDZ/RBP}$ values (Figure 2C, top; see Supplementary Data).*"

In the Supplementary Data, we added a dataset including beta coefficients and conditional R^2 (coefficients_for_RSAlme.xlsx).

R1.8. Thank you for providing the code and data. I did not run the scripts but briefly reviewed the Python code. I was unable to locate code related to the statistical analyses (in particular, the regression model mentioned above). Could the authors provide the code used to run the statistical analysis?

Response:

Thank you for pushing us to improve the transparency. We have now uploaded the statistical analysis codes to the public repository figShare (https://figshare.com/s/7d4ec0ac23271ca0549d), including RSAlme.m, PAClme.m, PhaseShift.py and PhaseShift_surr.py. We apologize for the earlier omission and appreciate your careful reading.

Referee #2 (Remarks to the Author):

R2.1. I appreciate the time and effort that the authors spent on this revision. I think the manuscript has improved substantially, the additional analyses help support their arguments and I appreciate the newly included cautionary statements which help contextualize their interpretations with respect to the

limitations of the study. The authors have addressed my comments and I recommend the manuscript for publication.

Response: Thank you for your positive assessment and recommendation. We are glad that you found the manuscript substantially improved and the additional analyses supportive of our arguments. We truly appreciate your recognition of our revisions.
